# Evolutionary origins of taro (*Colocasia esculenta*) in Southeast Asia

**DOI:** 10.1002/ece3.6958

**Published:** 2020-11-02

**Authors:** Ibrar Ahmed, Peter J. Lockhart, Esperanza M. G. Agoo, Kyaw W. Naing, Dzu V. Nguyen, Dilip K. Medhi, Peter J. Matthews

**Affiliations:** ^1^ Institute of Fundamental Sciences Massey University Palmerston North New Zealand; ^2^ Alpha Genomics Private Limited Islamabad Pakistan; ^3^ Biology Department De La Salle University Manila Philippines; ^4^ Vegetable and Fruit Research and Development Center Hlegu Myanmar; ^5^ Institute of Ecology and Biological Resources & Graduate University of Science and Technology Vietnam Academy of Science and Technology Hanoi Vietnam; ^6^ Department of Anthropology Gauhati University Guwahati India; ^7^ Department of Cross‐Field Research National Museum of Ethnology Osaka Japan

**Keywords:** Araceae, chloroplast DNA, cultivated, domestication, evolution, wild

## Abstract

As an ancient clonal root and leaf crop, taro (*Colocasia esculenta*, Araceae) is highly polymorphic with uncertain genetic and geographic origins. We explored chloroplast DNA diversity in cultivated and wild taros, and closely related wild taxa, and found cultivated taro to be polyphyletic, with tropical and temperate clades that appear to originate in Southeast Asia sensu lato. A third clade was found exclusively in wild populations from Southeast Asia to Australia and Papua New Guinea. Our findings do not support the hypothesis of taro domestication in Papua New Guinea, despite archaeological evidence for early use or cultivation there, and the presence of apparently natural wild populations in the region (Australia and Papua New Guinea).

## Graphical abstract

As an ancient clonal root and leaf crop, taro (*Colocasia esculenta*, Araceae) is highly polymorphic with uncertain genetic and geographic origins. We explored chloroplast DNA variation in wild and cultivated taros and other *Colocasia* species and found cultivated taro to be polyphyletic, with tropical and temperate clades originating in Southeast Asia. A third clade was found exclusively in wild populations from Southeast Asia to Australia and Papua New Guinea.

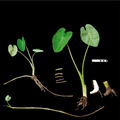

## 1 INTRODUCTION

Before the Columbian exchange of crops between the Americas and the Old World, *Colocasia esculenta* (L.) Schott (taro, Araceae) was the world's most widespread food crop, grown in tropical to temperate regions of Africa, Mediterranean, Asia, and Oceania (Grimaldi, 2016; Grimaldi et al., 2018; Matthews, 1991, 2006, 2014; Spriggs et al., 2012). Cultivated forms produce edible starchy mother corms, stolons or starchy side‐corms, and leaves (often to 1.5 m tall, with long petioles and broad peltate blades) (Matthews, 2004, 2010). Consensus is lacking on the full number of distinct *Colocasia* species (currently about 20), and new species continue to be discovered in Southeast Asia (Matthews, 2014). Early botanical records of other *Colocasia* species and wild populations of *C. esculenta* led to initial suggestions that taro originated as a natural species in the region of northeast India to Southeast Asia and was domesticated there (de Candolle, 1885; Matthews, 1991; Spier, 1951). In Oceania, taro has long been involved in debates on the origins of agriculture in New Guinea, the movements of people into Oceania, and cultural connections with Southeast Asia. Archaeological, archaeobotanical, and botanical findings provided circumstantial support for an independent, early‐ to mid‐Holocene domestication in New Guinea (Fullagar et al., 2006; Golson, 1989; Golson et al., 2017; Matthews, 1991). In order to investigate the possibility of domestication in New Guinea, the senior author surveyed wild taro populations in Papua New Guinea and northern Australia in 1985 (Matthews, 1991, 2014). Samples from this early survey were included in the present study.

**FIGURE 1:**
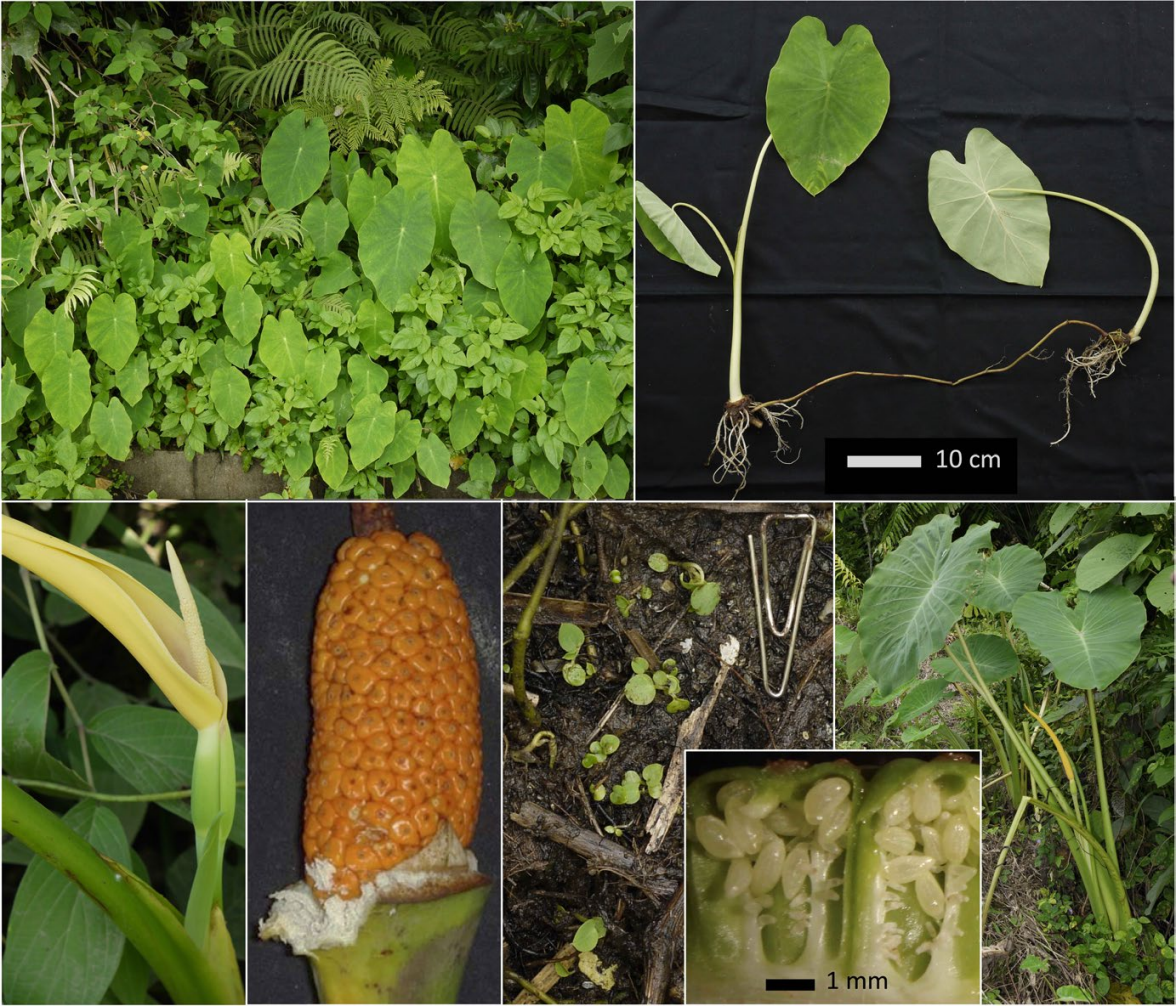
Habit and life cycle of wild taro (*Colocasia esculenta* var. *aquatilis*). Upper panel: commensal wild taro in clump (left), spreading by long stolons (right), along roadside (vegetative population, Okinawa, Japan). Lower panel: inflorescence with upper spathe open at anthesis (to release pollinating flies held in the lower chamber with female flowers), and spadix emerging (Papua New Guinea) (left); mature fruiting head with numerous berries that are attractive for birds (Myanmar) (middle left); wild taro flowering at edge of forest (right), with nearby seedlings (middle right) growing on ground saturated with water from seepage at foot of a steep hill (Markham valley, PNG); and vertical section through two immature, green berries, showing parietal placentation of seeds and unfertilized ovules (at different location) (inset)

**FIGURE 2:**
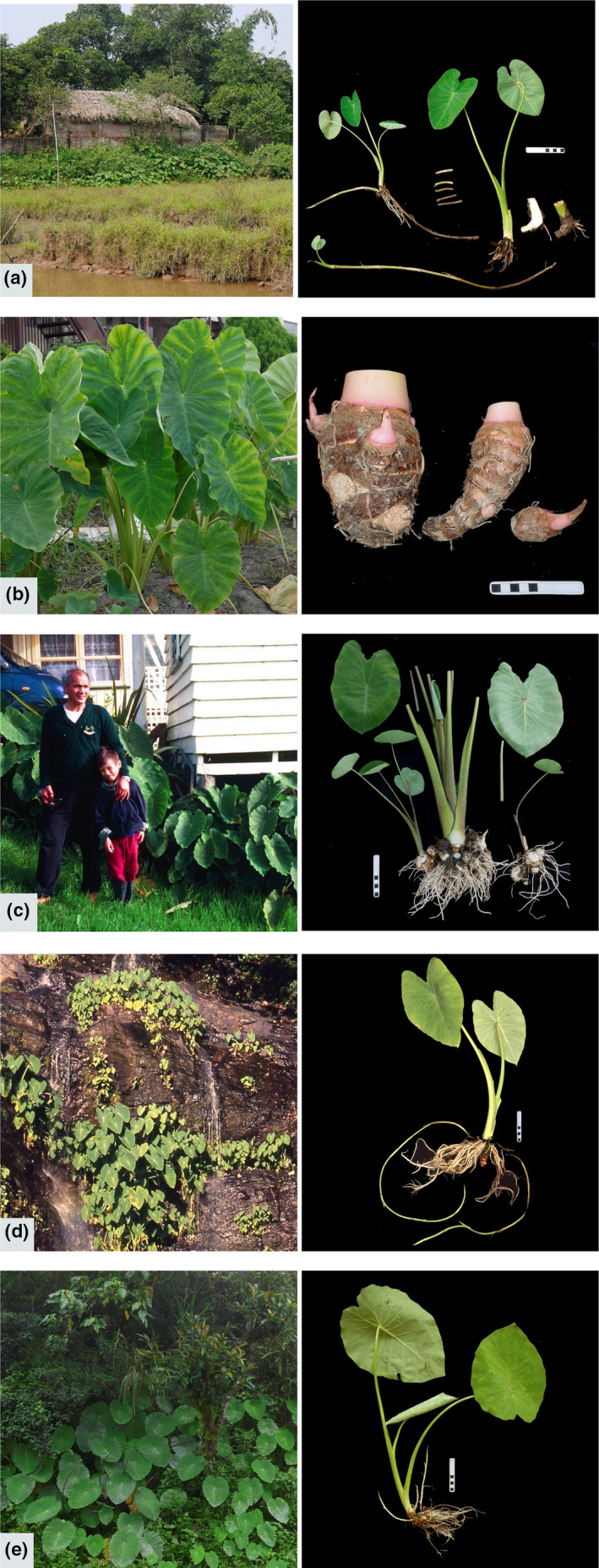
Example habitats and morphotypes (*Colocasia esculenta* and *Colocasia formosana*). Examples of wild and cultivated taros that display haplotypes in chloroplast Clades I–III. Left = habitat, right = morphology. Scale bar = 10 cm; (a, b) show specific plants tested; (c–e) show representative plants of cultivar or wild population tested. (a) Clade I, Type 1. *C. esculenta* var. *aquatilis* producing long stolons (commensal wild population at edge of wetland and settlement, northern Vietnam, sample CESVN05). Short stolon pieces in center illustrate a preparation step for eating as a wild vegetable. (b) Clade I, Type 1. *C. esculenta* var. *esculenta* producing large central corm (cultivar ex Cairo market, Egypt, sample CESJP02, in test cultivation). (c) Clade II. *C. esculenta* var. *antiquorum* producing abundant side‐corms (two cultivars, left, cv RR in house garden with house residents, Northland, New Zealand; right, cv *Ishikawa‐wase*, Kyoto, Japan; both are known triploids). (d) Clade III. *C. esculenta* var. *aquatilis* producing long stolons (wild population in rainforest, Queensland, Australia: left, at Isabella Falls; right, plant from bank of Russell River). (e) Clade III. *C. formosana* with young stolons (wild population in rainforest, vic. Banaue, Ifugao, Philippines)

Morphologically, *C. esculenta* (L.) Schott is a highly plastic species. A common wild morphotype, var. *aquatilis*, is found in wild, vegetative, and breeding populations from Southeast Asia to India, China, southern Japan, northern Australia, and Melanesia (Matthews, 1991, 2014) and produces relatively small mother corms, and vigorous long stolons instead of side‐corms (Figures 1 and 2). Two commonly cultivated morphotypes (Plucknett, 1983) are *C. esculenta* var. *antiquorum*, with many starchy side‐corms (and relatively small mother corms), and var. *esculenta* with large mother corms (and few side‐corms, or with stolons instead) (Figure 2) Cultivars vary greatly in their specific morphological, agronomic, and culinary traits (e.g., vegetative side‐shoot morphology, blade and petiole color, floral morphology, day‐length response, maturing time, and acridity of the different plant parts). Hotta (1970) recognized only two botanical varieties, var. *esculenta* and var. *aquatilis*, and assigned cultivars to “cultivar groups” within var. *esculenta*. The common morphotypes are difficult to recognize as formal botanical varieties (Hay, 1996; Plucknett, 1983), exist alongside many intermediate forms, and do not suggest an obvious domestication sequence.

Cytological surveys of cultivated taros established that diploid (2*n* = 2*x* = 28) and triploid (2*n* = 3*x* = 42) taros are common in Asia, that cultivars in Remote Oceania are all diploid (apart from modern introductions), and that triploid cultivars predominate at higher altitudes and latitudes in mainland Asia (India to China and Japan) (Kuruvilla & Singh, 1981; Matsuda & Nawata, 2002; Yen & Wheeler, 1968; Zhang & Zhang, 1990, 2000). Numerous surveys of cultivars in Asia and the Pacific have employed a range of tests for isoenzyme and DNA diversity (Devi, 2012; Helmkampf et al., 2017; Ivancic & Lebot, 2000; Matsuda & Nawata, 2002; Miyasaka et al., 2019; Zhu et al., 2000). Most effort has been focused on characterizing cultivated clones (cultivars), comparing cultivars in different geographical regions, reducing duplication in cultivar collections, and identifying nuclear genome markers of potential value for linkage mapping and plant breeding. Some surveys have included small numbers of “wild” or “wildtype” samples, without describing the wild plants or populations analyzed. Kreike et al. (2004) found high genetic diversity in 16 wild taros collected in Thailand, but gave no further information about the plants. Lakhanpaul et al. (2003) and Velayudhan (2008) surveyed and described wild and cultivated taros in southern to northern India. They noted possibilities for domestication in India, but found it difficult to distinguish progenitors and descendants among wild plants found to be closely related to cultivars. Chaïr et al. (2016) conducted a worldwide survey of genetic diversity in cultivated taros, using simple sequence repeat (SSR) analysis of alleles at 11 loci. Their results did not support primary domestication in New Guinea, suggesting instead dual domestication in India, or in Indian and Asian‐Pacific (Indo‐Malayan) genepools, with admixture between each genepool and the possibility of secondary domestication following introduction (with a genetic bottleneck) to New Guinea.

Yoshino (1975) described wild plants and populations in Nepal, then compared wild and cultivated plants from Nepal, Japan, China, and other countries using a variety of methods (Ochiai et al., 2001; Yoshino, 2002). In the first study of chloroplast DNA (cpDNA) variation in taro, Ochiai et al. (2000) used restriction fragment length polymoprhisms (RFLPs) to construct a phylogenetic tree for 41 accessions of wild and cultivated, diploid and triploid taros from Nepal, China, Japan, and Southeast Asia, with *Xanthosoma* sp., *Alocasia* spp. and *C. gigantea* (syn. *Leucocasia gigantea*) as outgroups. Taro formed a monophyletic group (Figure S4), but clade structure within taro was not discussed. Following isoenzyme analysis, Yoshino (2002) suggested that triploid cultivars in Nepal and Yunnan may have originated from separate diploid progenitors in each area. He considered the Himalayan south slope to be the likely area of origin for *C. esculenta* as a species, but did not comment on possible natural range limits. More recently, DNA sequences at four chloroplast loci were analyzed in *Alocasia*, a species‐rich genus in the same tribe as taro (Colocasieae) (Nauheimer et al., 2012), and in all 117 genera of Araceae (Nauheimer et al., 2012). *Colocasia*, *Remusatia*, and *Steudnera* grouped together and served as a near‐outgroup cluster for analyzing relationships among *Alocasia* species. Estimates for evolutionary divergence times were calculated for *Alocasia* and the outgroup taxa, using fossil evidence for calibration of a molecular clock model. This provided an initial estimate used to calibrate the phylogenetic model presented here.

In summary, previous studies of diversity in wild and cultivated taros did not identify wild source populations for primary domestication, but suggested the possibility of multiple areas of domestication, in India, China and Southeast Asia. Studies of phylogeny within *Colocasia* and other Araceae did not include closely related wild species within *Colocasia*. Our aim was to clarify the evolutionary history of taro through further study of chloroplast genomes in taro and closely related species, and to compare wild and cultivated taros in order to learn more about the evolutionary and geographical origins of cultivars.

To search for the geographical origins of any crop, the search space can be constrained by an estimate (explicit or implied) of the natural range of the species undergoing domestication. Our estimate of the maximum natural range of *C. esculenta* includes the region from India, China and mainland Southeast Asia to northern Australia and Melanesia, with geographical limits defined by the barriers of ocean (Indian and Pacific oceans) and climate (dry steppe and desert climates in northwestern India and central Australia; perpetually cold high altitudes of the Himalayan mountains; and temperate climates with cold winters at high latitudes in East Asia) (Matthews, 1991, 2014). Within these limits, wild breeding populations of taro are widely distributed, and have been seen by the present authors in southern and northern India, Bangladesh, Myanmar, Thailand, Vietnam, southern China, Taiwan, Philippines, Papua New Guinea, and northern Australia. Wild taro populations in northern Australia are confined to wet habitats in regions supplied with high rainfall by the southern monsoon, and wild populations in India and mainland Southeast Asia also appear to follow an approximate boundary defined by the northern monsoon (Matthews, 1991, 2013, 2014). In Australia, a survey of RFLPs in nuclear (NOR‐locus) rDNA revealed distinct wild taro populations in the Kimberley, Arnhemland, and northeast Queensland regions of northern Australia, raising the possibility of more than one pathway of introduction, including a possible combination of natural dispersal and human introduction (Matthews, 2014; Matthews & Terauchi, 1994). The possibility of multiple dispersals of taro into northern Australia was also suggested by the presence of two species of *Tarophagus* (*T. persephone* and *T. colocasiae*), the taro plant hopper (Matthews, 2003). Although the rDNA haplotype of the wild population in northeast Queensland was uniform over a distance of approximately 400 km, simple sequence repeat (SSR) diversity provided genetic confirmation of breeding at a Queensland location where fruits, seeds, and a specialist insect pollinator (*Colocasiomiya* sp.) were also observed (Hunt et al., 2013). The presence of specialist aroid pollinators (*Colocasiomiya*), and effective breeding by wild taros (with production of mature fruit and seeds), also distinguish wild populations inside the possible natural range from naturalized, wild populations that depend on vegetative propagation and dispersal outside the natural range (Matthews, 1995, 2014; Matthews, Takei, et al., 1992; Matthews et al., 2017). At Lake Euramoo in northeast Queensland, pollen records showed a rapid shift from sclerophyll to rainforest dominance at around 8,700 cal yr BP, with *Colocasia* pollen appearing in the period 8,700 to 5,000 BP (Haberle, 2005), suggesting natural expansion of the wild taro population with expansion of the rainforest.

Previously, we identified polymorphic regions in chloroplast DNA sequences from *C. esculenta* (Ahmed et al., 2012), and loci suitable for high‐resolution phylogeographic studies of *C. esculenta* and closely related taxa (Ahmed et al., 2013a). For the present study, we examined samples from across Asia and the Pacific, including wild and cultivated taros, other *Colocasia* species, and the closely related genera *Remusatia* and *Steudnera*. After combining data from six phylogenetically informative chloroplast loci, three distinct clades (CI–III) were found in *C. esculenta*: CI in cultivars and wild taros, CII in cultivars only—including cultivars introduced into commensal wild habitats in New Zealand to create wild food and fodder sources, for example, “var. RR” in Matthews (1985, 2014) (Figure 2c; Table S1)—and CIII in wild taros only. The apparently natural wild population in northeast Queensland, Australia, and phenotypically similar wild plants in Papua New Guinea (Matthews, 1991, 2014) displayed CIII, and represent a regional population that is unlikely to have been a locus for primary domestication (since CIII was not found in cultivated taros anywhere). Chloroplast diversity appears especially narrow in tropical cultivated taros with CI haplotypes, and wild CI subclades were found in the vicinity of the Bay of Bengal, suggesting a natural origin of the CI lineage in this region. We cannot pinpoint natural wild source populations for the domesticated CI and CII lineages of taro, but can suggest where to look for them (assuming that they still exist). The process of defining natural range limits, and detecting possible source populations for past domestication within those limits is an iterative process (Matthews et al., 2017). Further field exploration, sampling, and genetic analysis are now needed to define not just the natural range of taro, but also the natural range of each evolutionary lineage within the species.

## 2 MATERIALS AND METHODS

Chloroplast diversity was examined in 205 samples of taro and other closely related taxa. All names, taxonomic authorities, samples, and collection details are recorded in Table S1. Samples were collected in the period 1963 to 2012, and many samples or their source populations were described in previous studies (Coates et al., 1988; Hunt et al., 2013; Matthews, 1991, 2014; Matthews, Matsushita, et al., 1992; Matthews & Naing, 2020, 2005, 1992, 1994; Matthews, Takei, et al., 1992; Matthews et al., 2012, 2015; Nguyen et al., 2016; Yen & Wheeler, 1968). Descriptions in Table S1 are based on field observations by the authors or other collectors, and are used to classify samples as: “wild”, from a natural or commensal wild habitat, and not recognized as a cultivar, or “cult.”, from a cultivated habitat, or recognized as a cultivar. “Commensal wild taro populations” are those found in modified habitats in close proximity to human settlements. Some may be derived from nearby natural wild habitats, and others from other commensal wild populations by deliberate transplantation without cultivation. Known cultivars may become commensal wild by deliberate transplantation into a ditch or stream, or through soil erosion and water flow carrying vegetative parts (often referred to as “escape”). Each kind of movement can be followed by self‐propagation and further naturalization. Some cultivars in our sample set were collected with no accompanying record of habitat. Example habitats and common morphotypes are shown in Figure 2.

Earlier DNA extracts were prepared in the period 1987–1990 from leaf tissue stored and ground in liquid nitrogen (Matthews, 2014); more recent extracts were prepared from fresh leaves, or from leaf tissue dried and stored with silica gel, using either DNeasy Plant Mini Kit (Qiagen) or a modified standard protocol (Ahmed et al., 2009). Primers for PCR amplification at six phylogenetically informative loci in the taro chloroplast genome were designed and tested (Ahmed et al., 2012, 2013a, 2013b), and Sanger sequencing was carried out by Massey Genome Services, Massey University. The six loci, identified by the primer pair used, were ACECP 005, 016, 018, 026, 035, and 039; the target sequences ranged in size from 139 to 589 bp (Table 1), and single‐nucleotide polymorphisms (SNPs) were mostly located in noncoding regions. Primers, PCR reaction mix, thermocycling steps, and sequencing conditions were reported by Ahmed et al. (2013a, 2013b). GenBank database accession numbers for 1,045 sequences used in the present study (Ahmed et al., 2013b; Ahmed et al., 2015–2016) are listed in Table S2. Some shorter sequences (<200 nucleotides) were not deposited in GenBank but were used in the final alignment of concatenated sequences from the six loci.

**TABLE 1:** Primer sequences, chloroplast loci, and GenBank numbers

No.	Primer pair	Sequence	Locus	GenBank numbers	Size
1	ACECP005	F: AAAATGGGGTTCCTAGTGGA R: ACTCGAACTCGAAGAAATGG	*rps16* intron–5′‐*rps16* CDS–IGS toward *trnQ*	KF284854–KF285047	548
2	ACECP016	F: TTTACAGTCCGTCCCCATTA R: CATCTCTCTTTCAAGGAGGC	*trnY*–IGS–*trnE*	KF285048–KF285088	139
3	ACECP018	F: AGAGAGATCTTGTTGATATTTGT R: TAGTCATGATTCAACGGGTC	IGS between *trnT* and *psbD*	KF284164–KF284369	254
4	ACECP026	F: ACTACGGTAGAGCGGTTTAT R: AAAGTCATCTCACGTTCACC	*rbcL*	KF284370–KF284574	402
5	ACECP035	F: TGGTTAGGTATTGGAGCAAC R: GTGGACATTCTACAGAAGCA	*petD*–IGS–*rpoA*	KF285089–KF285278	253
6	ACECP039	F: AGTTACTCCCTTTTCCACCA R: GTAATGTTGGGGTGAACCAA	IGS between *rpl22* and *rps19*–*rps19*–IGS–*rpl2*	KF284575–KF284775	589

Note

Forward and reverse primer sequences and locus information for the six chloroplast loci were analyzed. The ACECP prefix used in the name of each primer pair is an acronym for “Ahmed, *Colocasia esculenta*, chloroplast.” Last column indicates size (bp) in the final alignment of all sequences obtained for each locus (not the size of individual sequences). Individual GenBank accession numbers are listed in Table S2.

We aligned and edited sequences using Geneious Pro v. 6.5 software (Drummond et al., 2009), deleting indels of varying lengths in the alignments, as indels cannot be modeled in the GTR model of evolution. To identify chloroplast haplotypes, and for downstream sequence analyses, we used the Mesquite software (Maddison & Maddison, 2011) to concatenate individual alignments for six loci, which generated a 2,185‐nucleotide‐long concatenated alignment. Identical sequences in the concatenated alignment were grouped together into Types (Table S3) using SplitsTree4 (Bryant & Moulton, 2004). In total, 205 samples displayed 34 haplotypes, including 14 grouped (identified by Type numbers) and 20 unique haplotypes (identified by individual sample numbers). The final sequence alignment was deposited in Dryad (Matthews et al., 2020). This alignment was used for downstream analyses, including neighbor‐net, maximum‐likelihood, and Bayesian analyses as below.

To avoid imposing any particular branching structure, as an initial assessment, haplotype relationships were visualized as a neighbor‐net diagram (Huson & Bryant, 2006) constructed using the SplitsTree4 software (Bryant & Moulton, 2004) (Figure S1). To develop a phylogenetic model, we used the JModelTest v. 2.1.3 software (Darriba et al., 2012) and found that the best model of substitution was the GTR + I + Г model (Tavare, 1986). The concatenated alignment was then used to build an optimal maximum‐likelihood (ML) tree with the PhyML software (Guindon & Gascuel, 2003). To find the optimal tree, we searched the tree space using the SPR (subtree prune and regraft) algorithm (Swofford et al., 1996) implemented in PhyML as a heuristic. In addition, nonparametric bootstrap resampling (100 bootstrap runs) was used to evaluate convergence on tree shape under the chosen substitution model. The FigTree v. 1.4 software (http://tree.bio.ed.ac.uk/software/figtree/) and the TreeDyn 198.3 software (Chevenet et al., 2006) were used to draw, edit, and save trees, and the final ML tree with bootstrap values is shown in Figure S2.

**FIGURE 3:**
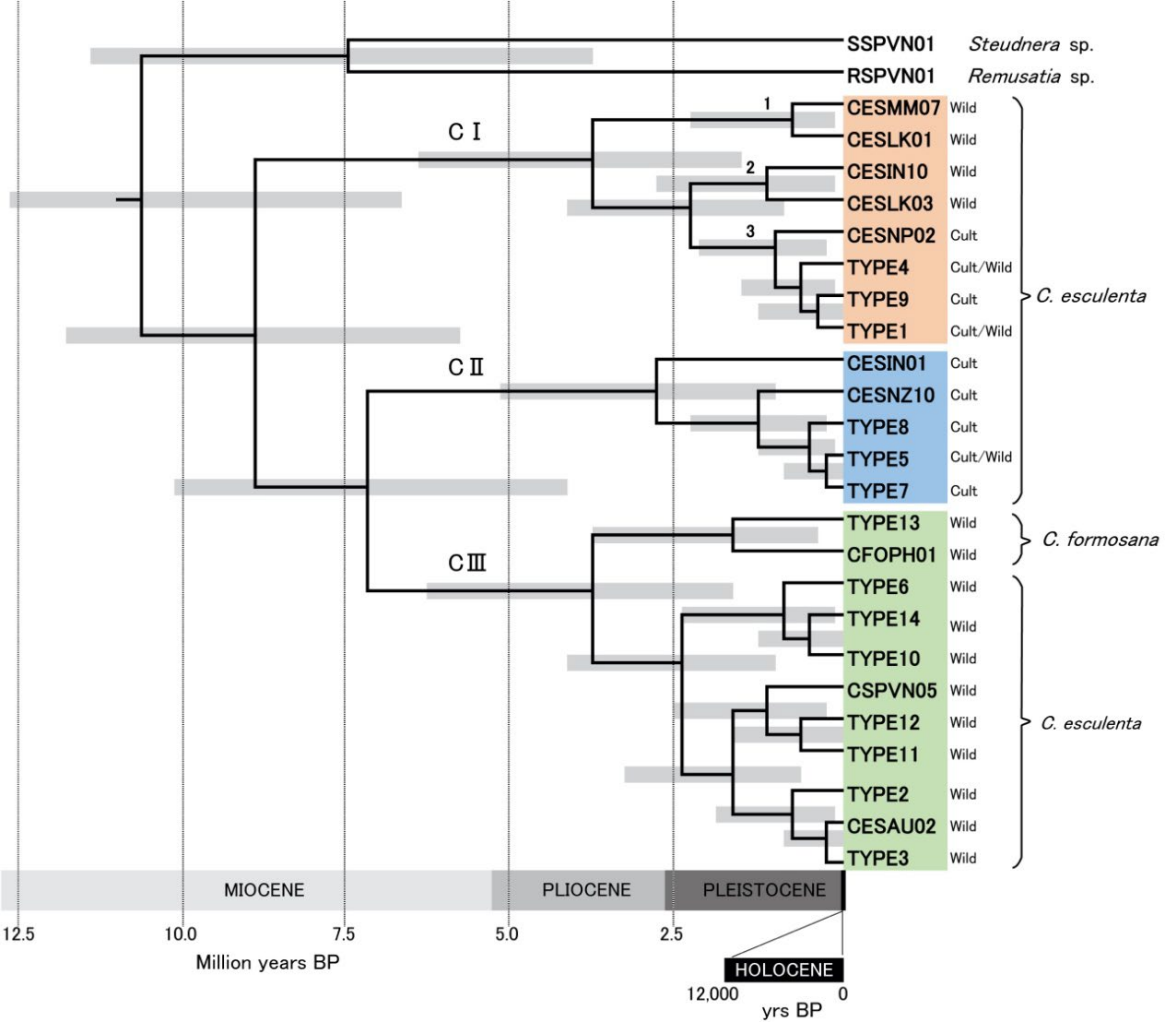
Approximate divergence times for chloroplast clades and subclades in *Colocasia esculenta* and *Colocasia formosana*. The gray horizontal bar at each node indicates 95% highest posterior distribution (HPD) probability range. Clade color code: orange = CI, blue = CII, green = CIII. Subclades 1–3 are discussed in the text. Sample habitats (wild or cultivated) associated with each clade and subclade are shown at right (see notes on habitats in Table S1). All Clade III samples were wild (*n* = 39, Figure 4). Terminal labels refer to unique haplotypes (identified by sample codes given in Table S1), or types (1–14) found in multiple samples (Tables S1 and S3)

To estimate evolutionary times of divergences within taro, we excluded all outgroup haplotypes except *Remusatia* and *Steudnera* (both in Tribe Colocasieae, together with *Colocasia*). (The CESNZ04 haplotype was also excluded due to its extreme long‐branch position in Figures S1 and S2, which was later traced to a data‐handling error). Our estimates of evolutionary divergence times are based on a secondary calibration of 10.84 Ma BP, the age previously estimated for the split between *C. esculenta* and *Remusatia/Steudnera* (Nauheimer, 2012). We then estimated divergence times of the taro clades using the Bayesian Evolutionary Analyses by Sampling Trees (BEAST v. 1.7.5) software (Drummond & Rambaut, 2007; Drummond et al., 2012). The analyses were carried out using BEAUTi v. 1.7.5 software to generate XML files for the BEAST input. We selected an uncorrelated log‐normal relaxed clock (Drummond et al., 2006) for divergence time estimation using the GTR + I + Г model of substitution and the coalescent constant model as tree priors. Five independent runs, each with a Markov‐Chain Monte Carlo chain length of 1,000,000 generations, were executed. Trees were sampled at every 1,000th step, giving 1,000 trees per run. The Tracer v. 1.5 software was used to evaluate the effective sample size in different runs. Trees from the five runs (5,000 total) were combined in the LogCombiner v. 1.7.5 software. A maximum clade credibility tree displaying median node heights (Figure 3) was inferred in the TreeAnnotator v. 1.7.5 software with a burn‐in limit of 500 (this removed the initial 10% of trees from each run, leaving 4,500 trees for calculation of the maximum clade credibility tree). The BEAUTi, Tracer, LogCombiner, and TreeAnnotator software are included in the BEAST package. Our estimate for the split between *Remusatia* and *Steudnera* (Figure 3) is 7.4 Ma BP, which is close to the previous estimate of 7.75 Ma BP (Nauheimer, 2012), thus confirming internal consistency in the BEAST analyses.

## 3 RESULTS

**FIGURE 4:**
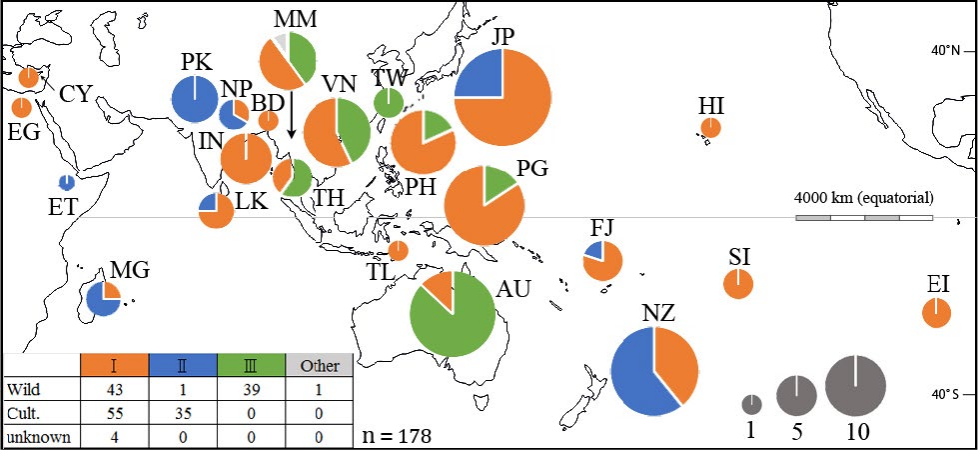
Map showing frequencies of taro chloroplast clades I–III in each area sampled. Sample number is indicated by pie chart diameter. The two‐letter code identifies each area according to country standard ISO 3166‐1, except for Hawaii (HI), Society Islands (SI), and Easter Island (EI). Clade III in TW and PH is represented by *Colocasia formosana*; all other samples belong to *Colocasia esculenta*, except possibly “Other” from Myanmar (recorded as *C. esculenta* in the field, based on vegetative characters, but showing an outgroup haplotype, CESMM12 (Figure 2, Figure S1). The number of wild or cultivated samples representing each clade is given at lower left (total *n* = 178)

The main results are summarized in Figures 3 and 4, and Tables S1 and S3. The initial neighbor‐net diagram (Figure S1) was not strictly tree‐like due to the presence of many contradictory internal splits, but four main clusters were obvious. The three clusters found in *C. esculenta* have been labeled Clades I, II, and III. The mutational dynamics of the most variable (noncoding) sequences in the chloroplast genome (Ahmed et al., 2012) make contradictory splits in the neighbor‐net analysis likely in population sample comparisons. One cluster comprised of outgroup taxa included a sample from Myanmar that was identified in the field as *C. esculenta* (CESMM12). This plant may be a hybrid or an undescribed species misidentified as *C. esculenta*. In the ML tree (Figure S2), *C. esculenta* appeared as a monophyletic group, with *Colocasia formosana* as a subclade within CIII, and the Myanmar sample CESMM12 again an outlier. The tree topology remained broadly similar to that shown in Figure S1.

The haplotypes of outgroup taxa clustered apart with the following exceptions (Tables S1 and S3). The CI, Type 1 haplotype was found in an introduced ornamental *C. affinis* collected in Luzon, Philippines, and CI, Type 4 in wild *Colocasia* sp. cf. *affinis* collected near Yangon, Myanmar. Other *Colocasia* species in northern Vietnam displayed CIII haplotypes: Type 11 and Type 12 in *C*. *lihengiae*, *C. menglaensis*, and *C. yunnanensis*, and the unique haplotype CSPVN05 in *Colocasia* sp. (The possibility of hybridization between *Colocasia* species is noted in Section 4).

Three distinct clades with deep divergence times were found in *C. esculenta*: CI in cultivars and wild taros, diploids (2*n* = 28) and triploids (2*n* = 42), distributed in tropical to subtropical regions; CII in cultivars, known triploids only, and temperate regions mainly; and CIII in wild taros, known diploids only, from mainland Southeast Asia to Australia and Papua New Guinea, in tropical to subtropical regions (Figure 3). Ploidy, as far as it is known from direct observation or inference, is recorded for individual samples in Table S1.

Within Clade I, the near‐identical Types 1, 4, and 9 in subclade 3 (Figure 3) were found in commensal wild populations producing long stolons (*C. esculenta* var. *aquatilis*, Figure 2a), and in cultivars with large mother corms (*C. esculenta* var. *esculenta*, Figure 2b). The vegetative population of *C. esculenta* var. *aquatilis* in Okinawa (Figure 1, upper panel) also displayed the CI, Type 1 haplotype (Table S1). The most common CI haplotype, Type 1 (Tables S1 and S3), was found in commensal wild taros (Asia and Pacific) and cultivars (Africa, Asia, and Pacific), explaining most of the observed range of CI (Figure 4).

Clade II haplotypes (Figures 3 and 4) were found in known triploid cultivars (Table S1) that produce abundant side‐corms in temperate regions (var. *antiquorum*, Figure 2c); none were found in known diploid cultivars or wild breeding populations. In our sample set, Clade II haplotypes were distributed in temperate to subtropical regions from Ethiopia and Madagascar, to Pakistan, Nepal, Japan, and New Zealand. One triploid sample with a Clade II haplotype was originally collected in Fiji in 1963, after introduction from India by immigrant farmers (see Coates et al., 1988; Yen & Wheeler, 1968) (CESIN01, no. 67, Table S1).

Clade III haplotypes (Figure 3) were found in known diploids (Table S1) or breeding wild populations of plants with long stolons (var. *aquatilis*, e.g., Figure 2d) in commensal or apparently natural wild habitats, in tropical to subtropical regions (Figure 4). *C. formosana* (Figure 2e), a wild, stolon‐bearing species of Taiwan and the Philippines (Matthews et al., 2012, 2015), also displayed CIII haplotypes that formed a distinct subclade (Figure 3, Table S1).

Evolutionary divergence times were estimated using Bayesian analyses, with the mean time of split of *C. esculenta* from *Remusatia* and *Steudnera* calibrated at 10.84 ± 1 Ma (see Section 2). The divergence time estimates for CI–CIII range from early to mid‐Miocene (approx. 7–8.5 million years ago), while most subclades and crown groups diverged from middle Pliocene to late Pleistocene (Figure 3). This phylogenetic model and correspondences between clade, habitat (wild or cultivated, Figure 3) and morphology within *C. esculenta* (Figure 2) indicate that: (a) *C. esculenta* is monophyletic as a species (containing clades CI, CII, and CIII), (b) within *C. esculenta*, cultivated taro is polyphyletic (CI, CII), and (c) within *C. esculenta*, wild taro with long stolons (var. *aquatilis*) is polyphyletic (CI, CIII). The implications and uncertainties of this model are discussed next.

## 4 DISCUSSION

The natural origin of *C. esculenta* as a species is believed to be in Southeast Asia, where all other wild *Colocasia* species are found (Matthews, 1991, 2014; Yoshino, 2002). Although samples are not shared between the two studies, our results broadly confirm the clade structure indicated by RFLP analysis of the chloroplast genome (Ochiai et al., 2000) (Figure S4). Chloroplast clades CI–III presumably originated in Southeast Asia early in the evolution of *C. esculenta* or genus *Colocasia*, during the late Miocene to Pliocene (Figure 3). With these estimates, we reject previous speculation that *C. esculenta* or *Colocasia* originated much earlier in Gondwanaland (Ivancic & Lebot, 1999; Lebot, 1999; Matthews, 2014). We suggest that wild CIII taros in the neighboring lowland rainforest zones of Australia and Papua New Guinea represent a Sunda floristic element that arrived following the mid‐Miocene collision with Sahul and emergence of a linking chain of islands, during the late Miocene to late Pliocene (Heaney, 1991; Whitmore, 1981). Birds, attracted by the fruit of taro (Caillon et al., 2006; Matthews & Naing, 2005), may have carried seeds between the wet habitats needed for seed germination and seedling survival (Hunt et al., 2013), across the Sunda shelf and islands leading to Sahul. This interpretation is consistent with the estimated late Miocene divergence of CIII (Figure 3), and the general view that the lowland forests of New Guinea are mostly derived from the Sunda lowland flora (Kooyman et al., 2019). There is also an initial indication of correlation between geography and subclade structure within CIII (Figure S3). With more comprehensive geographical sampling, nested clade phylogeographical analysis (NCPA) (Templeton, 2004) could be used to formally test the null hypothesis that nested clades of CIII haplotypes show no geographical associations across Sunda and Sahul.

The presence of Clade III haplotypes in other *Colocasia* species that have not been widely sampled adds uncertainty to our phylogenetic model. *C. formosana* Hayata displays a distinct subclade within Clade III, but the species has not been accepted as separate from *C. esculenta* by all taxonomists, and may be a northern, subtropical ecotype of *C. esculenta* (Matthews et al., 2015). Initial results (not presented) suggest the possibility of hybridization between wild taro and other *Colocasia* species that are sympatric with taro in northern Vietnam, which might explain the presence of Clade III haplotypes in *C. lihengiae*, *C. menglaensis* and *C. yunnanensis* (Table S1). *C. lihengiae* Long & Liu has recently been synonymized with *C. mannii* Hook. f., a wild species in Assam, northeast India, together with a report of *C. mannii* used as an edible leaf vegetable (Gogoi et al., 2019). The possibility of hybridization between this wild species and *C. esculenta* is of particular interest. The main pollinators of taro and closely related aroids (*Colocasiomiya* spp.) have quite specific host‐plant preferences (Sultana et al., 2006), but can cross‐pollinate closely related hosts (Miyake & Yafuso, 2005), and may be responsible for known or inferred instances of interspecific and intergeneric hybridization involving *Colocasia* spp., *Alocasia* spp. and other aroids (Matthews, 2014; Nauheimer, et al., 2012; Ochiai et al., 2000, 2001; Yoshino, 2002). Many ornamental aroid species have been found to hybridize quite readily when artificially pollinated (Henny, 1988; Snijder et al., 2007). Yoshino (2002) suggested that triploid taros may arise relatively frequently in wide crosses between taro and other taxa, and may be informative with regard to reproductive boundaries and speciation in *Colocasia* and closely related genera.

Wider taxonomic sampling and experimental breeding studies are needed to investigate interspecies hybridization, chloroplast transmission, and evolutionary relationships among all the known *Colocasia* species. The phylogenetic model presented here (Figure 3) may represent the maternal evolutionary history of taro, but this remains to be confirmed. Maternal transmission of chloroplast genomes is the dominant mode of transmission in most plants, but has not been studied experimentally in *Colocasia* species. Maternal, biparental and paternal transmission of chloroplasts has been demonstrated in numerous experimental crosses between different species of *Zantedeschia* (Araceae), but maternal transmission is dominant (Snijder et al., 2007). This example raises the likelihood that biparental and paternal transmission can occur in other aroids, including *Colocasia* species, at least occasionally.

All cultivated Clade I taros clustered together within subclade 3 as Types 1, 4, 9, which are distinguished by very few mutations in the chloroplast loci analyzed. No reliance can be placed on the timing of divergences between these three haplotypes (Figure 3), but subclade 3 as a whole is likely to have evolved somewhere in the vicinity of the two wild subclades 1 and 2, around of the Bay Bengal (i.e., in the general region of India, Sri Lanka, Bangladesh, and Myanmar). If the CI, Type I haplotype did not originate in plants under cultivation, it may also have evolved in this region, as part of subclade 3. The CI, Type 1 haplotype has the widest distribution under cultivation (Asia, Africa and Pacific; Table S1), and largely explains the wide distribution of Clade I in Figure 4. This haplotype appears to be very widespread among commensal wild taros used as food and as fodder in household pig husbandry (mainland and island Southeast Asia to Okinawa in southern Japan) (Figure 1, Okinawa; Figure 2a, Vietnam) (cf. Matthews & Naing, 2005; Matthews, Takei, et al., 1992; Matthews et al., 2012; Nguyen et al., 2015). In Southeast Asia, commensal wild taros form breeding populations with insect pollinators, and may be derived from (a) cultivars through naturalization (after escape from cultivation, or after deliberate transplanting into wild habitats), or from (b) natural wild populations within the unknown natural range of Clade I, subclade 3. The very wide distribution of CI, Type 1 cultivars indicates that they were highly favored after CI domestication at an unknown date, and commensal wild taro populations with this haplotype may have been favored sources for transplantation, with or without subsequent cultivation. These possibilities will be considered further as we interpret the results from New Guinea and Australia.

Three kinds of circumstantial evidence supported the hypothesis of taro domestication in New Guinea from around 10,000 years ago: earthworks indicating water control at Kuk swamp archaeological site, plant remains attributed to taro at Kuk, and the presence of apparently natural wild taro populations in New Guinea (Fullagar et al., 2006; Golson, 1989; Golson et al., 2017; Matthews, 1991). Our genetic data do not support the New Guinea domestication hypothesis: all New Guinean cultivars displayed the CI, Type 1 haplotype, and not the CIII haplotypes found in mainland New Guinea, East New Britain, and northeast Australia (Figures 2d and 3, Table S1). This does not discredit or contradict archaeological evidence for early use and cultivation of taro in Melanesia. Use of stone flakes to process taro at about 28,000 years BP, in the Solomon Islands (Loy et al., 1992), might reflect early use of CIII wild taros before CI, Type 1 cultivars arrived from Southeast Asia. If CI, Type I was cultivated at Kuk from 10,000 years BP (at earliest), the crop may have been introduced after late Pleistocene domestication in Southeast Asia. Alternatively, if CIII wild taros were utilized or taken into cultivation during the early phases at Kuk, they may have been replaced by Type 1 cultivars after an early‐ or mid‐Holocene domestication in Southeast Asia. If the Type 1 cultivars were first introduced by Austronesian speakers entering Melanesia from Southeast Asia 4000–3,000 years BP (Gaffney et al., 2015; Spriggs, 2011), they might represent an Austronesian contribution to the proposed “Colocasian revolution” during Phase 4 at Kuk, around 1,200 years BP (Bayliss‐Smith & Golson, 1992). More recent introductions and replacements of cultivars are also possible, as movements of taro cultivars in Asia and the Pacific are likely to have been continuous over time.

Genetic diversity was previously reported among wild taros in northern Australia (see Introduction). In the present survey, we found the CI, Type I haplotype in one plant collected in the remote Kimberley region (CESAU04, Table S1; Scarlett, 1985), and also among the wild plants collected in New Guinea. It is theoretically possible that wild CI plants are naturally present in New Guinea, and this can also be suggested for northern Australia, but all the CI plants found show the specific Type I haplotype common in cultivated taros from Africa to Asia and Remote Oceania, and in commensal wild taros of Southeast Asia and southern Japan (Table S1). Throughout the known range of CI, Type 1, commensal wild populations with this haplotype are likely to be derived from cultivated plants, or from commensal wild plants transported as useful plants and introduced into wild habitats. The Clade I, Type 1 taro in Kimberley could represent a prehistoric introduction from island Southeast Asia or New Guinea, by early agriculturalists, hunter‐gatherers or sea‐faring traders, along with other early plant and animal introductions (Denham, Donohue, et al., 2009; Fillios & Taçon, 2016). That this specific haplotype was first domesticated in New Guinea, rather than any other area where it is found wild, is possible but improbable. It is also unlikely that this specific haplotype dispersed naturally through Southeast Asia to Australia and New Guinea, without any differentiation as seen in Clade III.

Primary domestication of CI and CII cultigens may have taken place in multiple regions and environments suggested in previous studies (Chaïr et al., 2016; Matthews, 2014; Yoshino, 2002), and multiple wild and cultivated genepools are likely to be involved in secondary domestication or improvement of the crop. New Guinea, with its rich archaeological evidence for early landscape modification and wetland cultivation is certainly of great significance for discussing the possible trajectories of taro domestication and dispersal, but is not the only candidate region for primary domestication of the crop, as suggested diagrammatically in some secondary literature (e.g., Fuller et al., 2014; Larson et al., 2014). The original archaeological literature concerning taro in New Guinea has never rejected the possibility of primary domestication outside New Guinea, while gradually developing a range of possible explanations for forest clearance, wetland drainage, archaeobotanical evidence for taro and other useful plants, and economic shifts from mainly hunting‐and‐gathering toward greater dependence on agricultural production (Denham, Fullagar, et al., 2009; Golson et al., 2017).

Efforts are now needed to define the natural range limits (Matthews, 2014; Matthews et al., 2017) of Clades I, II and III in Sunda and Sahul—from Himalaya to southern China, southern India, and island Southeast Asia, to northern Australia, Papua New Guinea, and eastern Melanesia. The great diversity reported in cultivated taros (in surveys of phenotypes, isoenzymes, and nuclear DNA) may partly reflect hybridization between different evolutionary lineages of wild and cultivated taros (CI, CII, and CIII), and between *C. esculenta* and other *Colocasia* species. Taro is a clonally propagated crop, but swidden systems with long fallows are likely to have created abundant opportunities for cycles of flowering, breeding, and farmer selection (Matthews, 2014). Such cycles may have led to crossing between diploid cultivars, introgression between different evolutionary lineages, and interspecific hybridization in regions of sympatry, generating diversity in vegetative and floral morphology, acridity, culinary qualities, and other characters.

In India, stolon‐bearing wild taro populations have been reported in most regions (in southern, eastern, and northern India, from latitudes 8° 85′ to 35° 0′ N; in tropical evergreen forests at low elevations to moist or marshy upland locations) and represent candidate source populations for domestication (Velayudhan, 2008). Lakhanpaul et al. (2003) analyzed randomly amplified polymorphic DNAs (RAPDs) from wild and cultivated morphotypes from throughout India, and found two main groups (unlabeled in their UPGMA tree diagram) containing mainly var. *esculenta* (clusters I and II in the diagram) and mainly var. *antiquorum* (clusters II and IV), with numerous intermediate morphological forms in each main group. Through correspondence with the common morphotypes (var. *esculenta*, var. *antiquorum*), we can infer that chloroplast Clades I and II are present in India. Lakhanpaul et al. (2003) noted that wild forms in each cluster may be “direct descendants or variants” of the progenitors of cultivars, or derived from cultivars through “chance escape” into the wild. Although no direct comparisons can be made with our study, the Indian survey set may be largely composed of CI and CII cultivars and numerous hybrids between them (the intermediate morphological forms noted above). If so, then CII diploids may exist in India (and also Nepal, China, and Thailand, see Figure S4), and may have hybridized with CI diploids. Hybridization between two evolutionary lineages in India (with CI and CII chloroplast genomes) may also partly explain the admixtures seen by Chaïr et al. (2016) in their survey of genetic diversity in cultivated taro.

What kinds of selection were involved in the primary domestication of taro? Here we consider morphological and biochemical traits. Long stolons (with indeterminate growth) are a trait shared by other *Colocasia* species that occupy wet or damp habitats (e.g., *C. affinis*, *C. fallax*, *C. formosana*, *C. lihengiae*, *C. menglaensis*, *C. yunnanensis*) (authors' observations, and taxonomic reports). Stolons and side‐corms represent mutually exclusive developmental directions for buds located in leaf axils on the mother corm, but both kinds of side‐shoot can be favored for consumption and clonal propagation by humans. If stolon production is a basal trait in *C. esculenta*, then starchy side‐corms in modern CI and CII cultivars may reflect human selection in one or both lineages, followed by introgression between them. Alternatively, if side‐corms in CII triploids (var. *antiquorum*) are a natural, evolutionary adaptation to dry and cool upland conditions in Himalaya, then wild CII diploids that produce side‐corms might exist, and stolon‐bearing CI wild taros may have been transformed by introgression from diploid CII domesticates.

Primary domestication “episodes” (Fuller et al., 2014) for wild Clade I and/or Clade II taros did not necessarily involve selection for traits related to vegetative propagation and production. Edibility may have been the first concern of early users of wild taro populations, leading to selection for reduced acridity in the plant, or for a greater sensitivity of acridity to heating and other methods of food preparation (Matthews, 2010). Changes in acridity might have continued to spread through improving selection in diverse lineages of cultivated taro. The properties of acridity in wild taro populations have rarely been studied. Velayudhan (2008) reported diversity in the acridity of corms and leaves of wild taros using subjective taste trials. Such trials are also used in modern taro breeding programs, as there is no easy method for objective, quantitative measurement of this subjectively unpleasant trait (Bradbury & Nixon, 1998; Konno et al., 2014; Matthews, 2004, 2010). Acridity has value to farmers as a natural protection for the crop against herbivory, while consumers favor cultivars for which the effect can be eliminated, so balancing selection may have prevented the complete loss of acridity in cultivars. Wild populations of *C. formosana* in Taiwan and the Philippines, and of *C. esculenta* in northern Queensland, are known to be very acrid and difficult to prepare for eating (Matthews, 2014; Matthews et al., 2012, 2015). The complete absence of CIII cultivars in our survey (Figures 3 and 4) may mean that selection for reduced acridity has never been effective in CIII populations (and conversely, that such selection was effective in CI and CII populations that gave rise to cultivars). In taro breeding programs, acridity is a key issue for cultivar acceptability. Strong acridity may have restricted the ability of CIII wild taros to contribute (through introgression) to diversification and improvement of the crop, after acridity was reduced in early CI and CII cultivars. In areas where CIII wild taros are absent, farmers may have experimented more freely with unfamiliar new plants (seedlings) that appeared in or around their gardens. This might partly explain, for example, the large number of cultivars found in Hawaii (Helmkampf et al., 2017), far outside the natural range of taro.

Evolutionary adaptations at higher to lower altitudes in Himalaya and Southeast Asia sensu lato (or monsoonal Asia including parts of India and China) may have facilitated the early and continuing spread of CI and CII cultivars in tropical and temperate latitudes respectively. This and the diversity of wild populations over a wide geographical range promise well for the ability of *C. esculenta* to acquire new traits under further natural and human selection and to accommodate future climate change as a species and crop. In contrast, numerous wild *Colocasia* species occupy restricted montane habitats in Southeast Asia and face the double threat of habitat loss through deforestation and rapid climate change—both of which must affect not just the plants but also their insect pollinators.

## CONFLICT OF INTEREST

None of the authors have competing interests to declare.

## AUTHOR CONTRIBUTIONS

**Ibrar Ahmed:** Conceptualization (equal); Data curation (lead); Formal analysis (lead); Funding acquisition (equal); Investigation (equal); Methodology (equal); Project administration (equal); Resources (supporting); Visualization (equal); Writing‐original draft (equal); Writing‐review & editing (equal). **Peter J. Lockhart:** Conceptualization (supporting); Formal analysis (supporting); Funding acquisition (equal); Methodology (equal); Project administration (equal); Resources (equal); Supervision (lead); Writing‐original draft (supporting); Writing‐review & editing (supporting). **Esperanza M. G. Agoo:** Investigation (supporting); Methodology (supporting); Resources (supporting); Writing‐original draft (supporting). **Kyaw W. Naing:** Investigation (supporting); Methodology (supporting); Resources (supporting); Writing‐original draft (supporting). **Dzu V. Nguyen:** Funding acquisition (supporting); Investigation (supporting); Methodology (supporting); Resources (supporting); Writing‐original draft (supporting). **Dilip K. Medhi:** Investigation (supporting); Methodology (supporting); Resources (supporting); Writing‐original draft (supporting). **Peter J. Matthews:** Conceptualization (equal); Data curation (supporting); Formal analysis (supporting); Funding acquisition (equal); Investigation (equal); Methodology (equal); Project administration (equal); Resources (equal); Supervision (supporting); Visualization (equal); Writing‐original draft (equal); Writing‐review & editing (equal).

### Open Research Badges

This article has earned an Open Data Badge for making publicly available the digitally‐shareable data necessary to reproduce the reported results. The data is available at (a) GENBANK: www.ncbi.nlm.nih.gov/genbank/, (b) DRYAD: https://doi.org/10.5061/dryad.nvx0k6dpj.

## Supporting information

Fig S1‐S4

Table S1‐S3

## Data Availability

All data are available in the article, supplementary materials, and in the GenBank and Dryad open‐access archives (Ahmed et al., 2013b; Ahmed et al., 2015–2016; Matthews et al., 2020). Dryad https://doi.org/10.5061/dryad.nvx0k6dpj

## References

[ece36958-bib-0001] Ahmed, I. , Biggs, P. J. , Matthews, P. J. , Collins, L. J. , Hendy, M. D. , & Lockhart, P. J. (2012). Mutational dynamics of aroid chloroplast genomes. Genome Biology and Evolution, 4, 1316–1323. 23204304 10.1093/gbe/evs110 PMC3542561

[ece36958-bib-0002] Ahmed, I. , Islam, M. , Arshad, W. , Mannan, A. , & Mirza, B. (2009). High‐quality plant DNA extraction for PCR: An easy approach. Journal of Applied Genetics, 50, 105–107. 19433907 10.1007/BF03195661

[ece36958-bib-0003] Ahmed, I. , Matthews, P. J. , Biggs, P. J. , Naeem, M. , McLenachan, P. A. , & Lockhart, P. J. (2013a). Identification of chloroplast genome loci suitable for high‐resolution phylogeographic studies of *Colocasia esculenta* (L.) Schott (Araceae) and closely related taxa. Molecular Ecology Resources, 13, 929–937. 23718317 10.1111/1755-0998.12128

[ece36958-bib-0004] Ahmed, I. , Matthews, P. J. , Biggs, P. J. , Naeem, M. , McLenachan, P. A. , & Lockhart, P. J. (2013b). Nucleotide sequences (DNA), Genbank: JN105395–JN105632 Retrieved from www.ncbi.nlm.nih.gov/genbank/

[ece36958-bib-0005] Ahmed, I. , Matthews, P. J. , & Lockhart, P. J. (2015–2016). Nucleotide sequences (DNA), Genbank: KF284854–KF285047, KF285048–KF285088, KF284164–KF284369, KF284370–KF284574, KF285089–KF285278, KF284575–KF284775. Retrieved from www.ncbi.nlm.nih.gov/genbank/

[ece36958-bib-0006] Bayliss‐Smith, T. , & Golson, J. (1992). A Colocasian revolution in the New Guinea Highlands? Insights from phase 4 at Kuk. Archaeology in Oceania, 27, 1–21. 10.1002/j.1834-4453.1992.tb00279.x

[ece36958-bib-0007] Bradbury, J. H. , & Nixon, R. W. (1998). The acridity of raphides from the edible aroids. Journal of the Science of Food and Agriculture, 76, 608–616. 10.1002/(SICI)1097-0010(199804)76:4<608:AID-JSFA996>3.0.CO;2-2

[ece36958-bib-0008] Bryant, D. , & Moulton, V. (2004). Neighbor‐Net: An agglomerative method for the construction of phylogenetic networks. Molecular Biology and Evolution, 21, 255–265. 10.1093/molbev/msh018 14660700

[ece36958-bib-0009] Caillon, S. , Quero‐Garcia, J. , Lescure, J. P. , & Lebot, V. (2006). Nature of taro (*Colocasia esculenta* (L.) Schott) genetic diversity prevalent in a Pacific Ocean island, Vanua Lava, Vanuatu. Genetic Resources and Crop Evolution, 53, 1273–1289. 10.1007/s10722-005-3877-x

[ece36958-bib-0010] Chaïr, H. , Traore, R. E. , Duval, M. F. , Rivallan, R. , Mukherjee, A. , Aboagye, L. M. , Van Rensburg, W. J. , Andrianavalona, V. , Pinheiro de Carvalho, M. A. A. , Saborio, F. , Sri Prana, M. , Komolong, B. , Lawac, F. , & Lebot, V. (2016). Genetic diversification and dispersal of taro (*Colocasia esculenta* (L.) Schott). PLoS One, 11, e0157712. 27314588 10.1371/journal.pone.0157712 PMC4912093

[ece36958-bib-0011] Chevenet, F. , Brun, C. , Bañuls, A.‐L. , Jacq, B. , & Christen, R. (2006). TreeDyn: Towards dynamic graphics and annotations for analyses of trees. BMC Bioinformatics, 7, 439. 17032440 10.1186/1471-2105-7-439 PMC1615880

[ece36958-bib-0012] Coates, D. J. , Yen, D. E. , & Gaffey, P. M. (1988). Chromosome variation in taro, *Colocasia esculenta*: Implications for origin in the Pacific. Cytologia (Tokyo), 53, 551–560.

[ece36958-bib-0013] Darriba, D. , Taboada, G. L. , Doallo, R. , & Posada, D. (2012). jModelTest 2: More models, new heuristics and parallel computing. Nature Methods, 9, 772. 10.1038/nmeth.2109 PMC4594756 22847109

[ece36958-bib-0014] de Candolle, A. (1885). Origin of cultivated plants. D. Appleton & Company.

[ece36958-bib-0015] Denham, T. , Donohue, M. , & Booth, S. (2009). Horticultural experimentation in northern Australia reconsidered. Antiquity, 83, 634–648.

[ece36958-bib-0016] Denham, T. , Fullagar, R. , & Head, L. (2009). Plant exploitation on Sahul: From colonisation to the emergence of regional specialisation during the Holocene. Quarternary International, 202, 29–40.

[ece36958-bib-0017] Devi, A. A. (2012). Genetic diversity analysis in taro using molecular markers – An overview. Journal of Root Crops, 38, 15–25.

[ece36958-bib-0018] Drummond, A. J. , Ashton, B. , Cheung, M. , Heled, J. , Kearse, M. , Moir, R. , Stones‐Havas, S. , Thierer, T. , & Wilson, A. (2009). Geneious v4.7. Biomatters.

[ece36958-bib-0019] Drummond, A. J. , Ho, S. Y. W. , Phillips, M. J. , & Rambaut, A. (2006). Relaxed phylogenetics and dating with confidence. PLoS Biology, 4, e88. 16683862 10.1371/journal.pbio.0040088 PMC1395354

[ece36958-bib-0020] Drummond, A. J. , & Rambaut, A. (2007). BEAST: Bayesian evolutionary analysis by sampling trees. BMC Evolutionary Biology, 7, 214. 17996036 10.1186/1471-2148-7-214 PMC2247476

[ece36958-bib-0021] Drummond, A. J. , Suchard, M. A. , Xie, D. , & Rambaut, A. (2012). Bayesian phylogenetics with BEAUti and the BEAST 1.7. Molecular Biology and Evolution, 29, 1969–1973. 22367748 10.1093/molbev/mss075 PMC3408070

[ece36958-bib-0022] Fillios, M. A. , & Taçon, P. S. C. (2016). Who let the dogs in? A review of the recent genetic evidence for the introduction of the dingo to Australia and implications for the movement of people. Journal of Archaeological Science: Reports, 7, 782–792.

[ece36958-bib-0023] Fullagar, R. , Field, J. , Denham, T. , & Lentfer, C. (2006). Early and mid Holocene tool‐use and processing of taro (*Colocasia esculenta*), yam (*Dioscorea* sp.) and other plants at Kuk Swamp in the highlands of Papua New Guinea. Journal of Archaeological Science, 33, 595–614.

[ece36958-bib-0024] Fuller, D. Q. , Denham, T. , Arroyo‐Kalin, M. , Lucas, L. , Stevens, C. J. , Qin, L. , Allaby, R. G. , & Purugganan, M. D. (2014). Convergent evolution and parallelism in plant domestication revealed by an expanding archaeological record. Proceedings of the National Academy of Sciences USA, 111, 6147–6152. 10.1073/pnas.1308937110 PMC4035951 24753577

[ece36958-bib-0025] Gaffney, D. , Summerhayes, G. R. , Ford, A. , Scott, J. M. , Denham, T. , Field, J. , & Dickinson, W. R. (2015). Earliest pottery on New Guinea mainland reveals Austronesian influences in highland environments 3000 years ago. PLoS One, 10, e0134497. 26331310 10.1371/journal.pone.0134497 PMC4557931

[ece36958-bib-0026] Gogoi, R. , Borah, S. , & Sarma, J. (2019). Taxonomic identity and lectotypification of *Colocasia mannii* (Araceae), a little known species from Northeast India. Nelumbo, 61, 131–134.

[ece36958-bib-0027] Golson, J. (1989). The Origins and Development of New Guinea Agriculture In Harris D. R., & Hillman G. C. (Eds.), Foraging and farming: The evolution of plant exploitation (pp. 678–687). Unwin Hyman.

[ece36958-bib-0028] Golson, J. , Denham, T. , Hughes, P. , Swadling, P. , & Muke, J. (Eds.) (2017). Ten thousand years of cultivation at Kuk swamp in the highlands of Papua New Guinea. ANU Press.

[ece36958-bib-0029] Grimaldi, I. M. (2016). Taro across the oceans, journeys of one of our oldest crops In Thanheiser U. (Ed.), News from the past, progress in African archaeobotany. Proceedings of the 7th international workshop on African Archaeobotany in Vienna, 2–5 July 2012 (pp. 67–81). Barkhuis.

[ece36958-bib-0030] Grimaldi, I. M. , Muthukumaran, S. , Tozzi, G. , Nastasi, A. , Boivin, N. , Matthews, P. J. , & van Andel, T. (2018). Literary evidence for taro in the ancient Mediterranean: A chronology of names and uses in a multilingual world. PLoS One, 13(6), e0198333 10.1371/journal.pone.0198333 29870533 PMC5988270

[ece36958-bib-0031] Guindon, S. , & Gascuel, O. (2003). A simple, fast, and accurate algorithm to estimate large phylogenies by maximum likelihood. Systematic Biology, 52, 696–704. 14530136 10.1080/10635150390235520

[ece36958-bib-0032] Haberle, S. G. (2005). A 23,000‐yr pollen record from Lake Euramoo, wet tropics of NE Queensland, Australia. Quaternary Research, 64, 343–356.

[ece36958-bib-0033] Hay, A. (1996). A new Bornean species of *Colocasia* Schott (Araceae: Colocasieae) with a synopsis of the genus in Malesia and Australia. Sandakania, 7, 31–48.

[ece36958-bib-0034] Heaney, L. R. (1991). A synopsis of climate and vegetational change in Southeast Asia. Climatic Change, 19, 53–61.

[ece36958-bib-0035] Helmkampf, M. , Wolfgruber, T. K. , Bellinger, M. R. , Paudel, R. , Kantar, M. B. , Miyasaka, S. C. , Kimball, H. L. , Brown, A. , Veillet, A. , Read, A. , & Shintaku, M. (2017). Phylogenetic relationships, breeding implications, and cultivation history of Hawaiian taro (*Colocasia esculenta*) through genome‐wide SNP genotyping. Journal of Heredity, 109, e1–e11. 10.1093/jhered/esx070 PMC6018804 28992295

[ece36958-bib-0036] Henny, R. J. (1988). Ornamental aroids: Culture and breeding. Horticultural Reviews, 10, 1–33. 10.1002/9781118060834.ch1

[ece36958-bib-0037] Hotta, M. (1970). A system of the Family Araceae in Japan and adjacent areas, Part I. Memoirs of the Faculty of Science, Kyoto University Series of Biology, 4, 72–96.

[ece36958-bib-0038] Hunt, H. V. , Moots, H. M. , & Matthews, P. J. (2013). Genetic data confirms field evidence for natural breeding in a wild taro population (*Colocasia esculenta*) in northern Queensland, Australia. Genetic Resources and Crop Evolution, 60, 1695–1707. 10.1007/s10722-012-9952-1

[ece36958-bib-0039] Huson, D. H. , & Bryant, D. (2006). Application of phylogenetic networks in evolutionary studies. Molecular Biology and Evolution, 23, 254–267. 10.1093/molbev/msj030 16221896

[ece36958-bib-0040] Ivancic, A. , & Lebot, V. (1999). Botany and genetics of New Caledonian wild taro, *Colocasia esculenta* . Pacific Science, 53, 273–285.

[ece36958-bib-0041] Ivancic, A. , & Lebot, V. (2000). The genetics and breeding of taro. CIRAD.

[ece36958-bib-0042] Konno, K. , Inoue, T. A. , & Nakamura, M. (2014). Synergistic defensive function of raphides and protease through the needle effect. PLoS One, 9, e91341. 24621613 10.1371/journal.pone.0091341 PMC3951349

[ece36958-bib-0043] Kooyman, R. M. , Morley, R. J. , Crayn, D. , Joyce, E. M. , Rossetto, M. , Slik, J. W. F. , Strijk, J. S. , Su, T. , Yap, J.‐Y.‐S. , & Wilf, P. (2019). Origins and assembly of Malesian rainforests. Annual Review of Ecology, Evolution, and Systematics, 50, 119–143.

[ece36958-bib-0044] Kreike, C. M. , Eck, H. J. V. , & Lebot, V. (2004). Genetic diversity of taro, *Colocasia esculenta* (L.) Schott, in Southeast Asia and the Pacific. Theoretical and Applied Genetics, 109, 761–768. 15156282 10.1007/s00122-004-1691-z

[ece36958-bib-0045] Kuruvilla, K. M. , & Singh, A. (1981). Karyotypic and electrophoretic studies on taro and its origin. Euphytica, 30, 405–413.

[ece36958-bib-0046] Lakhanpaul, S. , Velayudhan, K. C. , & Bhat, K. V. (2003). Analysis of genetic diversity in Indian taro *Colocasia esculenta* (L.) Schott using random amplified polymorphic DNA (RAPD) markers. Genetic Resources and Crop Evolution, 50, 603–609.

[ece36958-bib-0047] Larson, G. , Piperno, D. R. , Allaby, R. G. , Purugganan, M. D. , Andersson, L. , Arroyo‐Kalin, M. , Barton, L. , Vigueira, C. C. , Denham, T. , Dobney, K. , Doust, A. N. , Gepts, P. , Gilbert, M. T. P. , Gremillion, K. J. , Lucas, L. , Lukens, L. , Marshall, F. B. , Olsen, K. M. , Pires, J. C. , … Fuller, D. Q. (2014). Current perspectives and the future of domestication studies. Proceedings of the National Academy of Sciences USA, 111, 6137–6146. 10.1073/pnas.1323964111 PMC4035915 24757054

[ece36958-bib-0048] Lebot, V. (1999). Biomolecular evidence for plant domestication in Sahul. Genetic Resources and Crop Evolution, 46, 619–628.

[ece36958-bib-0049] Loy, T. H. , Spriggs, M. , & Wickler, S. (1992). Direct evidence for human use of plants 28,000 years ago: Starch residues on stone artefacts from the northern Solomon Islands. Antiquity, 66, 898–912.

[ece36958-bib-0050] Maddison, W. P. , & Maddison, D. R. (2011). Mesquite: A modular system for evolutionary analysis. Version 2.75. Retrieved from www.mesquiteproject.org/

[ece36958-bib-0051] Matsuda, M. , & Nawata, E. (2002). Geographical distribution of ribosomal DNA variation in taro, *Colocasia esculenta* (L.) Schott, in eastern Asia. Euphytica, 128, 165–172.

[ece36958-bib-0052] Matthews, P. J. (1985). Nga taro o Aotearoa. Journal of the Polynesian Society, 94, 253–272.

[ece36958-bib-0053] Matthews, P. J. (1991). A possible tropical wildtype taro: *Colocasia esculenta* var. *aquatilis* . Indo‐Pacific Prehistory Association Bulletin, 11, 69–81.

[ece36958-bib-0054] Matthews, P. (1995). Aroids and the Austronesians. Tropics, 4, 105–126.

[ece36958-bib-0055] Matthews, P. J. (2003). Taro planthoppers (*Tarophagus* spp.) in Australia and the origins of taro (*Colocasia esculenta*) in Oceania. Archaeology in Oceania, 38, 192–202.

[ece36958-bib-0056] Matthews, P. J. (2004). Genetic diversity in taro, and the preservation of culinary knowledge. Ethnobotany Research and Applications, 2, 55–71.

[ece36958-bib-0057] Matthews, P. J. (2006). Written records of taro in the eastern Mediterranean In Ertug Z. F. (Ed.), Ethnobotany: At the junction of the continents and the disciplines (Proceedings of the IVth International Congress of Ethnobotany, ICEB, 21–26 August, Istanbul, Turkey) (pp. 419–426). Yayinlari.

[ece36958-bib-0058] Matthews, P. J. (2010). An introduction to the history of taro as a food In Rao V. R., Matthews P. J., Eyzaguirre P. B., & Hunter D. (Eds.), The global diversity of taro: Ethnobotany and conservation (pp. 6–30). Bioversity International.

[ece36958-bib-0059] Matthews, P. J. (2013). Comparing the habitats of wild rice (*Oryza rufipogon*) and wild taro (*Colocasia esculenta*) in Australia and Papua New Guinea In Yuyao City Hemudu Heritage Museum (Ed.), Hemudu culture international forum: Proceedings (pp. 118–129, in Chinese; pp. 187–204, in English). China Modern Economic Publishing House.

[ece36958-bib-0060] Matthews, P. J. (2014). On the trail of taro: An exploration of natural and cultural history. Senri ethnological studies 88. National Museum of Ethnology.

[ece36958-bib-0061] Matthews, P. J. , Agoo, E. M. G. , Tandang, D. N. , & Madulid, D. A. (2012). Ethnobotany and ecology of wild taro (*Colocasia esculenta*) in the Philippines: Implications for domestication and dispersal In Spriggs M., Addison D., & Matthews P. J. (Eds.), Senri ethnological studies 78: Irrigated taro (*Colocasia esculenta*) in the Indo‐Pacific (pp. 307–340). National Museum of Ethnology.

[ece36958-bib-0062] Matthews, P. J. , Ahmed, I. , Lockhart, P. J. , Agoo, E. M. G. , Naing, K. W. , Nguyen, D. V. , & Medhi, D. K. (2020). DNA sequences for six chloroplast loci concatenated, representing haplotypes found in *Colocasia esculenta*, and closely related Araceae Dryad Dataset, 10.5061/dryad.nvx0k6dpj

[ece36958-bib-0063] Matthews, P. J. , Lockhart, P. J. , & Ahmed, I. (2017). Phylogeography, ethnobotany and linguistics: Issues arising from research on the natural and cultural history of taro, *Colocasia esculenta* (L.) Schott. Man India, 97, 353–380.

[ece36958-bib-0064] Matthews, P. , Matsushita, Y. , Sato, T. , & Hirai, M. (1992). Ribosomal and mitochondrial DNA variation in Japanese taro (*Colocasia esculenta* L. Schott). Japanese Journal of Breeding, 42, 825–833.

[ece36958-bib-0065] Matthews, P. J. , & Naing, K. W. (2005). Notes on the provenance and providence of wildtype taros (*Colocasia esculenta*) in Myanmar. Bulletin of the National Museum of Ethnology, 29, 587–615.

[ece36958-bib-0066] Matthews, P. J. , Nguyen, V. D. , Tandang, D. , Agoo, E. M. , & Madulid, D. A. (2015). Taxonomy and ethnobotany of *Colocasia esculenta* and *C. formosana* (Araceae): Implications for the evolution, natural range, and domestication of taro. Aroideana, 38E, 153–176.

[ece36958-bib-0067] Matthews, P. J. , Takei, E. , & Kawahara, T. (1992). *Colocasia esculenta* var. *aquatilis* on Okinawa Island, southern Japan: The distribution and possible origins of a wild diploid taro. Man and Culture in Oceania, 8, 19–34.

[ece36958-bib-0068] Matthews, P. J. , & Terauchi, R. (1994). The genetics of agriculture: DNA variation in taro and yam In Hather J. G. (Ed.), Tropical archaeobotany: Applications and new developments (pp. 251–270). Routledge.

[ece36958-bib-0069] Miyake, T. , & Yafuso, M. (2005). Pollination of *Alocasia culcullata* (Araceae) by two *Colocasiomyia* flies known to be specific pollinators for *Alocasia odora* . Plant Species Biology, 20, 201–208.

[ece36958-bib-0070] Miyasaka, S. C. , Bellinger, M. R. , Kantar, M. B. , Helmkampf, M. , Wolfgruber, T. , Paudel, R. , & Shintaku, M. (2019). Genetic diversity in taro (*Colocasia esculenta*) In Nandwani D. (Ed.), Genetic diversity in horticultural plants (pp. 191–215). Springer Nature.

[ece36958-bib-0071] Nauheimer, L. (2012). Molecular systematics and historical biogeography of Araceae at a Worldwide scale and in southeast Asia. PhD thesis. Ludwig Maximilian University of Munich, Munich. Retrieved from https://edoc.ub.uni‐muenchen.de/15233/2/Nauheimer_Lars.pdf

[ece36958-bib-0072] Nauheimer, L. , Boyce, P. C. , & Renner, S. S. (2012). Giant taro and its relatives: A phylogeny of the large genus *Alocasia* (Araceae) sheds light on Miocene floristic exchange in the Malesian region. Molecular Phylogenetics and Evolution, 63, 43–51. 22209857 10.1016/j.ympev.2011.12.011

[ece36958-bib-0073] Nauheimer, L. , Metzler, D. , & Renner, S. S. (2012). Global history of the ancient monocot family Araceae inferred with models accounting for past continental positions and previous ranges based on fossils. New Phytologist, 195, 938–950. 22765273 10.1111/j.1469-8137.2012.04220.x

[ece36958-bib-0074] Nguyen, D. V. , Matthews, P. J. , & Ahmed, I. (2016). *Colocasia yunnanensis* (Araceae), a new record for the flora of Vietnam. Journal of Japanese Botany, 91, 223–229.

[ece36958-bib-0075] Nguyen, D. V. , Tran, V. T. , Masuno, T. , & Matthews, P. J. (2015). Useful aroids and their prospects in Vietnam. Aroideana Supplement, 38E, 130–142.

[ece36958-bib-0076] Ochiai, T. , Nguyen, V. X. , Tahara, M. , & Yoshino, H. (2001). Geographical differentiation of Asian taro, *Colocasia esculenta* (L.) Schott, detected by RAPD and isozyme analyses. Euphytica, 122, 219–234.

[ece36958-bib-0077] Ochiai, T. , Tahara, M. , & Yoshino, H. (2000). Phylogenetic relationships of taro and allied species based on restriction fragment length polymorphisms (RFLPs) of chloroplast DNA. Scientific Reports of the Faculty of Agriculture, Okayama University, 89, 15–21.

[ece36958-bib-0078] Plucknett, D. L. (1983). Taxonomy of the genus *Colocasia* In Wang J.‐K. (Ed.), Taro: A review of *Colocasia esculenta* and its potentials (pp. 14–33). University of Hawaii Press.

[ece36958-bib-0079] Scarlett, N. H. (1985). Report on collection trip to the Kimberley and Alice Springs 3rd‐26th July, 1984 [unpublished typescript, Department of Botany, La Trobe University, Melbourne].

[ece36958-bib-0080] Snijder, R. C. , Brown, F. S. , & van Tuyl, J. M. (2007). The role of plastome‐genome incompatibility and biparental plastid inheritance in interspecific hybridization in the genus *Zantedeschia* (Araceae). Floriculture and Ornamental Biotechnology, 1, 150–157.

[ece36958-bib-0081] Spier, R. F. G. (1951). Some notes on the origin of taro. Southwestern Journal of Anthropology, 7, 69–76.

[ece36958-bib-0082] Spriggs, M. (2011). Archaeology and the Austronesian expansion: Where are we now? Antiquity, 85, 510–528. 10.1017/S0003598X00067910

[ece36958-bib-0083] Spriggs, M. , Addison, D. , & Matthews, P. J. (Eds.) (2012). Irrigated Taro (*Colocasia esculenta*) in the Indo‐Pacific. Senri ethnological studies 78. National Museum of Ethnology Retrieved from https://minpaku.repo.nii.ac.jp

[ece36958-bib-0084] Sultana, F. , Hu, Y.‐G. , Toda, M. J. , Takenaka, K. , & Yafuso, M. (2006). Phylogeny and classification of Colocasiomyia (Diptera, Drosophilidae), and its evolution of pollination mutualism with aroid plants. Systematic Entomology, 31, 684–702.

[ece36958-bib-0085] Swofford, D. , Olsen, G. , Waddell, P. , & Hillis, D. (1996). Phylogenetic inference In Hillis D. M., Moritz C., & Mable B. K. (Eds.), Molecular systematics (pp. 407–514). Sinauer.

[ece36958-bib-0086] Tavare, S. (1986). Some probabilistic and statistical problems in the analysis of DNA sequences In Miura R. M. (Ed.), Lectures on mathematics in the life sciences (Vol. 17, pp. 57–86). American Mathematical Society.

[ece36958-bib-0087] Templeton, A. R. (2004). Statistical phylogeography: Methods of evaluating and minimizing inference errors. Molecular Ecology, 13, 789–809. 15012756 10.1046/j.1365-294x.2003.02041.x

[ece36958-bib-0088] Velayudhan, K. C. (2008). Studies on genetic resources of taro – *Colocasia esculenta* (L.) Schott complex. PhD thesis. University of Calicut, Malappuram. Retrieved from https://shodhganga.inflibnet.ac.in/handle/10603/11475

[ece36958-bib-0089] Whitmore, T. C. (1981). Palaeoclimate and vegetation history In Whitmore T. C. (Ed.), Wallace's line and plate tectonics (pp. 36–42). Clarendon Press.

[ece36958-bib-0090] Yen, D. E. , & Wheeler, J. M. (1968). Introduction of taro into the Pacific: The indications of the chromosome numbers. Ethnology, 7, 259–267.

[ece36958-bib-0091] Yoshino, H. (1975). On the wild or escaped and the cultivated species of tribe Colocasieae in eastern Nepal In AACK (Ed.), Yalung Kang science research report (1973 expedition) (pp. 47–61). Academic Alpine Club of Kyoto (AACK).

[ece36958-bib-0092] Yoshino, H. (2002). Morphological and genetic variation in cultivated and wild taro In Yoshida S., & Matthews P. J. (Eds.), Vegeculture in eastern Asia and Oceania (pp. 95–116). JCAS Symposium Series 16. Japan Center for Area Studies.

[ece36958-bib-0093] Zhang, D. , & Zhang, G. (2000). Preliminary studies on evolution and classification of taro (*Colocasia* spp.) in China In Zhu D., Eyzaguirre P. B., Zhou M., Sears L., & Liu G. (Eds.), Ethnobotany and genetic diversity of Asian taro: Focus on China (pp. 32–45). IPGRI Office for East Asia.

[ece36958-bib-0094] Zhang, G. , & Zhang, D. (1990). The relationship between geographic distribution and ploidy level of taro, *Colocasia esculenta* . Euphytica, 47, 25–27. 10.1007/BF00040358

[ece36958-bib-0095] Zhu, D. , Eyzaguirre, P. B. , Zhou, M. , Sears, L. , & Liu, G. (Eds.) (2000). Ethnobotany and genetic diversity of Asian taro: Focus on China. IPGRI Office for East Asia.

