# Supplementary material for: Evolutionary origins of taro (Colocasia esculenta) in Southeast Asia
Source: Ecol Evol. 2020 Nov 2;10(23):13530–43. doi: 10.1002/ece3.6958 (PMC7713977; doi:10.1002/ece3.6958)
Supplement: Supplementary file 1 — Fig S1‐S4 [file ECE3-10-13530-s001.docx]

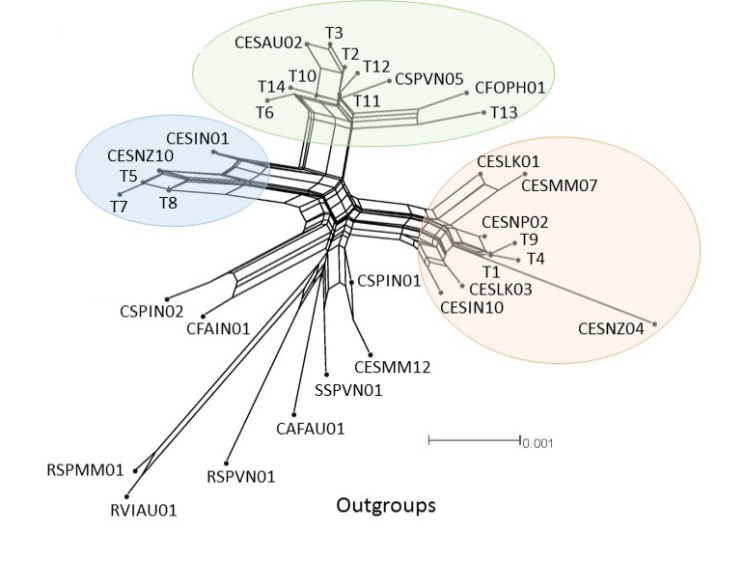


**Supporting Figure 1. Neighbor-net clustering of 34 haplotypes.**

Three main clades are apparent within *C. esculenta* (including *C. formosana*). Color code: orange = Clade I, blue = Clade II, green = Clade III. Genetic distance scale is at right. One Myanmar sample (CESMM12) was identified as *C. esculenta* at time of collection, but appears among the outgroups. Sample set for *C. esculenta* and *C. formosana* as in Fig. 1, but including here the Clade 1 outlier CESNZ04, and further outgroups (as in Supp. Fig. 2).


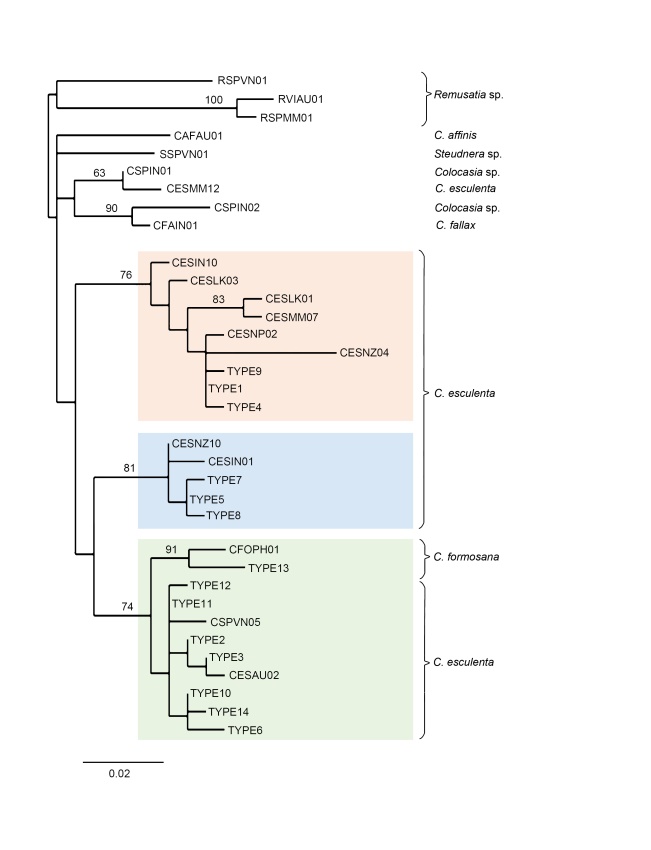


**Supporting Figure 2. Maximum-likelihood tree.**

The tree was made using the concatenated sequences from six chloroplast loci. Bootstrap values above 70 provide support for three main clades within *C. esculenta* (including *C. formosana*). Color code: orange = Clade I, blue = Clade II, green = Clade III. Genetic distance scale is at left. Sample set for *C. esculenta* and *C. formosana* as in Fig. 1, but including here the Clade 1 outlier CESNZ04, and further outgroups (as in Supp. Fig. 1).

*
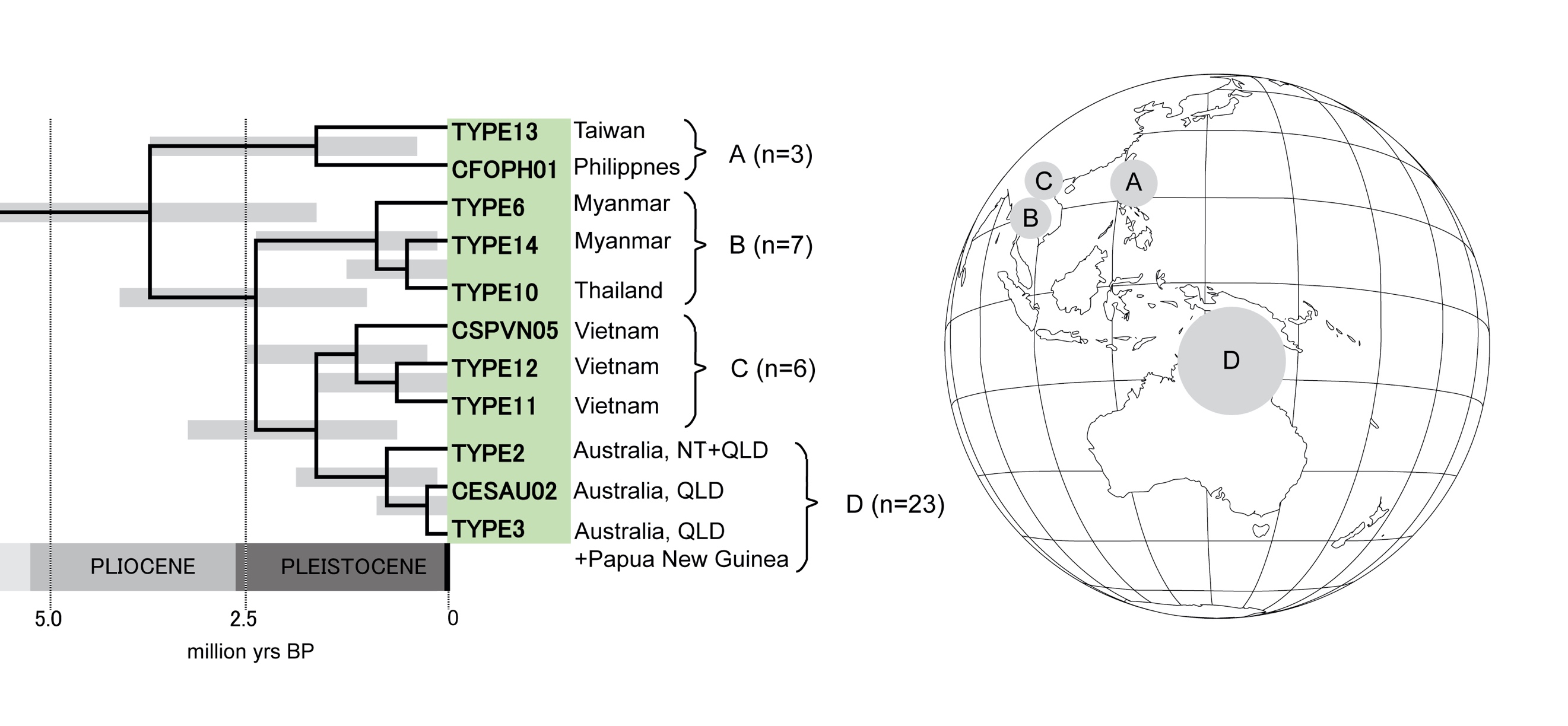
*

**Supporting Figure 3. Geographical distribution of Clade III.**

A = *Colocasia formosana*, B-D = *C. esculenta* and *C.* sp. (CSPVN05; n=1; unique haplotype). CSPVN05, found in an isolated mountain valley in northern Vietnam, is very similar to *C. esculenta* in terms of vegetative morphology, but the floral morphology is not yet known. Although sample sizes within each region are small (Fig. 2), the distribution of subclade diversity within this wild clade is consistent with natural dispersal and differentiation across Southeast Asia during the Pliocene to Pleistocene (natural range expansion accompanied or followed by fragmentation). Diagram at left is a detail from Fig. 1; grey circles at right indicate known geographical extent of each subclade. Total n=39, all samples wild.


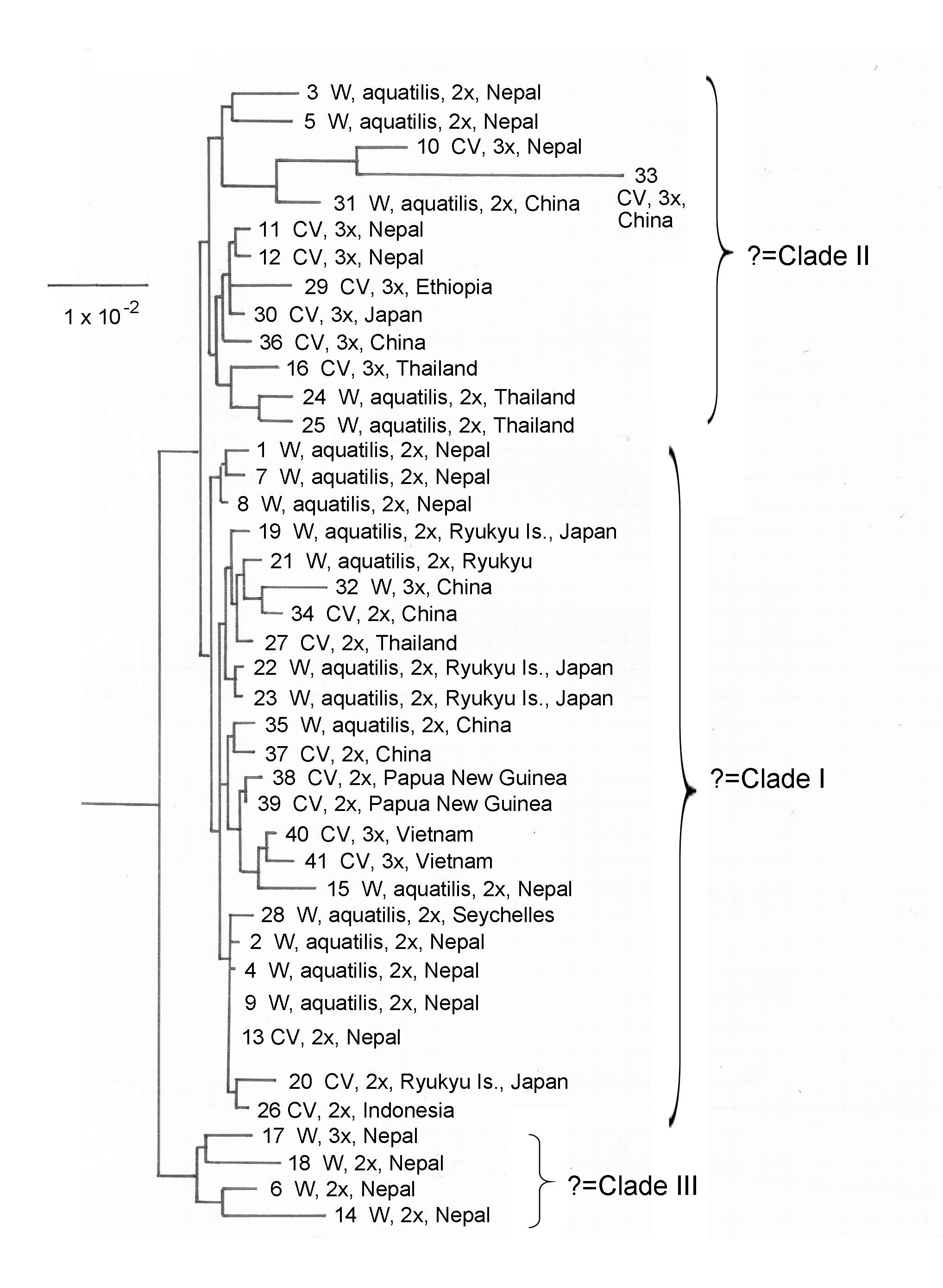


**Supporting Figure 4. Neighbour Joining tree based on RFLP analysis of chloroplast DNA in *Colocasia esculenta*, adapted from Ochiai, Tahara & Yoshino (2000).**

Genetic distance scale is shown at left. Five DNA probes from different regions of a yam chloroplast genome (*Dioscorea* sp.) were hybridised with taro DNA fragments generated by seven restriction enzymes, providing wide coverage of the chloroplast genome. The numbered samples (1-41) were reported as "wild" (W) or as "cultivar" or "cultivated type" (CV), diploid (2x), or triploid (3x) and were mostly from Nepal, China, and Japan. Samples were further identified as either var. *aquatilis* or var. *esculenta*, following Hotta (1970). Distant outgroup taxa shown in the original diagram are not shown here.

The main clusters may correspond to Clades I, II and II reported in the present paper. Samples are not shared with the present study, so the suggested correspondence with clades reported by us (CI, CII, CIII) is tentative only, as explained next.

*Sample cluster 1-26 (? = CI).* This cluster has mainly diploid wild and cultivated plants. In our study, diploid cultivars and commensal wild taros were predominant in CI, and our samples of wild taro (var. *aquatilis*) from Okinawa (nos. 90 to 105, Clade I, Type 1, in Supp. Table 1) come from a regional clonal population that is included in this sample cluster (var. *aquatilis*, Ryukyu Islands). Okinawa is the main island in the Ryukyu archipelago. Wild taros are not known to breed in the Ryukyu archipelago, and are presumed to lie outside the natural range of the species.

*Sample cluster 3-25 (? = CII).* This cluster has mainly triploid cultivars (3x) as well as wild diploids located in mainland Southeast Asia. In our sample set, most known triploids were CII, including samples from Japan, Nepal and Ethiopia. The Nepalese and Ethiopian triploid cultivars may include the same plants sampled by us (CESNP01, CESNP03, and CESET01, Supp. Table 1) from collections previously held at Kyoto University (where much of the work by H. Yoshino was conducted).

*Sample cluster 17-14 (? = CIII).* This cluster of four wild samples from Nepal were noted by Ochiai *et al.* (2000) as possible inter-generic or inter-specific hybrids, with *C. esculenta* as the likely maternal parent. Like our CIII samples, this cluster contains only wild plants. Identification of this cluster as CIII is especially uncertain, as it contains few samples, and these come from outside the geographic range of CIII in our survey.
