# Supplementary material for: Evolutionary origins of taro (Colocasia esculenta) in Southeast Asia
Source: Ecol Evol. 2020 Nov 2;10(23):13530–43. doi: 10.1002/ece3.6958 (PMC7713977; doi:10.1002/ece3.6958)
Supplement: Supplementary file 2 — Table S1‐S3 [file ECE3-10-13530-s002.docx]

Supporting Tables

**Supporting Table 1. List of samples with collection details and individual results (clade, type).**

| List no. | Species^[[1]](#footnote-1)^ | Source  country^[[2]](#footnote-2)^ | Sample  ID^[[3]](#footnote-3)^ | Remarks^[[4]](#footnote-4)^ | Other  numbers^[[5]](#footnote-5)^ | Clade.Type |
| --- | --- | --- | --- | --- | --- | --- |
| **Other genera in Tribe Colocasieae excluding *Colocasia*** | | | | | | |
| 1 | *Remusatia vivipara* | Australia | RVIAU01 | Cape York, Queensland; wild^[[6]](#footnote-6)^, ex Australian National Botanic Gardens, Canberra. Collected before 1990 | ANU T221 | Outgroup |
| 2 | *Remusatia* sp | Myanmar | RSPMM01 | Mt Popa; wild. Coll. PJM & KWN, 27.7.2004; vern. ‘flying taro’ |  | Outgroup |
| 3 | *Remusatia* sp | Vietnam | RSPVN01 | Vic. Sapa, NW Vietnam; wild. Coll. PJM & NVD, 31.07.2012. Possibly *R. yunnanesis* |  | Outgroup |
| 4 | *Steudnera* sp. | Vietnam | SSPVN01 | Ba Vi, Hanoi, N Vietnam; wild. Coll. PJM & NVD, 15.10.2011. Possibly *S. henryana.* |  | Outgroup |
| **Other species in genus *Colocasia* excluding *C. esculenta*** | | | | | |  |
| 5 | *Colocasia* sp. cf. *affinis* | India | CSPIN01 | Gauhati University Campus, Guwahati, Assam; wild. Coll. DKM, 8.08.2011; vern. black-spotted ‘*baga kochu’ (*Assamese*)..* |  | Outgroup |
| 6 | *Colocasia* sp. cf. *affinis* | Myanmar | CSPMM01 | Taik Kyi town, Yangon. Coll. PJM & KWN, 22.07.04; var. A, with purple blotched blade. |  | I.T4 |
| 7 | *Colocasia* sp. cf. *affinis* | Myanmar | CSPMM02 | Taik Kyi town, Yangon. Coll. PJM & KWN, 22.07.04; var. B. |  | I.T4 |
| 8 | *C. affinis*  var. *jenningsii* | Australia | CAFAU01 | *ex culti* England, 1974, via Royal Botanic Gardens, Sydney, to ANU, Canberra c. 1985. | ANU T328 | Outgroup |
| 9 | *C. affinis* | Philippines | CAFPH01 | Growing as ornamental; Luzon, Philippines, 2012. Inflorescence seen, leaf sample collected for the National Herbarium in Manila. |  | I.T1 |
| 10 | *C. fallax* | India | CFAIN01 | Munich Botanic Garden via Vienna Botanic Garden. Coll. JB, before 2010. |  | Outgroup |
| 11 | *C. formosana* | Taiwan | CFOTW01 | Wulu Gorge, Taitung County, SE Taiwan; wild. Coll. ETK, 2008, as seed in fruit; leaf sample from seedling. |  | III.T13 |
| 12 | *C. formosana* | Taiwan | CFOTW02 | Daken, Taichung City, W Taiwan; wild. Coll. PJM, 2008, as seed in fruit; leaf sample from seedling. |  | III.T13 |
| 13 | *C. formosana* | Philippines | CFOPH01 | Ifugao, Mountain prov., wild. Coll. EMA, 2011. |  | III.unique |
| 14 | *C. lihengiae* | Vietnam | CLIVN01 | Vic. Sapa, Lao Cai prov., wild. Coll. PJM & NVD, 31.07.2012. | WP394 | III.T12 |
| 15 | *C. lihengiae* | Vietnam | CLIVN02 | Sapa - Lao Cai road, Lao Cai prov., wild. Coll. PJM & NVD, 01.08.2012. | WP411 | III.T12 |
| 16 | *C. lihengiae* | Vietnam | CLIVN03 | Ba Vi, Hanoi, Lao Cai prov., wild. Coll. PJM & NVD, 15.10.2011. | WP188 | III.T11 |
| 17 | *C. menglaensis* | Vietnam | CMEVN01 | Vic. Sapa, Lao Cai prov., wild. Coll. PJM & NVD, 31.08.2012. | WP019 | III.T12 |
| 18 | *C. menglaensis* | Vietnam | CMEVN02 | Sapa–Lao Cai road, Lao Cai prov., wild. Coll. PJM & NVD, 31.07.2012. | WP395 | III.T12 |
| 19 | *C. menglaensis* | Vietnam | CMEVN03 | Ba Vi, Hanoi, wild. Coll. PJM & NVD, 15.10.2011. | WP188 | III.T11 |
| 20 | *C. yunnanensis* | Vietnam | CYUVN01 | Vic. Sapa, Lao Cai prov., wild. Coll. PJM & NVD, 21.10.2011; variegated blade. | WP267 | III.T11 |
| 21 | *C. yunnanensis* | Vietnam | CYUVN02 | Vic. Sapa, Lao Cai prov., wild. Coll. PJM & NVD, 21.10.2011; green blade. | WP267 | III.T11 |
| 22 | *C. yunnanensis* | Vietnam | CYUVN03 | Vic. Sapa, Lao Cai prov., wild. Coll. PJM & NVD, 21.10.2011; green blade. | WP271 | III.T11 |
| 23 | *C. yunnanensis* | Vietnam | CYUVN04 | Vic. Sapa, road to Khoang vill., Lao Cai prov., wild. Coll. PJM & NVD, 21.10.2011; green blade. | WP274 | III.T11 |
| 24 | *C. yunnanensis* | Vietnam | CYUVN05 | Vic. Sapa, Lao Cai prov., wild. Coll. PJM & NVD, 22/10/11; green blade. | WP286 | III.T11 |
| 25 | *C.* sp*.* ‘Mau son’ | Vietnam | CSPVN01 | Mau Son, Lang Son prov., wild. Coll. PJM & NVD, 08.08.2012. | WP456 | III.T11 |
| 26 | *C.* sp*.* ‘Mau son’ | Vietnam | CSPVN02 | Mau Son, Lang Son prov., wild. Coll. PJM & NVD, 09.08.2012. | WP458 | III.T11 |
| 27 | *C.* sp. ‘Mau son’ | Vietnam | CSPVN03 | Khat cap-1, Mau Son, Lang Son prov., wild. Coll. PJM & NVD, 09.08.2012. | WP462 | III.T11 |
| 28 | *C.* sp. ‘Mau son’ | Vietnam | CSPVN04 | Khay cap-2, Mau Son, Lang Son prov., wild. Coll. PJM & NVD, 09.08.2012. | WP463 | III.T11 |
| 29 | *C.* sp. ‘Bing lu’ | Vietnam | CSPVN05 | Hó Chäu Ba, Bing lu District, Lai Chau prov., wild. Coll. PJM & NVD, 27.10.2011. | WP288 | III.unique |
| ***Colocasia esculenta*** | | | | | | |
| 30 | Wild | Australia | CESAU01 | 80 km E of Maningrida, on trib. of Blyth R., Arnhem Land, Northern Territory. Coll. RJ, 1980. | ANU T31 | III.T2 |
| 31 | Wild | Australia | CESAU02^[[7]](#footnote-7)^ | Stewart Creek, Daintree R. tributary, Queensland. Coll. JW& IT, 11.06.1972; 2*n* = 28. | ANU T33 | III.unique |
| 32 | Wild | Australia | CESAU03 | Ngilipitji, Walker R., Arnhem Land, Northern Territory. Coll. NW, 18.08.1983 (same location as CESAU05). | ANU T331 | III.T2 |
| 33 | Wild | Australia | CESAU04 | Wurlwurlji, Osmund Range, Kimberley, West Australia. Coll. NS, 04.07.1984; 2*n* = 28. | ANU T335 | I.T1 |
| 34 | Wild | Australia | CESAU05 | Ngilipitji, Walker R., Arnhem Land, Northern Territory. Coll. NW, 09.07.1981 (same location as CESAU03). | NGW81-6, ANU T338 | III.T2 |
| 35 | Wild | Australia | CESAU06 | Upper Calvert R., Northern Territory, c. 100 km from Gulf of Carpentaria. Coll. PL, 1986; 2*n* = 28. | ANU T376 | III.T2 |
| 36 | Cultivar, naturalized (wild) | Australia | CESAU07 | Lex Creek Rd, vic. Eungella NP, Queensland. Coll. PJM, 11.09.1987, phenotype 1. |  | I.T1 |
| 37 | Wild | Australia | CESAU08 | Gap Creek, Halifax Bay, Queensland. Coll. PJM, 16.09.1987. |  | III.T3 |
| 38 | Wild | Australia | CESAU09 | Little Gin Creek, Halifax Bay, Queensland. Coll. PJM, 16.09.1987. |  | III.T2 |
| 39 | Wild | Australia | CESAU10 | Jiyer Cave, Russell R., Queensland. Coll. PJM, 20.09.1987, leaf J2C; 2*n* = 28. |  | III.T3 |
| 40 | Wild | Australia | CESAU11 | Whyanbeel Creek, Queensland. Coll. PJM, 29.09.1987; leaf N3. |  | III.T3 |
| 41 | Wild | Australia | CESAU12 | Cassowary Creek, Queensland. Coll. PJM, 30.9.87; leaf N2 |  | III.T3 |
| 42 | Wild | Australia | CESAU13 | South Mossman R., Queensland. Coll. PJM, 30.09.1987; leaf N1. |  | III.T3 |
| 43 | Wild | Australia | CESAU14 | Harvey Creek, Mulgrave R. trib., Queensland. Coll. PJM, 01.10.1987; leaf N1. |  | III.T3 |
| 44 | Wild | Australia | CESAU15 | Boulder Falls, North Babinda Creek, Queensland, Coll. PJM, 02.10.1987; leaf N1. |  | III.T3 |
| 45 | Wild | Australia | CESAU16 | Badgery Creek, Borong State Forest, Queensland. Coll. PJM, 02.10.1987; leaf N1. |  | III.T3 |
| 46 | Wild | Australia | CESAU17 | Blue's Patch, lower Seymour R., Queensland. Coll. PJM, 03.10.1987; leaf N1. |  | III.T3 |
| 47 | Wild | Australia | CESAU18 | Combo's Crossing, Russell R. Coll. PJM, 21.09.1987; leaf N1. |  | III.T3 |
| 48 | Wild | Australia | CESAU19 | Combo's Crossing, Russell R. Coll. PJM, 21.09.1987; leaf N2. |  | III.T3 |
| 49 | Cultivar, (naturalized wild) | Australia | CESAU20 | Mooball, New South Wales. Coll. PJM 07.10.1987; leaf N3. |  | I.T1 |
| 50 | Wild | Australia | CESAU21 | Hopevale, Queensland. Coll. PJM, 26.09.1987; leaf U1.1 from breeding population (= P1 in Hunt, Moots, & Matthews, 2013), so presumed diploid. |  | III.T2 |
| 51 | Wild | Australia | CESAU22 | Hopevale, Queensland. Coll. PJM, 26.09.1987; leaf U6.3 from breeding population (= P9 in Hunt, Moots, & Matthews, 2013), so presumed diploid. |  | III.T3 |
| 52 | Wild | Australia | CESAU23 | Hopevale, Queensland. Coll. PJM, 26.09.1987; leaf B1.1 from breeding population (= P31 in Hunt, Moots, & Matthews, 2013), so presumed diploid. |  | III.T2 |
| 53 | Wild | Bangladesh | CESBD01 | Vic. Dakar, Bangladesh. Coll. KI, 08.2009. |  | I.T4 |
| 54 | Cultivated | Chile | CESEI01 | Rapanui (Easter Island). Coll. DEY, 1984, #1; DNA extracted 12.06.1987; vern. *ketu* *anga mea*, faint pink at top of petiole. | ANU T114 | I.T1 |
| 55 | Cultivated | Chile | CESEI02 | Rapanui (Easter Island). Coll. DEY, 1984, #4; vern. *ara vatea,* pink markings; 2*n* = 28. | ANU T117 | I.T1 |
| 56 | Cultivated | Chile | CESEI03 | Rapanui (Easter Island). Coll. DEY, 1984, #6; vern. *hara hara rapanui*, pink stem. | ANU T119 | I.T1 |
| 57 | Cultivated | Cyprus | CSPCY01 | Via migrants from Cyprus to Whanganui. Coll. PJM, 29.05.2002. |  | I.T1 |
| 58 | Cultivar | Egypt | CESJP02 | Cairo market, Egypt, via PJM, Japan, 03.11.2010; vern. *qolqas, ul’as* (Arabic). |  | I.T1 |
| 59 | Cultivar | Ethiopia | CESET01 | Via Plant Systematics Laboratory, Kyoto University, 1994. |  | II.T5 |
| 60 | Cultivar | Fiji | CSPFJ01 | Via F. Delsuc, 2010, as ‘*Colocasia* sp.’, presumed *C. esculenta*, garden or naturalized (no other sp. recorded in Fiji). |  | I.T1 |
| 61 | Cultivar | Fiji | CSPFJ02 | Via F. Delsuc, 2010, as ‘*Colocasia* sp.’, presumed *C. esculenta,* garden or naturalized (no other sp. recorded in Fiji). |  | I.T1 |
| 62 | Cultivar | Fiji | CSPFJ03 | Via F. Delsuc, 2010, as ‘*Colocasia* sp.’, presumed *C. esculenta,* garden or naturalized (no other sp. recorded in Fiji). |  | I.T1 |
| 63 | Cultivar | Fiji | CSPFJ05 | Via F. Delsuc, 2010, as ‘*Colocasia* sp.’, presumed *C. esculenta,* garden or naturalized (no other sp. recorded in Fiji). |  | I.T1 |
| 64 | Cultivated | French Polynesia | CESSI01 | Huahine, Society Islands. Coll. DEY, #11, 1984; vern. *'apura'*, medium green stem; 2*n* = 28. | ANU T124 | I.T1 |
| 65 | Cultivated | French Polynesia | CESSI02 | Huahine, Field # 13, Society Islands. Coll. DEY. DNA ext., 20.06.1988; vern. ‘*veo*’, stem medium light green. | ANU T 126 | I.T1 |
| 66 | Cultivated | French Polynesia | CESSI03 | Huahine, Field #14, Society Islands. Coll. DEY. DNA ext., 20.06.1988; vern. *'iihi'*, stem dark purple with green stripes. | ANU T 127 | I.T1 |
| 67 | Cultivated | Fiji (from India) | CESIN01 | Via Lyon Arboretum, Honolulu. Coll. DEY, 31.08.1983, orig. coll. Fiji 1963; 2*n* = 42, supplied by Indian growers in Fiji (Yen & Wheeler, 1968; Coates, Yen & Gaffey, 1988). | ANU T111 | II.unique |
| 68 | Cultivated | India | CESIN02 | Hamren, Karbi Anglong, Assam. Coll. DKM, 22.05.2011; vern. *banaria boga kochu* (Assamese). |  | I.T4 |
| 69 | - | India | CSPIN02  (likely *C. esculenta*) | Langhemphi, Karbi Aong, Coll. DKM, 22.05.2011; vern. *harlong bongvo* (Karbi). |  | I.T4 |
| 70 | Cultivated | India | CESIN03 | Hamren, Karbi Anglong, Assam. Coll. DKM, 22.05.2011; vern. *boga kochu* (Mukhiya) (Assamese). |  | 1.T1 |
| 71 | Cultivated | India | CESIN06 | Umpavang, Karbi Anglong. Coll. DKM, 22.05.2011; vern. *hensek* (Karbi). |  | I.T1 |
| 72 | Cultivated | India | CESIN07 | Nongjrong, Karbi Anglong. Coll. DKM, 22.05.2011; vern. *chaore kochu* (Nepali), *hensek voso* (Karbi). |  | I.T4 |
| 73 | Wild (commensal) | India | CESIN08 | Gurchok, Pathar Quarry, Guwahati. Coll. DKM, 06.07.2011; vern. *boga kochu* (Assamese). |  | I.T4 |
| 74 | Wild (commensal) | India | CESIN09 | Deepar Beel, Guwahati, Assam; near rail gate (crossing). Coll. DKM, 06.07.2011; vern. *garo kochu* (Assamese). |  | I.T1 |
| 75 | Wild | India | CESIN10 | Vic. CRPF camp, Rani Forest, Assam. Coll. DKM, 06.07.2011; vern. *garo kochu* (Assamese); black plant. |  | I.unique |
| 76 | Cultivated | India | CESIN11 | Vic. guest house, Rani Forest. Coll. DKM, 06.07.2011; vern. *nal kochu;* pink strip on petiole. |  | I.T4 |
| 77 | Wild (commensal) | India | CESIN12 | Campus, Gauhati University, Guwahati, Assam. Coll. DKM, 08.08.2011; vern. *nal kochu bor* (big). |  | I.T4 |
| 78 | Cultivated | Japan | CESJP01 | Kyoto. Coll. PJM, 23.11.2005; vern. cv. *tono-imo* (lit. ‘taro of China’); diploid cv in Matthews, Matsushita et al. (1992). |  | I.T1 |
| 79 | Cultivated | Japan | CESJP03 | Kyoto. Coll. PJM, 07.09.2010; vern. cv. *Ishikawa-wase;* triploid cv in Matthews, Matsushita et al. (1992). |  | II.T5 |
| 80 | Cultivated | Japan | CESJP04 | Kyoto. Coll. PJM, 07.09.2010; vern. cv. *dodare*; triploid cv in Matthews, Matsushita et al. (1992). |  | II.T5 |
| 81 | Cultivated | Japan | CESJP05 | Kyoto. Coll. PJM, 07.09.2010; vern. cv. *tono-imo*; diploid cv in Matthews, Matsushita et al. (1992). |  | I.T1 |
| 82 | Cultivated | Japan | CESJP06 | Kyoto. Coll. PJM, 07.09.2010; vern. cv. *kinu-hikari.* |  | II.T5 |
| 83 | Cultivated | Japan | CESJP07 | Kyoto. Coll. PJM, 07.09.2010; vern. cv. *kamisho.* |  | II.T5 |
| 84 | Cultivated | Japan | CESJP08 | Kyoto. Coll. PJM, 07.09.2010; vern. cv. 'Kyoto strong'. |  | II.T5 |
| 85 | Cultivar | Japan | CESJP09 | Minami-ibaraki, Osaka. Coll. PJM, 11.11.2010; vern. cv. *aka-zuiki.* |  | I.T1 |
| 86 | Cultivated | Japan | CESJP10 | Kawakami Aza, Okinawa. Coll. PJM 19.05.2011; vern. cv. *ta-imo.* | WP088 | I.T1 |
| 87 | Cultivated | Japan | CESJP11 | Kagoshima Prefecture Agricultural Development Centre, Osumi Branch, Kyushu. Coll. Kik, 30.06.2011; cv. *binroshin*; historically known as introduction from Taiwan; diploid cv. in Matthews, Matsushita et al. (1992). |  | I.T1 |
| 88 | Cultivar | Japan | CESJP12 | Osaka City, ex Miyazaki Prefecture, Kyushu. Coll. ET, 10.12.2010; vern. cv. *akame* ; triploid cv. in Matthews, Matsushita et al. (1992). |  | II.T5 |
| 89 | Cultivar | Japan | CESJP13 | Osaka Prefecture. Coll. ET, 10.12.2010; cv. *egu-imo;* triploid cv in Matthews, Matsushita et al. (1992). |  | II.T5 |
| 90 | Wild (commensal) | Japan | CESJP146 | Todoroki road, Okinawa. Coll. PJM, 17.05.2011, vern. *maa-muun.* | WP048 | I.T1 |
| 91 | Wild (commensal) | Japan | CESJP15 | Todoroki waterfall. Coll. PJM, 17.05.2011. | WP048 | I.T1 |
| 92 | Wild (commensal) | Japan | CESJP16 | Vic. Bimata tofu factory, Okianwa. Coll. PJM, 18.05.2011. | WP052 | I.T1 |
| 93 | Wild (commensal) | Japan | CESJP17 | Vic. Bimata Yabu primary school, Nakayama, Okinawa. Coll. PJM, 18.05.2011. | WP053 | I.T1 |
| 94 | Wild (commensal) | Japan | CESJP18 | Vic. Mazakina bus stop, Okinawa. Coll. PJM, 18.05.2011. | WP054 | I.T1 |
| 95 | Wild (commensal) | Japan | CESJP19 | Inoha landslide, Okinawa. Coll. PJM, 18.05.2011. | WP059 | I.T1 |
| 96 | Wild (commensal) | Japan | CESJP20 | Yaedake Road, Okinawa. Coll. PJM, 18.05.2011. | WP065 | I.T1 |
| 97 | Wild (commensal) | Japan | CESJP21 | Yaedake Lookout, Okinawa. PJM, 18.05.2011. | WP070 | I.T1 |
| 98 | Wild (commensal) | Japan | CESJP22 | Asahikawa, Okinawa. Coll. PJM, 18.05.2011 | WP074 | I.T1 |
| 99 | Wild (commensal) | Japan | CESJP23 | Okita, Nago City, Okinawa. Coll. PJM, 19.05.2011. | WP084 | I.T1 |
| 100 | Wild (commensal) | Japan | CESJP24 | Lower Isashigawa Valley, Okinawa. Coll. PJM, 19.05.2011. | WP086 | I.T1 |
| 101 | Wild (commensal) | Japan | CESJP25 | Upper Kawakami, Okinawa. Coll. PJM, 19.05.2011. | WP089 | I.T1 |
| 102 | Wild (commensal) | Japan | CESJP26 | Makiya-okawa Valley, Okinawa. Coll. PJM, 19.05.2011. | WP094 | I.T1 |
| 103 | Wild (commensal) | Japan | CESJP27 | Makiya-okawa Dam, Okinawa. Coll. PJM, 19.05.2011. | WP092 | I.T1 |
| 104 | Wild (commensal) | Japan | CESJP28 | Sub-location a, Fukuchigawa, Okinawa. Coll. PJM, 20.05.2011. | WP105 | I.T1 |
| 105 | Wild (commensal) | Japan | CESJP29 | Sub-location b, Fukuchigawa, Okinawa. Coll. PJM, 20.05.2011. | WP105 | I.T1 |
| 106 | Cultivar | Madagascar | CESMG01 | Madagascar. Coll. HEW, 1988; with cormels. | ANU T363 | II.T5 |
| 107 | Cultivar | Madagascar | CESMG02 | Madagascar. Coll. HEW, 1988; with cormels. | ANU T364 | II.T5 |
| 108 | Cultivar | Madagascar | CESMG03 | Madagascar. Coll. HEW, 1988; with cormels. | ANU T365 | II.T5 |
| 109 | Cultivar | Madagascar | CESMG04 | Madagascar. Coll. HEW, 1988; with direct side-shoots and short stolons. | ANU T366 | I.T1 |
| 110 | Wild | Myanmar | CESMM02 | Forest stream below Kyauk-than-bud vill., vic. Yezin. Coll. PJM & KWN, 30.07.04. | WP071 | III.T6 |
| 111 | Wild (commensal) | Myanmar | CESMM03 | Near Botataung Monastery (vic. Yodega Gate), Yangon. Coll. PJM & KWN, 21.07.04. |  | I.T4 |
| 112 | Wild | Myanmar | CESMM06 | Yuzana Spring, Mt Popa. Coll. PJM & KWN, 26.07.04. |  | III.T14 |
| 113 | Wild | Myanmar | CESMM07 | Ngayangon Spring, Mt Popa. Coll. PJM & KWN, 27.07.2004. |  | I.unique |
| 114 | Wild | Myanmar | CESMM08 | Tha Kho Kya spring, Mt Popa. Coll. PJM & KWN, 26.07.2004. |  | III.T14 |
| 115 | Wild (commensal) | Myanmar | CESMM09 | Tadau vill., Bago Region. Coll. PJM & KWN, 31.07.2004; green stem. | WP080 | I.T4 |
| 116 | Wild | Myanmar | CESMM10 | River bank at foot of forested hills, Tan Pin Chaung, Yezin. Coll. PJM & KWN, 29.07.2004. |  | III.T6 |
| 117 | Wild (commensal) | Myanmar | CESMM11 | Riverside below gardens, vic. Let Pan Dan, Yangon-Pyay Road. Coll. PJM & KWN, 22.07.2004. |  | I.T1 |
| 118 | Wild (commensal) | Myanmar | CESMM12 | Zi Gon town, Yangon-Pyay Road. Coll. PJM & KWN, 22.07.2004; vern. *pein*; very pale, bright-green blade and light-purple petiole; not eaten; used as pig fodder. |  | Outgroup |
| 119 | Wild (commensal) | Myanmar | CESMM13 | Kyauktan, vic. Yangon. Coll. PJM & KWN, 01.08.2004. |  | I.T4 |
| 120 | Cultivar | Nepal | CESNP01 | Bazar, Kathmandu. Coll. TY, 21.06.1973; vern. *pindalu*; 2*n* = 42. | KPGI 7416  ANU T307 | II.T7 |
| 121 | Cultivar | Nepal | CESNP02 | Bazar, Kathmandu. Coll. SS, 03.10.1975; 2*n* = 42. | KPGI 7506  ANU T309 | I, unique |
| 122 | Cultivar | Nepal | CESNP03 | Bazar, Kathmandu. Coll. SS, 03.10.1975; 2*n* = 42. | KPGI 7511  ANU T310 | II.T5 |
| 123 | Cultivated  (ornamental, cf. cv "Black Magic") | New Zealand | CESNZ01 | No provenance; campus, University of Auckland, Auckland. Coll. IA, 25.06.2008; purple. | MPN 46547 | I.T1 |
| 124 | - (cultivar) | New Zealand | CESNZ02 | No provenance; campus, University of Auckland, Auckland. Coll. IA, 25.06.2008; var. RR, triploid, cf. Matthews (2014). | MPN 46548; cp genome sequenced; GenBank JN105690 | II.T5 |
| 125 | - | New Zealand | CESNZ03 | No provenance; campus, University of Auckland. Coll. IA, 25.06.2008; var. GP; triploid, cf. Matthews (2014). | MPN 46549; cp genome sequenced;  GenBank JN105689 | I.T4 |
| 126 | Cultivated  (ornamental, cf. cv "Black Magic") | New Zealand | CESNZ04 | No provenance; campus, University of Auckland. Coll. IA, 25.06.2008; purple. |  | I.outgroup  (figs. S2; S3) |
| 127 | - (cultivar) | New Zealand | CESNZ05 | No provenance; campus, University of Auckland. Coll. IA, 25.06.2008; var. RR; triploid, cf. Matthews (2014). |  | II.T5 |
| 128 | Wild (naturalized cultivar) | New Zealand | CESNZ06 | Rodney district, Auckland. Coll. VT, 2003; var. RR, 'heritage variety'; triploid, cf. Matthews (2014). | ABG 20040027 | II.T5 |
| 129 | Cultivated | New Zealand | CESNZ07 | Rodney district, Auckland. Coll. IL 2003; var. RR, 'heritage variety'; triploid, cf. Matthews (2014). | ABG 20040028 | II.T5 |
| 130 | Wild (commensal) | New Zealand | CESNZ08 | Franklin district. Coll. IL, 2003; var. GP, 'heritage variety'; triploid, cf. Matthews (2014). | ABG 20040025 | I.T4 |
| 131 | Wild (naturalized cultivar) | New Zealand | CESNZ09 | Waiheke Island, Auckland. Coll. VT; var. RR, 'heritage variety'; triploid, cf. Matthews (2014). | ABG 20040498. | II.T5 |
| 132 | - (cultivar) | New Zealand | CESNZ10 | No provenance, ABG. Var. RR; triploid, cf. Matthews (2014). | ABG 20040201 | II.unique |
| 133 | Wild (naturalized cultivar) | New Zealand | CESNZ11 | Rodney district, Auckland. Coll. IL, 2003; var. RR, 'heritage variety'; triploid, cf. Matthews (2014). | ABG 20040030 | II.T5 |
| 134 | Wild (naturalized cultivar) | New Zealand | CESNZ12 | Auckland City. Coll. VT, 2004; var. RR; triploid, cf. Matthews (2014). | ABG 20040499 | II.T5 |
| 135 | - | New Zealand | CESNZ13 | No provenance, ex Opanaku Nursery, Henderson, Auckland, via ABG. | ABG 931024 | I.T1 |
| 136 | Cultivated (ornamental) | New Zealand | CESNZ14 | Tandara Nursery, Auckland, via ABG. Var. *fontanesii*; triploid, cf. Figs. 10.2 & 14.11 in Matthews (2014). | ABG 20040128 | I.T1 |
| 137 | Cultivated (ornamental) | New Zealand | CESNZ15 | Campus, Massey University, Palmerston North. Coll. IA, 27.06.2008; var. *fontanesii*; triploid, cf. Figs. 10.2 & 14.11 in Matthews (2014). |  | I.T1 |
| 138 | Wild (commensal) | New Zealand | CESNZ16 | Grazed wetland; vic. Otaika Bridge, Whangarei. Coll. PJM & IA, 23.10. 2009; var. GP; triploid, cf. Matthews (2014). |  | I.T4 |
| 139 | Wild (naturalized cultivar) | New Zealand | CESNZ17 | Mimiha stream, Helena Bay, Whangerei. Coll. PJM & IA, 23.10. 2009; var. RR; triploid, cf. Matthews (2014). |  | II.T5 |
| 140 | Wild (naturalized cultivar) | New Zealand | CESNZ18 | Vic. Rawhiti settlement, Oke Bay, Bay of Islands, Northland. Coll. PJM & IA, 24.10. 2009; var. RR; triploid, cf. Matthews (2014). |  | II.T5 |
| 141 | Cultivated | New Zealand | CESNZ19 | Waipiro Bay, Bay of Islands Coll. PJM & IA, 24.10. 2009; var. RR; triploid, cf. Matthews (2014). |  | II.T5 |
| 142 | Cultivated (ornamental) | New Zealand | CESNZ20 | Peka Peka stream, Ohaeawai, Bay of Islands. Coll. PJM & IA, 26.10. 2009; var. *fontanesii*; triploid, cf. Figs. 10.2, 14.11 in Matthews (2014). | MPN 46550 | I.T1 |
| 143 | Wild (naturalized cultivar) | New Zealand | CESNZ21 | Peka Peka stream, Ohaeawai, Bay of Islands. Coll. PJM & IA, 26.10. 2009; var. RR;, triploid, cf. Matthews (2014). |  | II.T5 |
| 144 | Wild (naturalized cultivar) | New Zealand | CESNZ22 | Taupo Bay. Coll. TK, Oct. 2010; var. RR; triploid, cf. Matthews (2014). |  | II.T8 |
| 145 | Wild (naturalized cultivar) | New Zealand | CESNZ23 | Okiwi, Great Barrier Island, Auckland. Coll. PJM, 23.02.2011; var. RR; triploid, cf. Matthews (2014). |  | II.T8 |
| 146 | Cultivated | Pakistan | CESPK01 | Tarbela, District Haripur. Coll. IA, May 2009; vern. *arvi*, | UAAR no. 125749 | II.T7 |
| 147 | Cultivated | Pakistan | CESPK02 | District Qasoor. Coll. IA, June 2009; vern. *arvi*. | UAAR no. 125750 | II.T7 |
| 148 | Cultivated | Pakistan | CESPK03 | Hasan Abdal, District Attock. Coll. IA, May 2009; vern. *arvi*. | UAAR no. 125751 | II.T5 |
| 149 | Cultivated | Pakistan | CESPK04 | District Pakpattan. Coll. IA, June 2009; vern. *arvi*. | UAAR no. 125752 | II.T5 |
| 150 | Cultivated | Pakistan | CESPK05 | District Multan. Coll. IA, June 2009; vern. *arvi*. | UAAR no. 125753 | II.T7 |
| 151 | Cultivated | Pakistan | CESPK06 | Jalalia (Hazro), District Attock. Coll. IA, May 2009; vern. *arvi*. | UAAR no. 125754 | II.T7 |
| 152 | Cultivated | Pakistan | CESPK07 | Jahangira, District Nowshehra. Coll. IA, May 2009; vern. *arvi*. | UAAR no. 125755 | II.T7 |
| 153 | Wild | Papua New Guinea | CESPG01 | Kerevat, East New Britain, Coll. in Yen 1981 list; inedible, in & near water courses; 2*n* = 28. | ANU T19 | III.T3 |
| 154 | Wild | Papua New Guinea | CESPG02 | Goldie R., near Port Moresby. Coll. DEY, in 1981 list; vern. *sabaka.* | ANU T21 | I.T1 |
| 155 | Wild | Papua New Guinea | CESPG03 | Goldie R., near Port Moresby. Coll. DEY, in 1981 list; vern. *apa*; 2*n* = 28. | ANU T24 | I.T1 |
| 156 | Wild | Papua New Guinea | CESPG04 | Yeni, Ruti, Western Highlands prov. Coll. DEY, July 1985, field #R11, vern. *me kekligh*; near garden site (looks like Queensland wild taro, PJM). | ANU T167 | I.T1 |
| 157 | Wild | Papua New Guinea | CESPG05 | Lower Wau Road, Markham Valley; site 29.6/3. Coll. PJM, 29.6.1985. | ANU T227 | I.T1 |
| 158 | Wild | Papua New Guinea | CESPG06 | Ramu R., Markham Valley, site 29.6/5. Coll. PJM, 29.6.1985; 2*n* =28, Fig. 10.10 in Matthews (2014). | ANU T229 | III.T3 |
| 159 | Wild | Papua New Guinea | CESPG07 | Vic. Bubia, Markham Valley, Morobe prov. Coll. PJM, 29.6.85. |  | III.T3 |
| 160 | Cultivated | Papua New Guinea | CESPG08 | Tambul, vic. Wahgi Valley, via Kuk Agricultural Station. Coll. DEY, in 1981 list; orig. coll. JP; vern. cv. *kawaro* (Kakoli lang.); 2*n* = 28. | ANU T1 | I.T1 |
| 161 | Cultivated | Papua New Guinea | CESPG09 | Kainantu, via Highlands Agricultural Experiment Station (HAES), Aiyura. Coll. DEY, in 1981 list; vern. cv. *misio'o*; 2*n* = 28. | ANU T11 | I.T1 |
| 162 | Cultivated | Papua New Guinea | CESPG10 | Goldie R., near Port Moresby. Coll. Yen, in 1981 list; vern. cv. *budoa*; also ‘feral’; 2*n* = 28. | ANU T23 | I.T1 |
| 163 | Cultivated | Papua New Guinea | CESPG11 | Mt Orop-Rulna, vic. Wahgi Valley. Coll. DW, 1981–82; vern. cv. *dampel (*Melpa), an ‘old’ variety. | ANU T46 | I.T1 |
| 164 | Cultivated | Papua New Guinea | CESPG12 | Wunimp, vic. Wahgi Valley. Coll. DW, 1981–82; vern. cv. *mit/deng* (Melpa), a ‘new’ variety. | ANU T47 | I.T1 |
| 165 | Cultivated | Papua New Guinea | CESPG13 | Wana Wunimp, Rulna. Coll. DW, 1981–82; vern. cv. *rom* (Melpa); ‘old’ variety. | ANU T48 | I.T1 |
| 166 | Cultivated | Papua New Guinea | CESPG14 | Mt Orop, vic. Wahgi Valley. Coll. DW, 1981–82; vern. cv. *kimba* (Melpa); ‘old’ variety. | ANU T49 | I.T1 |
| 167 | Cultivated | Papua New Guinea | CESPG15 | Near Wanimp crater, vic. Wahgi Valley. Coll. DW, 1981–’82; ‘old’ Melpa variety. | ANU T50 | I.T1 |
| 168 | Cultivated | Papua New Guinea | CESPG16 | Ruti, Kawbenaberi, Western Highlands. Coll. DEY, June/July 1985. | ANU T160 | I.T1 |
| 169 | Cultivated | Papua New Guinea | CESPG17 | Ruti, near Yeni swamp, Western Highlands. Coll. DEY, July 1985, vern. cv. *kun.* | ANU T 163  (field #R7) | I.T1 |
| 170 | Cultivated | Papua New Guinea | CESPG18 | Ruti, near Yeni swamp, Western Highlands. Coll. DEY, July 1985, vern. cv. *kimbe.* | ANU T164  (field #R8) | I.T1 |
| 171 | Cultivated | Papua New Guinea | CESPG19 | Ruti, near Yeni swamp, Western Highlands. Coll. DEY, July 1985, ‘local cv.’ | ANU T166  (field #R10) | I.T1 |
| 172 | Cultivated | Philippines | CESPH01 | via Lyon Arboretum (no number), Hawaii. Coll. DEY, 31.08.1983; orig. coll. Philippines, 1963. | ANU T107 | I.T1 |
| 173 | Cultivated | Philippines | CESPH02 | Bayninan, Banaue, Ifugao, Luzon. Coll. DEY & HC, 1985; vern. *mumpaahaq;* leaf & corm eaten. | ANU T131  (field #2) | I.T1 |
| 174 | Cultivated | Philippines | CESPH03 | Bayninan, Banaue, Ifugao, Luzon. Coll. DEY & HC, 1985; inf. #2, vern. *baakun;* leaf & corm eaten. | ANU T134  (field #20) | I.T1 |
| 175 | Cultivated | Philippines | CESPH04 | Bayninan, Banaue, Ifugao, Luzon. Coll. DEY & HC, 1985; vern. *quingallitan;* leaf & corm eaten. | ANU T137  (field #5) | I.T1 |
| 176 | Cultivated | Philippines | CESPH05 | Bayninan, Banaue, Ifugao, Luzon. Coll. DEY & HC, 1985; vern. *pehen;* leaf only eaten. | ANU T139 (field #8, ‘duplicate of field #16’) | I.T1 |
| 177 | Cultivated | Philippines | CESPH06 | Bayninan, Banaue, Ifugao, Luzon. Coll. DEY & HC, 1985; inf. #2; vern. *pehen;* leaf only eaten. | ANU T140 (field #16, ‘duplicate of field #8’) | I.T9 |
| 178 | Cultivated | Philippines | CESPH07 | Bayninan, Banaue, Ifugao, Luzon. Coll. DEY & HC, 1985; vern. *qaggetqet*; leaf only eaten. | ANU T141  (field #9, ‘duplicate of field #17, 23’) | I.T9 |
| 179 | Cultivated | Philippines | CESPH08 | Bayninan, Banaue, Ifugao, Luzon. Coll. DEY & HC, 1985; vern. *qaggetqet*; leaf only eaten. | ANU T142  (field #17, ‘duplicate of field #9, 23’) | I.T9 |
| 180 | Cultivated | Philippines | CESPH09 | Bayninan, Banaue, Ifugao, Luzon. Coll. DEY & HC, 1985; inf. #2; vern. *qaggetqet*; leaf only eaten. | ANU T143  (field #23, ‘duplicate of field #9, 17’) | I.T1 |
| 181 | Wild (likely commensal) | Sri Lanka | CESLK01 | Mt Lavenia, Colombo. Coll. VA, 1985; vern. *habarala* (Sinhala; broad generic name, used also for *Alocasia, Xanthosoma*). | ANU T319 | 1.unique |
| 182 | Wild (likely commensal) | Sri Lanka | CESLK02 | Mt Lavenia, Colombo. Coll. VA, 1985; vern. *habarala* (Sinhala; broad generic name, used also for *Alocasia, Xanthosoma*). | ANU T320 | 1.T4 |
| 183 | Wild | Sri Lanka | CESLK03 | Lakeside, Nuwara Eliya, Central prov. Coll. VA, 1985; var. *fontanesii*; 2*n* = 42. | ANU T322 | 1.unique |
| 184 | Wild (likely commensal) | Sri Lanka | CESLK04 | Colombo. Coll. VA, 1985; forms side-corms. | ANU T323 | II.T5 |
| 185 | Wild | Thailand | CESTH01 | Bangkok; via Dodo Creek Research Station, Honiara, Solomon Islands (variety with *Phytopthera colocasiae* resistance; used for breeding program in 1980s). | ANU T345 | III.T10 |
| 186 | Wild | Thailand | CESTH02 | Ban Nong Jhap Tao, Na Chontien, Chonburi prov. Coll. DEY, 17.11.1986. | ANU T352  (field #1) | III.T10 |
| 187 | Wild | Thailand | CESTH03 | Vic. Archaeology Station, Na Chontien, Chonburi prov. Coll. DEY, 17.11.1986; vern. *bon* (= 'wild'). | ANU T353  (field #2) | III.T10 |
| 188 | Wild (commensal) | Thailand | CESTH04 | Sumit Ngam Is., Chanthaburi R., Chanthaburi prov. Coll. DEY 17.11.1986; vern. *bon* (‘wild’); in garden near river. | ANU T356  (field #5) | I.T1 |
| 189 | Cultivated | Thailand | CESTH05 | Sattahip Nursery, Na Chontien, Chonburi prov. Coll. DEY, 17.11.1986; vern. *poek.* | ANU T359  (field #8) | I.T1 |
| 190 | - | Timor Leste | CESTL01 | Via Lyon Arboretum, Honolulu, Hawaii (no number). Coll. DEY, 31.08.1983, orig. coll. Timor, 1963; 2*n* = 42. | ANU T110 | I.T4 |
| 191 | Cultivated | USA | CESHW01 | Hawaii, via Lyon Arboretum, Honolulu. Coll. DEY, 31.08.1983; vern. *ele ele makoko.* | ANU T104  LA L-69-047 | I.T1 |
| 192 | Wild (commensal) | Vietnam | CESVN01 | Edge of pond, in vill. Ba Vi, Hanoi. Coll. PJM & NVD, 16.10.2011; green with long stolons. |  | I.T1 |
| 193 | Wild | Vietnam | CESVN02 | Natural swamp, Lanty, Hu Lien. Coll. PJM & NVD, 18.10.2011; green with long stolons. |  | III.T11 |
| 194 | Wild | Vietnam | CESVN03 | Stream bank, Hu Lien. Coll. PJM & NVD, 19.10.2011; green with long stolons. |  | III.T11 |
| 195 | Wild (commensal) | Vietnam | CESVN04 | Roadside ditch in Lang Thi vill., Hanoi. Coll. PJM & NVD, 20.10.2011; green with long stolons. |  | I.T1 |
| 196 | Wild (commensal) | Vietnam | CESVN05 | Vic. border of Yen Bai prov. Coll. PJM & NVD, 20.10.2011; vern. *khoai mon*, green with long stolons; stolons edible, corms not edible. | WP249 | I.T1 |
| 197 | Wild (commensal) | Vietnam | CESVN06 | Vic. Yen Bai–Lao Cai provincial border. Coll. PJM & NVD, 20.10.2011; vern. *khoai sap*, dark purple with long stolons; stolons and corms eaten. | WP249 | I.T4 |
| 198 | Wild (commensal) | Vietnam | CESVN07 | Môt Túc vill., Luc Yen, Yen Bai prov. Coll. PJM & NVD, 20.10.11; vern. *khoai mon*, green with long stolons; transplanted to bunds of rice pondfield, then spontaneous, flowering & fruiting; used as pig fodder; only young leaf eaten by people. | WP258 | III.T11 |
| 199 | Cultivated | Vietnam | CESVN08 | Môt Túc vill., Luc Yen, Yen Bai prov. Coll. PJM & NVD, 20.10.2011. | WP258 | I.T1 |
| 200 | Cultivated | Vietnam | CESVN09 | Sapa Market, Sapa, Lao Cai prov. Coll. PJM & NK, 21.10.2011; sample a bract from edible stolon sold in market; vern. cv. *co bon* (‘water taro’) (Ray language). |  | I.T1 |
| 201 | Wild (commensal) | Vietnam | CESVN10 | Roadside in vill.; lower Hó Chäu Ba valley, Lai Chau prov. Coll. PJM & NVD, 22.10.2011. | WP291 | III.T11 |
| 202 | Wild | Vietnam | CESVN11 | Sapa–Lao Cai road, Lao Cai prov. Coll. PJM & NVD, 01.08.2012. | WP411 | III.T12 |
| 203 | Wild (commensal) | Vietnam | CESVN12 | Natural river swale planted with corn, downstream from Ba Be (Three Lakes), Bac Can prov. Coll. PJM & NVD, 05.08.2012. | WP425 | I.T1 |
| 204 | Wild (commensal) | Vietnam | CESVN13 | Quang vill., near Thang Hen Lake, Cao Bang prov. Coll. PJM & NVD, 07.08.2012; vern. *co puc*; used as pig fodder. | WP447 | III.T11 |
| 205 | Wild (commensal) | Vietnam | CESVN14 | Ban Lo vill., Loc Binh district, Lang Son prov. Coll. PJM & NVD, 10.08.2012; vern. *bon canh* (Tay language); initially planted as fodder for pigs; petiole & stolon also eaten by people; now spreading in ditch. | WP464 | I.T1 |

**Supporting Table 2. Individual sequences in the Genbank open-access repository.**

Each Genbank accession number represents an individual sequence submitted in one of two series: KF (Ahmed, Matthews, & Lockhart, 2015-2016), and JN (Ahmed, Matthews, et al. 2013b). The .sqn suffix marks each sequence submitted to Genbank in batch data.

| Primer | Sample ID | GenBank  accession number |
| --- | --- | --- |
| ACECP018.sqn | CAFPH01 | KF284166 |
| ACECP018.sqn | CESAU01 | KF284167 |
| ACECP018.sqn | CESAU02 | KF284168 |
| ACECP018.sqn | CESAU03 | KF284169 |
| ACECP018.sqn | CESAU04 | KF284170 |
| ACECP018.sqn | CESAU05 | KF284171 |
| ACECP018.sqn | CESAU06 | KF284172 |
| ACECP018.sqn | CESAU07 | KF284173 |
| ACECP018.sqn | CESAU08 | KF284174 |
| ACECP018.sqn | CESAU09 | KF284175 |
| ACECP018.sqn | CESAU11 | KF284176 |
| ACECP018.sqn | CESAU12 | KF284177 |
| ACECP018.sqn | CESAU13 | KF284178 |
| ACECP018.sqn | CESAU14 | KF284179 |
| ACECP018.sqn | CESAU15 | KF284180 |
| ACECP018.sqn | CESAU16 | KF284181 |
| ACECP018.sqn | CESAU17 | KF284182 |
| ACECP018.sqn | CESAU19 | KF284183 |
| ACECP018.sqn | CESAU20 | KF284184 |
| ACECP018.sqn | CESAU21 | KF284185 |
| ACECP018.sqn | CESAU22 | KF284186 |
| ACECP018.sqn | CESAU23 | KF284187 |
| ACECP018.sqn | CESBD01 | KF284188 |
| ACECP018.sqn | CESEI01 | KF284189 |
| ACECP018.sqn | CESEI02 | KF284190 |
| ACECP018.sqn | CESEI03 | KF284191 |
| ACECP018.sqn | CESET01 | KF284192 |
| ACECP018.sqn | CESHW01 | KF284193 |
| ACECP018.sqn | CESIN01 | KF284194 |
| ACECP018.sqn | CESIN02 | KF284195 |
| ACECP018.sqn | CESIN03 | KF284196 |
| ACECP018.sqn | CESIN06 | KF284197 |
| ACECP018.sqn | CESIN07 | KF284198 |
| ACECP018.sqn | CESIN08 | KF284199 |
| ACECP018.sqn | CESIN09 | KF284200 |
| ACECP018.sqn | CESIN10 | KF284201 |
| ACECP018.sqn | CESIN11 | KF284202 |
| ACECP018.sqn | CESIN12 | KF284203 |
| ACECP018.sqn | CESJP02 | KF284204 |
| ACECP018.sqn | CESJP03 | KF284205 |
| ACECP018.sqn | CESJP04 | KF284206 |
| ACECP018.sqn | CESJP05 | KF284207 |
| ACECP018.sqn | CESJP06 | KF284208 |
| ACECP018.sqn | CESJP07 | KF284209 |
| ACECP018.sqn | CESJP08 | KF284210 |
| ACECP018.sqn | CESJP09 | KF284211 |
| ACECP018.sqn | CESJP10 | KF284212 |
| ACECP018.sqn | CESJP11 | KF284213 |
| ACECP018.sqn | CESJP12 | KF284214 |
| ACECP018.sqn | CESJP13 | KF284215 |
| ACECP018.sqn | CESJP14 | KF284216 |
| ACECP018.sqn | CESJP15 | KF284217 |
| ACECP018.sqn | CESJP16 | KF284218 |
| ACECP018.sqn | CESJP17 | KF284219 |
| ACECP018.sqn | CESJP18 | KF284220 |
| ACECP018.sqn | CESJP19 | KF284221 |
| ACECP018.sqn | CESJP20 | KF284222 |
| ACECP018.sqn | CESJP21 | KF284223 |
| ACECP018.sqn | CESJP22 | KF284224 |
| ACECP018.sqn | CESJP23 | KF284225 |
| ACECP018.sqn | CESJP24 | KF284226 |
| ACECP018.sqn | CESJP25 | KF284227 |
| ACECP018.sqn | CESJP26 | KF284228 |
| ACECP018.sqn | CESJP27 | KF284229 |
| ACECP018.sqn | CESJP28 | KF284230 |
| ACECP018.sqn | CESJP29 | KF284231 |
| ACECP018.sqn | CESLK01 | KF284232 |
| ACECP018.sqn | CESLK02 | KF284233 |
| ACECP018.sqn | CESLK03 | KF284234 |
| ACECP018.sqn | CESLK04 | KF284235 |
| ACECP018.sqn | CESMG01 | KF284236 |
| ACECP018.sqn | CESMG02 | KF284237 |
| ACECP018.sqn | CESMG03 | KF284238 |
| ACECP018.sqn | CESMG04 | KF284239 |
| ACECP018.sqn | CESMM02 | KF284240 |
| ACECP018.sqn | CESMM03 | KF284241 |
| ACECP018.sqn | CESMM06 | KF284242 |
| ACECP018.sqn | CESMM07 | KF284243 |
| ACECP018.sqn | CESMM08 | KF284244 |
| ACECP018.sqn | CESMM09 | KF284245 |
| ACECP018.sqn | CESMM10 | KF284246 |
| ACECP018.sqn | CESMM11 | KF284247 |
| ACECP018.sqn | CESMM12 | KF284248 |
| ACECP018.sqn | CESMM13 | KF284249 |
| ACECP018.sqn | CESNP01 | KF284250 |
| ACECP018.sqn | CESNP02 | KF284251 |
| ACECP018.sqn | CESNP03 | KF284252 |
| ACECP018.sqn | CESNZ01 | KF284253 |
| ACECP018.sqn | CESNZ05 | KF284254 |
| ACECP018.sqn | CESNZ06 | KF284255 |
| ACECP018.sqn | CESNZ07 | KF284256 |
| ACECP018.sqn | CESNZ08 | KF284257 |
| ACECP018.sqn | CESNZ09 | KF284258 |
| ACECP018.sqn | CESNZ10 | KF284259 |
| ACECP018.sqn | CESNZ11 | KF284260 |
| ACECP018.sqn | CESNZ12 | KF284261 |
| ACECP018.sqn | CESNZ13 | KF284262 |
| ACECP018.sqn | CESNZ15 | KF284263 |
| ACECP018.sqn | CESNZ16 | KF284264 |
| ACECP018.sqn | CESNZ17 | KF284265 |
| ACECP018.sqn | CESNZ18 | KF284266 |
| ACECP018.sqn | CESNZ19 | KF284267 |
| ACECP018.sqn | CESNZ20 | KF284268 |
| ACECP018.sqn | CESNZ21 | KF284269 |
| ACECP018.sqn | CESNZ22 | KF284270 |
| ACECP018.sqn | CESNZ23 | KF284271 |
| ACECP018.sqn | CESPG01 | KF284272 |
| ACECP018.sqn | CESPG02 | KF284273 |
| ACECP018.sqn | CESPG03 | KF284274 |
| ACECP018.sqn | CESPG04 | KF284275 |
| ACECP018.sqn | CESPG05 | KF284276 |
| ACECP018.sqn | CESPG06 | KF284277 |
| ACECP018.sqn | CESPG07 | KF284278 |
| ACECP018.sqn | CESPG08 | KF284279 |
| ACECP018.sqn | CESPG09 | KF284280 |
| ACECP018.sqn | CESPG10 | KF284281 |
| ACECP018.sqn | CESPG11 | KF284282 |
| ACECP018.sqn | CESPG12 | KF284283 |
| ACECP018.sqn | CESPG13 | KF284284 |
| ACECP018.sqn | CESPG14 | KF284285 |
| ACECP018.sqn | CESPG15 | KF284286 |
| ACECP018.sqn | CESPG16 | KF284287 |
| ACECP018.sqn | CESPG17 | KF284288 |
| ACECP018.sqn | CESPG18 | KF284289 |
| ACECP018.sqn | CESPG19 | KF284290 |
| ACECP018.sqn | CESPH01 | KF284291 |
| ACECP018.sqn | CESPH02 | KF284292 |
| ACECP018.sqn | CESPH03 | KF284293 |
| ACECP018.sqn | CESPH04 | KF284294 |
| ACECP018.sqn | CESPH05 | KF284295 |
| ACECP018.sqn | CESPH06 | KF284296 |
| ACECP018.sqn | CESPH07 | KF284297 |
| ACECP018.sqn | CESPH08 | KF284298 |
| ACECP018.sqn | CESPH09 | KF284299 |
| ACECP018.sqn | CESPK01 | KF284300 |
| ACECP018.sqn | CESPK02 | KF284301 |
| ACECP018.sqn | CESPK05 | KF284302 |
| ACECP018.sqn | CESPK06 | KF284303 |
| ACECP018.sqn | CESPK07 | KF284304 |
| ACECP018.sqn | CESSI01 | KF284305 |
| ACECP018.sqn | CESSI02 | KF284306 |
| ACECP018.sqn | CESSI03 | KF284307 |
| ACECP018.sqn | CESTH01 | KF284308 |
| ACECP018.sqn | CESTH02 | KF284309 |
| ACECP018.sqn | CESTH03 | KF284310 |
| ACECP018.sqn | CESTH04 | KF284311 |
| ACECP018.sqn | CESTH05 | KF284312 |
| ACECP018.sqn | CESTL01 | KF284313 |
| ACECP018.sqn | CESVN01 | KF284314 |
| ACECP018.sqn | CESVN02 | KF284315 |
| ACECP018.sqn | CESVN03 | KF284316 |
| ACECP018.sqn | CESVN04 | KF284317 |
| ACECP018.sqn | CESVN05 | KF284318 |
| ACECP018.sqn | CESVN06 | KF284319 |
| ACECP018.sqn | CESVN07 | KF284320 |
| ACECP018.sqn | CESVN08 | KF284321 |
| ACECP018.sqn | CESVN09 | KF284322 |
| ACECP018.sqn | CESVN10 | KF284323 |
| ACECP018.sqn | CESVN11 | KF284324 |
| ACECP018.sqn | CESVN12 | KF284325 |
| ACECP018.sqn | CESVN13 | KF284326 |
| ACECP018.sqn | CESVN14 | KF284327 |
| ACECP018.sqn | CFAIN01 | KF284328 |
| ACECP018.sqn | CFOPH01 | KF284330 |
| ACECP018.sqn | CFOTW02 | KF284331 |
| ACECP018.sqn | CLIVN01 | KF284335 |
| ACECP018.sqn | CLIVN02 | KF284336 |
| ACECP018.sqn | CLIVN03 | KF284337 |
| ACECP018.sqn | CMEVN01 | KF284338 |
| ACECP018.sqn | CMEVN02 | KF284339 |
| ACECP018.sqn | CMEVN03 | KF284340 |
| ACECP018.sqn | CSPCY01 | KF284341 |
| ACECP018.sqn | CSPFJ01 | KF284342 |
| ACECP018.sqn | CSPFJ02 | KF284343 |
| ACECP018.sqn | CSPFJ03 | KF284344 |
| ACECP018.sqn | CSPFJ05 | KF284346 |
| ACECP018.sqn | CSPIN01 | KF284348 |
| ACECP018.sqn | CSPMM01 | KF284349 |
| ACECP018.sqn | CSPMM02 | KF284350 |
| ACECP018.sqn | CSPVN01 | KF284351 |
| ACECP018.sqn | CSPVN02 | KF284352 |
| ACECP018.sqn | CSPVN03 | KF284353 |
| ACECP018.sqn | CSPVN04 | KF284354 |
| ACECP018.sqn | CSPVN05 | KF284355 |
| ACECP018.sqn | CYUVN01 | KF284356 |
| ACECP018.sqn | CYUVN02 | KF284357 |
| ACECP018.sqn | CYUVN03 | KF284358 |
| ACECP018.sqn | CYUVN04 | KF284359 |
| ACECP018.sqn | CYUVN05 | KF284360 |
| ACECP018.sqn | RSPMM01 | KF284361 |
| ACECP018.sqn | RSPVN01 | KF284362 |
| ACECP018.sqn | SSPVN01 | KF284363 |
| ACECP026.sqn | CAFPH01 | KF284371 |
| ACECP026.sqn | CESAU01 | KF284372 |
| ACECP026.sqn | CESAU02 | KF284373 |
| ACECP026.sqn | CESAU03 | KF284374 |
| ACECP026.sqn | CESAU04 | KF284375 |
| ACECP026.sqn | CESAU05 | KF284376 |
| ACECP026.sqn | CESAU06 | KF284377 |
| ACECP026.sqn | CESAU07 | KF284378 |
| ACECP026.sqn | CESAU08 | KF284379 |
| ACECP026.sqn | CESAU09 | KF284380 |
| ACECP026.sqn | CESAU11 | KF284381 |
| ACECP026.sqn | CESAU12 | KF284382 |
| ACECP026.sqn | CESAU13 | KF284383 |
| ACECP026.sqn | CESAU14 | KF284384 |
| ACECP026.sqn | CESAU15 | KF284385 |
| ACECP026.sqn | CESAU16 | KF284386 |
| ACECP026.sqn | CESAU17 | KF284387 |
| ACECP026.sqn | CESAU19 | KF284388 |
| ACECP026.sqn | CESAU20 | KF284389 |
| ACECP026.sqn | CESAU21 | KF284390 |
| ACECP026.sqn | CESAU22 | KF284391 |
| ACECP026.sqn | CESAU23 | KF284392 |
| ACECP026.sqn | CESBD01 | KF284393 |
| ACECP026.sqn | CESEI01 | KF284394 |
| ACECP026.sqn | CESEI02 | KF284395 |
| ACECP026.sqn | CESEI03 | KF284396 |
| ACECP026.sqn | CESET01 | KF284397 |
| ACECP026.sqn | CESHW01 | KF284398 |
| ACECP026.sqn | CESIN01 | KF284399 |
| ACECP026.sqn | CESIN02 | KF284400 |
| ACECP026.sqn | CESIN03 | KF284401 |
| ACECP026.sqn | CESIN06 | KF284402 |
| ACECP026.sqn | CESIN07 | KF284403 |
| ACECP026.sqn | CESIN08 | KF284404 |
| ACECP026.sqn | CESIN09 | KF284405 |
| ACECP026.sqn | CESIN10 | KF284406 |
| ACECP026.sqn | CESIN11 | KF284407 |
| ACECP026.sqn | CESIN12 | KF284408 |
| ACECP026.sqn | CESJP02 | KF284409 |
| ACECP026.sqn | CESJP03 | KF284410 |
| ACECP026.sqn | CESJP04 | KF284411 |
| ACECP026.sqn | CESJP05 | KF284412 |
| ACECP026.sqn | CESJP06 | KF284413 |
| ACECP026.sqn | CESJP07 | KF284414 |
| ACECP026.sqn | CESJP08 | KF284415 |
| ACECP026.sqn | CESJP09 | KF284416 |
| ACECP026.sqn | CESJP10 | KF284417 |
| ACECP026.sqn | CESJP11 | KF284418 |
| ACECP026.sqn | CESJP12 | KF284419 |
| ACECP026.sqn | CESJP13 | KF284420 |
| ACECP026.sqn | CESJP14 | KF284421 |
| ACECP026.sqn | CESJP15 | KF284422 |
| ACECP026.sqn | CESJP16 | KF284423 |
| ACECP026.sqn | CESJP17 | KF284424 |
| ACECP026.sqn | CESJP18 | KF284425 |
| ACECP026.sqn | CESJP19 | KF284426 |
| ACECP026.sqn | CESJP20 | KF284427 |
| ACECP026.sqn | CESJP21 | KF284428 |
| ACECP026.sqn | CESJP22 | KF284429 |
| ACECP026.sqn | CESJP23 | KF284430 |
| ACECP026.sqn | CESJP24 | KF284431 |
| ACECP026.sqn | CESJP25 | KF284432 |
| ACECP026.sqn | CESJP26 | KF284433 |
| ACECP026.sqn | CESJP27 | KF284434 |
| ACECP026.sqn | CESJP28 | KF284435 |
| ACECP026.sqn | CESJP29 | KF284436 |
| ACECP026.sqn | CESLK01 | KF284437 |
| ACECP026.sqn | CESLK02 | KF284438 |
| ACECP026.sqn | CESLK03 | KF284439 |
| ACECP026.sqn | CESLK04 | KF284440 |
| ACECP026.sqn | CESMG01 | KF284441 |
| ACECP026.sqn | CESMG02 | KF284442 |
| ACECP026.sqn | CESMG03 | KF284443 |
| ACECP026.sqn | CESMG04 | KF284444 |
| ACECP026.sqn | CESMM02 | KF284445 |
| ACECP026.sqn | CESMM03 | KF284446 |
| ACECP026.sqn | CESMM06 | KF284447 |
| ACECP026.sqn | CESMM07 | KF284448 |
| ACECP026.sqn | CESMM08 | KF284449 |
| ACECP026.sqn | CESMM09 | KF284450 |
| ACECP026.sqn | CESMM10 | KF284451 |
| ACECP026.sqn | CESMM11 | KF284452 |
| ACECP026.sqn | CESMM12 | KF284453 |
| ACECP026.sqn | CESMM13 | KF284454 |
| ACECP026.sqn | CESNP01 | KF284455 |
| ACECP026.sqn | CESNP02 | KF284456 |
| ACECP026.sqn | CESNP03 | KF284457 |
| ACECP026.sqn | CESNZ04 | KF284458 |
| ACECP026.sqn | CESNZ05 | KF284459 |
| ACECP026.sqn | CESNZ06 | KF284460 |
| ACECP026.sqn | CESNZ07 | KF284461 |
| ACECP026.sqn | CESNZ08 | KF284462 |
| ACECP026.sqn | CESNZ09 | KF284463 |
| ACECP026.sqn | CESNZ10 | KF284464 |
| ACECP026.sqn | CESNZ11 | KF284465 |
| ACECP026.sqn | CESNZ12 | KF284466 |
| ACECP026.sqn | CESNZ13 | KF284467 |
| ACECP026.sqn | CESNZ15 | KF284468 |
| ACECP026.sqn | CESNZ16 | KF284469 |
| ACECP026.sqn | CESNZ17 | KF284470 |
| ACECP026.sqn | CESNZ18 | KF284471 |
| ACECP026.sqn | CESNZ19 | KF284472 |
| ACECP026.sqn | CESNZ20 | KF284473 |
| ACECP026.sqn | CESNZ21 | KF284474 |
| ACECP026.sqn | CESNZ22 | KF284475 |
| ACECP026.sqn | CESNZ23 | KF284476 |
| ACECP026.sqn | CESPG01 | KF284477 |
| ACECP026.sqn | CESPG02 | KF284478 |
| ACECP026.sqn | CESPG03 | KF284479 |
| ACECP026.sqn | CESPG04 | KF284480 |
| ACECP026.sqn | CESPG05 | KF284481 |
| ACECP026.sqn | CESPG06 | KF284482 |
| ACECP026.sqn | CESPG07 | KF284483 |
| ACECP026.sqn | CESPG08 | KF284484 |
| ACECP026.sqn | CESPG09 | KF284485 |
| ACECP026.sqn | CESPG10 | KF284486 |
| ACECP026.sqn | CESPG11 | KF284487 |
| ACECP026.sqn | CESPG12 | KF284488 |
| ACECP026.sqn | CESPG13 | KF284489 |
| ACECP026.sqn | CESPG14 | KF284490 |
| ACECP026.sqn | CESPG15 | KF284491 |
| ACECP026.sqn | CESPG16 | KF284492 |
| ACECP026.sqn | CESPG17 | KF284493 |
| ACECP026.sqn | CESPG18 | KF284494 |
| ACECP026.sqn | CESPG19 | KF284495 |
| ACECP026.sqn | CESPH01 | KF284496 |
| ACECP026.sqn | CESPH02 | KF284497 |
| ACECP026.sqn | CESPH03 | KF284498 |
| ACECP026.sqn | CESPH04 | KF284499 |
| ACECP026.sqn | CESPH05 | KF284500 |
| ACECP026.sqn | CESPH06 | KF284501 |
| ACECP026.sqn | CESPH07 | KF284502 |
| ACECP026.sqn | CESPH08 | KF284503 |
| ACECP026.sqn | CESPH09 | KF284504 |
| ACECP026.sqn | CESPK01 | KF284505 |
| ACECP026.sqn | CESPK02 | KF284506 |
| ACECP026.sqn | CESPK05 | KF284507 |
| ACECP026.sqn | CESPK06 | KF284508 |
| ACECP026.sqn | CESPK07 | KF284509 |
| ACECP026.sqn | CESSI01 | KF284510 |
| ACECP026.sqn | CESSI02 | KF284511 |
| ACECP026.sqn | CESSI03 | KF284512 |
| ACECP026.sqn | CESTH01 | KF284513 |
| ACECP026.sqn | CESTH02 | KF284514 |
| ACECP026.sqn | CESTH03 | KF284515 |
| ACECP026.sqn | CESTH04 | KF284516 |
| ACECP026.sqn | CESTH05 | KF284517 |
| ACECP026.sqn | CESTL01 | KF284518 |
| ACECP026.sqn | CESVN01 | KF284519 |
| ACECP026.sqn | CESVN02 | KF284520 |
| ACECP026.sqn | CESVN03 | KF284521 |
| ACECP026.sqn | CESVN04 | KF284522 |
| ACECP026.sqn | CESVN05 | KF284523 |
| ACECP026.sqn | CESVN06 | KF284524 |
| ACECP026.sqn | CESVN07 | KF284525 |
| ACECP026.sqn | CESVN08 | KF284526 |
| ACECP026.sqn | CESVN09 | KF284527 |
| ACECP026.sqn | CESVN10 | KF284528 |
| ACECP026.sqn | CESVN11 | KF284529 |
| ACECP026.sqn | CESVN12 | KF284530 |
| ACECP026.sqn | CESVN13 | KF284531 |
| ACECP026.sqn | CESVN14 | KF284532 |
| ACECP026.sqn | CFAIN01 | KF284533 |
| ACECP026.sqn | CFOPH01 | KF284535 |
| ACECP026.sqn | CFOTW02 | KF284536 |
| ACECP026.sqn | CLIVN01 | KF284540 |
| ACECP026.sqn | CLIVN02 | KF284541 |
| ACECP026.sqn | CLIVN03 | KF284542 |
| ACECP026.sqn | CMEVN01 | KF284543 |
| ACECP026.sqn | CMEVN02 | KF284544 |
| ACECP026.sqn | CMEVN03 | KF284545 |
| ACECP026.sqn | CSPCY01 | KF284546 |
| ACECP026.sqn | CSPFJ01 | KF284547 |
| ACECP026.sqn | CSPFJ02 | KF284548 |
| ACECP026.sqn | CSPFJ03 | KF284549 |
| ACECP026.sqn | CSPFJ05 | KF284551 |
| ACECP026.sqn | CSPIN01 | KF284553 |
| ACECP026.sqn | CSPMM01 | KF284554 |
| ACECP026.sqn | CSPMM02 | KF284555 |
| ACECP026.sqn | CSPVN01 | KF284556 |
| ACECP026.sqn | CSPVN02 | KF284557 |
| ACECP026.sqn | CSPVN03 | KF284558 |
| ACECP026.sqn | CSPVN04 | KF284559 |
| ACECP026.sqn | CSPVN05 | KF284560 |
| ACECP026.sqn | CYUVN01 | KF284561 |
| ACECP026.sqn | CYUVN02 | KF284562 |
| ACECP026.sqn | CYUVN03 | KF284563 |
| ACECP026.sqn | CYUVN04 | KF284564 |
| ACECP026.sqn | CYUVN05 | KF284565 |
| ACECP026.sqn | RSPMM01 | KF284566 |
| ACECP026.sqn | RSPVN01 | KF284567 |
| ACECP026.sqn | SSPVN01 | KF284568 |
| ACECP039.sqn | CAFPH01 | KF284576 |
| ACECP039.sqn | CESAU01 | KF284577 |
| ACECP039.sqn | CESAU02 | KF284578 |
| ACECP039.sqn | CESAU03 | KF284579 |
| ACECP039.sqn | CESAU04 | KF284580 |
| ACECP039.sqn | CESAU05 | KF284581 |
| ACECP039.sqn | CESAU06 | KF284582 |
| ACECP039.sqn | CESAU07 | KF284583 |
| ACECP039.sqn | CESAU08 | KF284584 |
| ACECP039.sqn | CESAU09 | KF284585 |
| ACECP039.sqn | CESAU11 | KF284586 |
| ACECP039.sqn | CESAU12 | KF284587 |
| ACECP039.sqn | CESAU13 | KF284588 |
| ACECP039.sqn | CESAU14 | KF284589 |
| ACECP039.sqn | CESAU15 | KF284590 |
| ACECP039.sqn | CESAU16 | KF284591 |
| ACECP039.sqn | CESAU17 | KF284592 |
| ACECP039.sqn | CESAU19 | KF284593 |
| ACECP039.sqn | CESAU20 | KF284594 |
| ACECP039.sqn | CESAU21 | KF284595 |
| ACECP039.sqn | CESAU22 | KF284596 |
| ACECP039.sqn | CESAU23 | KF284597 |
| ACECP039.sqn | CESBD01 | KF284598 |
| ACECP039.sqn | CESEI01 | KF284599 |
| ACECP039.sqn | CESEI02 | KF284600 |
| ACECP039.sqn | CESEI03 | KF284601 |
| ACECP039.sqn | CESET01 | KF284602 |
| ACECP039.sqn | CESHW01 | KF284603 |
| ACECP039.sqn | CESIN01 | KF284604 |
| ACECP039.sqn | CESIN02 | KF284605 |
| ACECP039.sqn | CESIN03 | KF284606 |
| ACECP039.sqn | CESIN06 | KF284607 |
| ACECP039.sqn | CESIN07 | KF284608 |
| ACECP039.sqn | CESIN08 | KF284609 |
| ACECP039.sqn | CESIN10 | KF284610 |
| ACECP039.sqn | CESIN11 | KF284611 |
| ACECP039.sqn | CESIN12 | KF284612 |
| ACECP039.sqn | CESJP02 | KF284613 |
| ACECP039.sqn | CESJP03 | KF284614 |
| ACECP039.sqn | CESJP04 | KF284615 |
| ACECP039.sqn | CESJP05 | KF284616 |
| ACECP039.sqn | CESJP06 | KF284617 |
| ACECP039.sqn | CESJP08 | KF284618 |
| ACECP039.sqn | CESJP09 | KF284619 |
| ACECP039.sqn | CESJP10 | KF284620 |
| ACECP039.sqn | CESJP11 | KF284621 |
| ACECP039.sqn | CESJP12 | KF284622 |
| ACECP039.sqn | CESJP13 | KF284623 |
| ACECP039.sqn | CESJP14 | KF284624 |
| ACECP039.sqn | CESJP15 | KF284625 |
| ACECP039.sqn | CESJP16 | KF284626 |
| ACECP039.sqn | CESJP17 | KF284627 |
| ACECP039.sqn | CESJP18 | KF284628 |
| ACECP039.sqn | CESJP19 | KF284629 |
| ACECP039.sqn | CESJP20 | KF284630 |
| ACECP039.sqn | CESJP21 | KF284631 |
| ACECP039.sqn | CESJP22 | KF284632 |
| ACECP039.sqn | CESJP23 | KF284633 |
| ACECP039.sqn | CESJP24 | KF284634 |
| ACECP039.sqn | CESJP25 | KF284635 |
| ACECP039.sqn | CESJP26 | KF284636 |
| ACECP039.sqn | CESJP27 | KF284637 |
| ACECP039.sqn | CESJP28 | KF284638 |
| ACECP039.sqn | CESJP29 | KF284639 |
| ACECP039.sqn | CESLK01 | KF284640 |
| ACECP039.sqn | CESLK02 | KF284641 |
| ACECP039.sqn | CESLK03 | KF284642 |
| ACECP039.sqn | CESLK04 | KF284643 |
| ACECP039.sqn | CESMG01 | KF284644 |
| ACECP039.sqn | CESMG02 | KF284645 |
| ACECP039.sqn | CESMG03 | KF284646 |
| ACECP039.sqn | CESMG04 | KF284647 |
| ACECP039.sqn | CESMM02 | KF284648 |
| ACECP039.sqn | CESMM03 | KF284649 |
| ACECP039.sqn | CESMM07 | KF284650 |
| ACECP039.sqn | CESMM08 | KF284651 |
| ACECP039.sqn | CESMM09 | KF284652 |
| ACECP039.sqn | CESMM10 | KF284653 |
| ACECP039.sqn | CESMM12 | KF284654 |
| ACECP039.sqn | CESMM13 | KF284655 |
| ACECP039.sqn | CESNP01 | KF284656 |
| ACECP039.sqn | CESNP02 | KF284657 |
| ACECP039.sqn | CESNP03 | KF284658 |
| ACECP039.sqn | CESNZ04 | KF284659 |
| ACECP039.sqn | CESNZ05 | KF284660 |
| ACECP039.sqn | CESNZ06 | KF284661 |
| ACECP039.sqn | CESNZ07 | KF284662 |
| ACECP039.sqn | CESNZ08 | KF284663 |
| ACECP039.sqn | CESNZ09 | KF284664 |
| ACECP039.sqn | CESNZ10 | KF284665 |
| ACECP039.sqn | CESNZ11 | KF284666 |
| ACECP039.sqn | CESNZ12 | KF284667 |
| ACECP039.sqn | CESNZ13 | KF284668 |
| ACECP039.sqn | CESNZ15 | KF284669 |
| ACECP039.sqn | CESNZ16 | KF284670 |
| ACECP039.sqn | CESNZ17 | KF284671 |
| ACECP039.sqn | CESNZ18 | KF284672 |
| ACECP039.sqn | CESNZ19 | KF284673 |
| ACECP039.sqn | CESNZ20 | KF284674 |
| ACECP039.sqn | CESNZ21 | KF284675 |
| ACECP039.sqn | CESNZ22 | KF284676 |
| ACECP039.sqn | CESNZ23 | KF284677 |
| ACECP039.sqn | CESPG01 | KF284678 |
| ACECP039.sqn | CESPG02 | KF284679 |
| ACECP039.sqn | CESPG03 | KF284680 |
| ACECP039.sqn | CESPG04 | KF284681 |
| ACECP039.sqn | CESPG05 | KF284682 |
| ACECP039.sqn | CESPG06 | KF284683 |
| ACECP039.sqn | CESPG07 | KF284684 |
| ACECP039.sqn | CESPG08 | KF284685 |
| ACECP039.sqn | CESPG09 | KF284686 |
| ACECP039.sqn | CESPG10 | KF284687 |
| ACECP039.sqn | CESPG11 | KF284688 |
| ACECP039.sqn | CESPG12 | KF284689 |
| ACECP039.sqn | CESPG13 | KF284690 |
| ACECP039.sqn | CESPG14 | KF284691 |
| ACECP039.sqn | CESPG15 | KF284692 |
| ACECP039.sqn | CESPG16 | KF284693 |
| ACECP039.sqn | CESPG17 | KF284694 |
| ACECP039.sqn | CESPG18 | KF284695 |
| ACECP039.sqn | CESPG19 | KF284696 |
| ACECP039.sqn | CESPH01 | KF284697 |
| ACECP039.sqn | CESPH02 | KF284698 |
| ACECP039.sqn | CESPH03 | KF284699 |
| ACECP039.sqn | CESPH04 | KF284700 |
| ACECP039.sqn | CESPH05 | KF284701 |
| ACECP039.sqn | CESPH06 | KF284702 |
| ACECP039.sqn | CESPH07 | KF284703 |
| ACECP039.sqn | CESPH08 | KF284704 |
| ACECP039.sqn | CESPH09 | KF284705 |
| ACECP039.sqn | CESPK01 | KF284706 |
| ACECP039.sqn | CESPK02 | KF284707 |
| ACECP039.sqn | CESPK05 | KF284708 |
| ACECP039.sqn | CESPK06 | KF284709 |
| ACECP039.sqn | CESPK07 | KF284710 |
| ACECP039.sqn | CESSI01 | KF284711 |
| ACECP039.sqn | CESSI02 | KF284712 |
| ACECP039.sqn | CESSI03 | KF284713 |
| ACECP039.sqn | CESTH01 | KF284714 |
| ACECP039.sqn | CESTH02 | KF284715 |
| ACECP039.sqn | CESTH03 | KF284716 |
| ACECP039.sqn | CESTH04 | KF284717 |
| ACECP039.sqn | CESTH05 | KF284718 |
| ACECP039.sqn | CESTL01 | KF284719 |
| ACECP039.sqn | CESVN01 | KF284720 |
| ACECP039.sqn | CESVN02 | KF284721 |
| ACECP039.sqn | CESVN03 | KF284722 |
| ACECP039.sqn | CESVN04 | KF284723 |
| ACECP039.sqn | CESVN05 | KF284724 |
| ACECP039.sqn | CESVN06 | KF284725 |
| ACECP039.sqn | CESVN07 | KF284726 |
| ACECP039.sqn | CESVN08 | KF284727 |
| ACECP039.sqn | CESVN09 | KF284728 |
| ACECP039.sqn | CESVN10 | KF284729 |
| ACECP039.sqn | CESVN11 | KF284730 |
| ACECP039.sqn | CESVN12 | KF284731 |
| ACECP039.sqn | CESVN13 | KF284732 |
| ACECP039.sqn | CESVN14 | KF284733 |
| ACECP039.sqn | CFAIN01 | KF284734 |
| ACECP039.sqn | CFOPH01 | KF284737 |
| ACECP039.sqn | CFOTW02 | KF284738 |
| ACECP039.sqn | CLIVN01 | KF284742 |
| ACECP039.sqn | CLIVN02 | KF284743 |
| ACECP039.sqn | CLIVN03 | KF284744 |
| ACECP039.sqn | CMEVN01 | KF284745 |
| ACECP039.sqn | CMEVN02 | KF284746 |
| ACECP039.sqn | CMEVN03 | KF284747 |
| ACECP039.sqn | CSPCY01 | KF284748 |
| ACECP039.sqn | CSPFJ01 | KF284749 |
| ACECP039.sqn | CSPFJ02 | KF284750 |
| ACECP039.sqn | CSPFJ03 | KF284751 |
| ACECP039.sqn | CSPFJ05 | KF284753 |
| ACECP039.sqn | CSPIN01 | KF284754 |
| ACECP039.sqn | CSPMM01 | KF284755 |
| ACECP039.sqn | CSPMM02 | KF284756 |
| ACECP039.sqn | CSPVN01 | KF284757 |
| ACECP039.sqn | CSPVN02 | KF284758 |
| ACECP039.sqn | CSPVN03 | KF284759 |
| ACECP039.sqn | CSPVN04 | KF284760 |
| ACECP039.sqn | CSPVN05 | KF284761 |
| ACECP039.sqn | CYUVN01 | KF284762 |
| ACECP039.sqn | CYUVN02 | KF284763 |
| ACECP039.sqn | CYUVN03 | KF284764 |
| ACECP039.sqn | CYUVN04 | KF284765 |
| ACECP039.sqn | CYUVN05 | KF284766 |
| ACECP039.sqn | RSPMM01 | KF284767 |
| ACECP039.sqn | RSPVN01 | KF284768 |
| ACECP039.sqn | SSPVN01 | KF284769 |
| ACECP005.sqn | CAFPH01 | KF284855 |
| ACECP005.sqn | CESAU01 | KF284856 |
| ACECP005.sqn | CESAU02 | KF284857 |
| ACECP005.sqn | CESAU03 | KF284858 |
| ACECP005.sqn | CESAU04 | KF284859 |
| ACECP005.sqn | CESAU05 | KF284860 |
| ACECP005.sqn | CESAU06 | KF284861 |
| ACECP005.sqn | CESAU07 | KF284862 |
| ACECP005.sqn | CESAU08 | KF284863 |
| ACECP005.sqn | CESAU09 | KF284864 |
| ACECP005.sqn | CESAU11 | KF284865 |
| ACECP005.sqn | CESAU12 | KF284866 |
| ACECP005.sqn | CESAU13 | KF284867 |
| ACECP005.sqn | CESAU14 | KF284868 |
| ACECP005.sqn | CESAU15 | KF284869 |
| ACECP005.sqn | CESAU16 | KF284870 |
| ACECP005.sqn | CESAU17 | KF284871 |
| ACECP005.sqn | CESAU19 | KF284872 |
| ACECP005.sqn | CESAU20 | KF284873 |
| ACECP005.sqn | CESAU21 | KF284874 |
| ACECP005.sqn | CESAU22 | KF284875 |
| ACECP005.sqn | CESAU23 | KF284876 |
| ACECP005.sqn | CESBD01 | KF284877 |
| ACECP005.sqn | CESEI01 | KF284878 |
| ACECP005.sqn | CESEI02 | KF284879 |
| ACECP005.sqn | CESEI03 | KF284880 |
| ACECP005.sqn | CESET01 | KF284881 |
| ACECP005.sqn | CESHW01 | KF284882 |
| ACECP005.sqn | CESIN01 | KF284883 |
| ACECP005.sqn | CESIN02 | KF284884 |
| ACECP005.sqn | CESIN03 | KF284885 |
| ACECP005.sqn | CESIN06 | KF284886 |
| ACECP005.sqn | CESIN07 | KF284887 |
| ACECP005.sqn | CESIN08 | KF284888 |
| ACECP005.sqn | CESIN09 | KF284889 |
| ACECP005.sqn | CESIN10 | KF284890 |
| ACECP005.sqn | CESIN11 | KF284891 |
| ACECP005.sqn | CESIN12 | KF284892 |
| ACECP005.sqn | CESJP02 | KF284893 |
| ACECP005.sqn | CESJP03 | KF284894 |
| ACECP005.sqn | CESJP04 | KF284895 |
| ACECP005.sqn | CESJP05 | KF284896 |
| ACECP005.sqn | CESJP06 | KF284897 |
| ACECP005.sqn | CESJP07 | KF284898 |
| ACECP005.sqn | CESJP08 | KF284899 |
| ACECP005.sqn | CESJP09 | KF284900 |
| ACECP005.sqn | CESJP10 | KF284901 |
| ACECP005.sqn | CESJP11 | KF284902 |
| ACECP005.sqn | CESJP12 | KF284903 |
| ACECP005.sqn | CESJP13 | KF284904 |
| ACECP005.sqn | CESJP14 | KF284905 |
| ACECP005.sqn | CESJP15 | KF284906 |
| ACECP005.sqn | CESJP16 | KF284907 |
| ACECP005.sqn | CESJP17 | KF284908 |
| ACECP005.sqn | CESJP18 | KF284909 |
| ACECP005.sqn | CESJP19 | KF284910 |
| ACECP005.sqn | CESJP20 | KF284911 |
| ACECP005.sqn | CESJP21 | KF284912 |
| ACECP005.sqn | CESJP22 | KF284913 |
| ACECP005.sqn | CESJP23 | KF284914 |
| ACECP005.sqn | CESJP24 | KF284915 |
| ACECP005.sqn | CESJP25 | KF284916 |
| ACECP005.sqn | CESJP26 | KF284917 |
| ACECP005.sqn | CESJP27 | KF284918 |
| ACECP005.sqn | CESJP28 | KF284919 |
| ACECP005.sqn | CESJP29 | KF284920 |
| ACECP005.sqn | CESLK01 | KF284921 |
| ACECP005.sqn | CESLK02 | KF284922 |
| ACECP005.sqn | CESLK03 | KF284923 |
| ACECP005.sqn | CESLK04 | KF284924 |
| ACECP005.sqn | CESMG01 | KF284925 |
| ACECP005.sqn | CESMG02 | KF284926 |
| ACECP005.sqn | CESMG03 | KF284927 |
| ACECP005.sqn | CESMG04 | KF284928 |
| ACECP005.sqn | CESMM02 | KF284929 |
| ACECP005.sqn | CESMM03 | KF284930 |
| ACECP005.sqn | CESMM06 | KF284931 |
| ACECP005.sqn | CESMM07 | KF284932 |
| ACECP005.sqn | CESMM08 | KF284933 |
| ACECP005.sqn | CESMM09 | KF284934 |
| ACECP005.sqn | CESMM10 | KF284935 |
| ACECP005.sqn | CESMM11 | KF284936 |
| ACECP005.sqn | CESMM13 | KF284937 |
| ACECP005.sqn | CESNP01 | KF284938 |
| ACECP005.sqn | CESNP02 | KF284939 |
| ACECP005.sqn | CESNP03 | KF284940 |
| ACECP005.sqn | CESNZ04 | KF284941 |
| ACECP005.sqn | CESNZ05 | KF284942 |
| ACECP005.sqn | CESNZ06 | KF284943 |
| ACECP005.sqn | CESNZ07 | KF284944 |
| ACECP005.sqn | CESNZ08 | KF284945 |
| ACECP005.sqn | CESNZ09 | KF284946 |
| ACECP005.sqn | CESNZ10 | KF284947 |
| ACECP005.sqn | CESNZ11 | KF284948 |
| ACECP005.sqn | CESNZ12 | KF284949 |
| ACECP005.sqn | CESNZ13 | KF284950 |
| ACECP005.sqn | CESNZ15 | KF284951 |
| ACECP005.sqn | CESNZ16 | KF284952 |
| ACECP005.sqn | CESNZ17 | KF284953 |
| ACECP005.sqn | CESNZ18 | KF284954 |
| ACECP005.sqn | CESNZ19 | KF284955 |
| ACECP005.sqn | CESNZ20 | KF284956 |
| ACECP005.sqn | CESNZ21 | KF284957 |
| ACECP005.sqn | CESNZ22 | KF284958 |
| ACECP005.sqn | CESNZ23 | KF284959 |
| ACECP005.sqn | CESPG01 | KF284960 |
| ACECP005.sqn | CESPG02 | KF284961 |
| ACECP005.sqn | CESPG03 | KF284962 |
| ACECP005.sqn | CESPG04 | KF284963 |
| ACECP005.sqn | CESPG05 | KF284964 |
| ACECP005.sqn | CESPG06 | KF284965 |
| ACECP005.sqn | CESPG07 | KF284966 |
| ACECP005.sqn | CESPG08 | KF284967 |
| ACECP005.sqn | CESPG09 | KF284968 |
| ACECP005.sqn | CESPG10 | KF284969 |
| ACECP005.sqn | CESPG11 | KF284970 |
| ACECP005.sqn | CESPG12 | KF284971 |
| ACECP005.sqn | CESPG13 | KF284972 |
| ACECP005.sqn | CESPG14 | KF284973 |
| ACECP005.sqn | CESPG15 | KF284974 |
| ACECP005.sqn | CESPG16 | KF284975 |
| ACECP005.sqn | CESPG17 | KF284976 |
| ACECP005.sqn | CESPG18 | KF284977 |
| ACECP005.sqn | CESPG19 | KF284978 |
| ACECP005.sqn | CESPH01 | KF284979 |
| ACECP005.sqn | CESPH02 | KF284980 |
| ACECP005.sqn | CESPH03 | KF284981 |
| ACECP005.sqn | CESPH04 | KF284982 |
| ACECP005.sqn | CESPH05 | KF284983 |
| ACECP005.sqn | CESPH06 | KF284984 |
| ACECP005.sqn | CESPH07 | KF284985 |
| ACECP005.sqn | CESPH08 | KF284986 |
| ACECP005.sqn | CESPH09 | KF284987 |
| ACECP005.sqn | CESPK01 | KF284988 |
| ACECP005.sqn | CESPK02 | KF284989 |
| ACECP005.sqn | CESPK05 | KF284990 |
| ACECP005.sqn | CESPK06 | KF284991 |
| ACECP005.sqn | CESPK07 | KF284992 |
| ACECP005.sqn | CESSI01 | KF284993 |
| ACECP005.sqn | CESSI02 | KF284994 |
| ACECP005.sqn | CESSI03 | KF284995 |
| ACECP005.sqn | CESTH01 | KF284996 |
| ACECP005.sqn | CESTH02 | KF284997 |
| ACECP005.sqn | CESTH03 | KF284998 |
| ACECP005.sqn | CESTH04 | KF284999 |
| ACECP005.sqn | CESTH05 | KF285000 |
| ACECP005.sqn | CESTL01 | KF285001 |
| ACECP005.sqn | CESVN01 | KF285002 |
| ACECP005.sqn | CESVN02 | KF285003 |
| ACECP005.sqn | CESVN03 | KF285004 |
| ACECP005.sqn | CESVN04 | KF285005 |
| ACECP005.sqn | CESVN05 | KF285006 |
| ACECP005.sqn | CESVN06 | KF285007 |
| ACECP005.sqn | CESVN07 | KF285008 |
| ACECP005.sqn | CESVN08 | KF285009 |
| ACECP005.sqn | CESVN09 | KF285010 |
| ACECP005.sqn | CESVN10 | KF285011 |
| ACECP005.sqn | CESVN11 | KF285012 |
| ACECP005.sqn | CESVN12 | KF285013 |
| ACECP005.sqn | CESVN13 | KF285014 |
| ACECP005.sqn | CESVN14 | KF285015 |
| ACECP005.sqn | CFAIN01 | KF285016 |
| ACECP005.sqn | CFOPH01 | KF285018 |
| ACECP005.sqn | CFOTW02 | KF285019 |
| ACECP005.sqn | CLIVN01 | KF285020 |
| ACECP005.sqn | CLIVN02 | KF285021 |
| ACECP005.sqn | CLIVN03 | KF285022 |
| ACECP005.sqn | CMEVN01 | KF285023 |
| ACECP005.sqn | CMEVN02 | KF285024 |
| ACECP005.sqn | CMEVN03 | KF285025 |
| ACECP005.sqn | CSPCY01 | KF285026 |
| ACECP005.sqn | CSPFJ01 | KF285027 |
| ACECP005.sqn | CSPFJ02 | KF285028 |
| ACECP005.sqn | CSPFJ03 | KF285029 |
| ACECP005.sqn | CSPFJ05 | KF285030 |
| ACECP005.sqn | CSPIN01 | KF285032 |
| ACECP005.sqn | CSPMM01 | KF285033 |
| ACECP005.sqn | CSPMM02 | KF285034 |
| ACECP005.sqn | CSPVN01 | KF285035 |
| ACECP005.sqn | CSPVN02 | KF285036 |
| ACECP005.sqn | CSPVN03 | KF285037 |
| ACECP005.sqn | CSPVN04 | KF285038 |
| ACECP005.sqn | CSPVN05 | KF285039 |
| ACECP005.sqn | CYUVN01 | KF285040 |
| ACECP005.sqn | CYUVN02 | KF285041 |
| ACECP005.sqn | CYUVN03 | KF285042 |
| ACECP005.sqn | CYUVN04 | KF285043 |
| ACECP005.sqn | CYUVN05 | KF285044 |
| ACECP005.sqn | RSPMM01 | KF285045 |
| ACECP005.sqn | RSPVN01 | KF285046 |
| ACECP005.sqn | SSPVN01 | KF285047 |
| ACECP016.sqn | CESAU03 | KF285049 |
| ACECP016.sqn | CESAU08 | KF285050 |
| ACECP016.sqn | CESAU09 | KF285051 |
| ACECP016.sqn | CESAU22 | KF285052 |
| ACECP016.sqn | CESJP03 | KF285053 |
| ACECP016.sqn | CESJP04 | KF285054 |
| ACECP016.sqn | CESJP06 | KF285055 |
| ACECP016.sqn | CESJP07 | KF285056 |
| ACECP016.sqn | CESJP08 | KF285057 |
| ACECP016.sqn | CESJP12 | KF285058 |
| ACECP016.sqn | CESJP13 | KF285059 |
| ACECP016.sqn | CESMM02 | KF285060 |
| ACECP016.sqn | CESMM10 | KF285061 |
| ACECP016.sqn | CESNP03 | KF285062 |
| ACECP016.sqn | CESNZ05 | KF285063 |
| ACECP016.sqn | CESNZ06 | KF285064 |
| ACECP016.sqn | CESNZ07 | KF285065 |
| ACECP016.sqn | CESNZ09 | KF285066 |
| ACECP016.sqn | CESNZ10 | KF285067 |
| ACECP016.sqn | CESNZ17 | KF285068 |
| ACECP016.sqn | CESNZ19 | KF285069 |
| ACECP016.sqn | CESNZ21 | KF285070 |
| ACECP016.sqn | CESNZ22 | KF285071 |
| ACECP016.sqn | CESNZ23 | KF285072 |
| ACECP016.sqn | CESPG06 | KF285073 |
| ACECP016.sqn | CESVN07 | KF285074 |
| ACECP016.sqn | CESVN11 | KF285075 |
| ACECP016.sqn | CFOPH01 | KF285076 |
| ACECP016.sqn | CFOTW02 | KF285077 |
| ACECP016.sqn | CLIVN03 | KF285081 |
| ACECP016.sqn | CSPVN01 | KF285084 |
| ACECP016.sqn | CSPVN04 | KF285085 |
| ACECP016.sqn | CSPVN05 | KF285086 |
| ACECP016.sqn | CYUVN04 | KF285087 |
| ACECP016.sqn | SSPVN01 | KF285088 |
| ACECP035.sqn | CAFPH01 | KF285090 |
| ACECP035.sqn | CESAU01 | KF285091 |
| ACECP035.sqn | CESAU02 | KF285092 |
| ACECP035.sqn | CESAU03 | KF285093 |
| ACECP035.sqn | CESAU04 | KF285094 |
| ACECP035.sqn | CESAU05 | KF285095 |
| ACECP035.sqn | CESAU08 | KF285096 |
| ACECP035.sqn | CESAU11 | KF285097 |
| ACECP035.sqn | CESAU12 | KF285098 |
| ACECP035.sqn | CESAU13 | KF285099 |
| ACECP035.sqn | CESAU14 | KF285100 |
| ACECP035.sqn | CESAU15 | KF285101 |
| ACECP035.sqn | CESAU16 | KF285102 |
| ACECP035.sqn | CESAU17 | KF285103 |
| ACECP035.sqn | CESAU20 | KF285104 |
| ACECP035.sqn | CESAU22 | KF285105 |
| ACECP035.sqn | CESAU23 | KF285106 |
| ACECP035.sqn | CESBD01 | KF285107 |
| ACECP035.sqn | CESEI01 | KF285108 |
| ACECP035.sqn | CESEI02 | KF285109 |
| ACECP035.sqn | CESET01 | KF285110 |
| ACECP035.sqn | CESHW01 | KF285111 |
| ACECP035.sqn | CESIN01 | KF285112 |
| ACECP035.sqn | CESIN02 | KF285113 |
| ACECP035.sqn | CESIN03 | KF285114 |
| ACECP035.sqn | CESIN06 | KF285115 |
| ACECP035.sqn | CESIN07 | KF285116 |
| ACECP035.sqn | CESIN08 | KF285117 |
| ACECP035.sqn | CESIN10 | KF285118 |
| ACECP035.sqn | CESIN11 | KF285119 |
| ACECP035.sqn | CESIN12 | KF285120 |
| ACECP035.sqn | CESJP01 | KF285121 |
| ACECP035.sqn | CESJP02 | KF285122 |
| ACECP035.sqn | CESJP03 | KF285123 |
| ACECP035.sqn | CESJP04 | KF285124 |
| ACECP035.sqn | CESJP05 | KF285125 |
| ACECP035.sqn | CESJP06 | KF285126 |
| ACECP035.sqn | CESJP07 | KF285127 |
| ACECP035.sqn | CESJP08 | KF285128 |
| ACECP035.sqn | CESJP09 | KF285129 |
| ACECP035.sqn | CESJP10 | KF285130 |
| ACECP035.sqn | CESJP11 | KF285131 |
| ACECP035.sqn | CESJP12 | KF285132 |
| ACECP035.sqn | CESJP13 | KF285133 |
| ACECP035.sqn | CESJP14 | KF285134 |
| ACECP035.sqn | CESJP15 | KF285135 |
| ACECP035.sqn | CESJP16 | KF285136 |
| ACECP035.sqn | CESJP17 | KF285137 |
| ACECP035.sqn | CESJP18 | KF285138 |
| ACECP035.sqn | CESJP19 | KF285139 |
| ACECP035.sqn | CESJP20 | KF285140 |
| ACECP035.sqn | CESJP21 | KF285141 |
| ACECP035.sqn | CESJP22 | KF285142 |
| ACECP035.sqn | CESJP23 | KF285143 |
| ACECP035.sqn | CESJP24 | KF285144 |
| ACECP035.sqn | CESJP25 | KF285145 |
| ACECP035.sqn | CESJP26 | KF285146 |
| ACECP035.sqn | CESJP27 | KF285147 |
| ACECP035.sqn | CESJP28 | KF285148 |
| ACECP035.sqn | CESJP29 | KF285149 |
| ACECP035.sqn | CESLK02 | KF285150 |
| ACECP035.sqn | CESLK03 | KF285151 |
| ACECP035.sqn | CESLK04 | KF285152 |
| ACECP035.sqn | CESMG01 | KF285153 |
| ACECP035.sqn | CESMG02 | KF285154 |
| ACECP035.sqn | CESMG03 | KF285155 |
| ACECP035.sqn | CESMG04 | KF285156 |
| ACECP035.sqn | CESMM02 | KF285157 |
| ACECP035.sqn | CESMM03 | KF285158 |
| ACECP035.sqn | CESMM06 | KF285159 |
| ACECP035.sqn | CESMM07 | KF285160 |
| ACECP035.sqn | CESMM08 | KF285161 |
| ACECP035.sqn | CESMM09 | KF285162 |
| ACECP035.sqn | CESMM10 | KF285163 |
| ACECP035.sqn | CESMM11 | KF285164 |
| ACECP035.sqn | CESMM12 | KF285165 |
| ACECP035.sqn | CESMM13 | KF285166 |
| ACECP035.sqn | CESNP02 | KF285167 |
| ACECP035.sqn | CESNP03 | KF285168 |
| ACECP035.sqn | CESNZ04 | KF285169 |
| ACECP035.sqn | CESNZ05 | KF285170 |
| ACECP035.sqn | CESNZ06 | KF285171 |
| ACECP035.sqn | CESNZ07 | KF285172 |
| ACECP035.sqn | CESNZ08 | KF285173 |
| ACECP035.sqn | CESNZ09 | KF285174 |
| ACECP035.sqn | CESNZ10 | KF285175 |
| ACECP035.sqn | CESNZ11 | KF285176 |
| ACECP035.sqn | CESNZ12 | KF285177 |
| ACECP035.sqn | CESNZ13 | KF285178 |
| ACECP035.sqn | CESNZ15 | KF285179 |
| ACECP035.sqn | CESNZ16 | KF285180 |
| ACECP035.sqn | CESNZ17 | KF285181 |
| ACECP035.sqn | CESNZ18 | KF285182 |
| ACECP035.sqn | CESNZ19 | KF285183 |
| ACECP035.sqn | CESNZ20 | KF285184 |
| ACECP035.sqn | CESNZ21 | KF285185 |
| ACECP035.sqn | CESNZ22 | KF285186 |
| ACECP035.sqn | CESNZ23 | KF285187 |
| ACECP035.sqn | CESPG01 | KF285188 |
| ACECP035.sqn | CESPG02 | KF285189 |
| ACECP035.sqn | CESPG03 | KF285190 |
| ACECP035.sqn | CESPG04 | KF285191 |
| ACECP035.sqn | CESPG05 | KF285192 |
| ACECP035.sqn | CESPG06 | KF285193 |
| ACECP035.sqn | CESPG07 | KF285194 |
| ACECP035.sqn | CESPG08 | KF285195 |
| ACECP035.sqn | CESPG09 | KF285196 |
| ACECP035.sqn | CESPG10 | KF285197 |
| ACECP035.sqn | CESPG11 | KF285198 |
| ACECP035.sqn | CESPG12 | KF285199 |
| ACECP035.sqn | CESPG13 | KF285200 |
| ACECP035.sqn | CESPG14 | KF285201 |
| ACECP035.sqn | CESPG15 | KF285202 |
| ACECP035.sqn | CESPG16 | KF285203 |
| ACECP035.sqn | CESPG17 | KF285204 |
| ACECP035.sqn | CESPG18 | KF285205 |
| ACECP035.sqn | CESPG19 | KF285206 |
| ACECP035.sqn | CESPH01 | KF285207 |
| ACECP035.sqn | CESPH02 | KF285208 |
| ACECP035.sqn | CESPH03 | KF285209 |
| ACECP035.sqn | CESPH04 | KF285210 |
| ACECP035.sqn | CESPH05 | KF285211 |
| ACECP035.sqn | CESPH06 | KF285212 |
| ACECP035.sqn | CESPH07 | KF285213 |
| ACECP035.sqn | CESPH08 | KF285214 |
| ACECP035.sqn | CESPH09 | KF285215 |
| ACECP035.sqn | CESPK01 | KF285216 |
| ACECP035.sqn | CESPK02 | KF285217 |
| ACECP035.sqn | CESPK05 | KF285218 |
| ACECP035.sqn | CESPK06 | KF285219 |
| ACECP035.sqn | CESPK07 | KF285220 |
| ACECP035.sqn | CESSI01 | KF285221 |
| ACECP035.sqn | CESSI02 | KF285222 |
| ACECP035.sqn | CESSI03 | KF285223 |
| ACECP035.sqn | CESTH01 | KF285224 |
| ACECP035.sqn | CESTH03 | KF285225 |
| ACECP035.sqn | CESTH04 | KF285226 |
| ACECP035.sqn | CESTH05 | KF285227 |
| ACECP035.sqn | CESTL01 | KF285228 |
| ACECP035.sqn | CESVN01 | KF285229 |
| ACECP035.sqn | CESVN02 | KF285230 |
| ACECP035.sqn | CESVN03 | KF285231 |
| ACECP035.sqn | CESVN04 | KF285232 |
| ACECP035.sqn | CESVN05 | KF285233 |
| ACECP035.sqn | CESVN06 | KF285234 |
| ACECP035.sqn | CESVN07 | KF285235 |
| ACECP035.sqn | CESVN08 | KF285236 |
| ACECP035.sqn | CESVN09 | KF285237 |
| ACECP035.sqn | CESVN10 | KF285238 |
| ACECP035.sqn | CESVN11 | KF285239 |
| ACECP035.sqn | CESVN12 | KF285240 |
| ACECP035.sqn | CESVN13 | KF285241 |
| ACECP035.sqn | CESVN14 | KF285242 |
| ACECP035.sqn | CFAIN01 | KF285243 |
| ACECP035.sqn | CFOPH01 | KF285245 |
| ACECP035.sqn | CFOTW02 | KF285246 |
| ACECP035.sqn | CLIVN01 | KF285250 |
| ACECP035.sqn | CLIVN02 | KF285251 |
| ACECP035.sqn | CLIVN03 | KF285252 |
| ACECP035.sqn | CMEVN01 | KF285253 |
| ACECP035.sqn | CMEVN02 | KF285254 |
| ACECP035.sqn | CMEVN03 | KF285255 |
| ACECP035.sqn | CSPCY01 | KF285256 |
| ACECP035.sqn | CSPFJ01 | KF285257 |
| ACECP035.sqn | CSPFJ02 | KF285258 |
| ACECP035.sqn | CSPFJ03 | KF285259 |
| ACECP035.sqn | CSPFJ05 | KF285261 |
| ACECP035.sqn | CSPIN01 | KF285263 |
| ACECP035.sqn | CSPMM01 | KF285264 |
| ACECP035.sqn | CSPMM02 | KF285265 |
| ACECP035.sqn | CSPVN01 | KF285266 |
| ACECP035.sqn | CSPVN02 | KF285267 |
| ACECP035.sqn | CSPVN03 | KF285268 |
| ACECP035.sqn | CSPVN04 | KF285269 |
| ACECP035.sqn | CSPVN05 | KF285270 |
| ACECP035.sqn | CYUVN01 | KF285271 |
| ACECP035.sqn | CYUVN02 | KF285272 |
| ACECP035.sqn | CYUVN03 | KF285273 |
| ACECP035.sqn | CYUVN04 | KF285274 |
| ACECP035.sqn | CYUVN05 | KF285275 |
| ACECP035.sqn | RSPMM01 | KF285276 |
| ACECP035.sqn | RSPVN01 | KF285277 |
| ACECP035.sqn | SSPVN01 | KF285278 |
| ACECP005.sqn | CESPK03 | JN105395 |
| ACECP005.sqn | CESAU18 | JN105396 |
| ACECP005.sqn | CFOTW01 | JN105398 |
| ACECP005.sqn | CESPK04 | JN105400 |
| ACECP005.sqn | CESNZ02 | JN105401 |
| ACECP005.sqn | CESNZ03 | JN105402 |
| ACECP005.sqn | CESAU10 | JN105403 |
| ACECP005.sqn | CESJP01 | JN105404 |
| ACECP005.sqn | CESNZ14 | JN105405 |
| ACECP005.sqn | CESNZ01 | JN105406 |
| ACECP005.sqn | CAFAU01 | JN105407 |
| ACECP016.sqn | CAFAU01 | JN105448 |
| ACECP016.sqn | CFOTW01 | JN105449 |
| ACECP016.sqn | CESNZ02 | JN105450 |
| ACECP016.sqn | CESAU10 | JN105451 |
| ACECP016.sqn | CESPK03 | JN105452 |
| ACECP016.sqn | CESPK04 | JN105453 |
| ACECP016.sqn | CESNZ03 | JN105454 |
| ACECP016.sqn | CESJP01 | JN105455 |
| ACECP016.sqn | CESNZ14 | JN105456 |
| ACECP016.sqn | CESNZ04 | JN105457 |
| ACECP018.sqn | CESPK03 | JN105473 |
| ACECP018.sqn | CESPK04 | JN105474 |
| ACECP018.sqn | CESNZ02 | JN105475 |
| ACECP018.sqn | CESAU18 | JN105476 |
| ACECP018.sqn | CESAU10 | JN105477 |
| ACECP018.sqn | CFOTW01 | JN105478 |
| ACECP018.sqn | CAFAU01 | JN105482 |
| ACECP018.sqn | CESJP01 | JN105483 |
| ACECP018.sqn | CESNZ14 | JN105484 |
| ACECP018.sqn | CESNZ04 | JN105485 |
| ACECP018.sqn | CESNZ03 | JN105486 |
| ACECP026.sqn | CESPK03 | JN105543 |
| ACECP026.sqn | CAFAU01 | JN105544 |
| ACECP026.sqn | CESAU18 | JN105545 |
| ACECP026.sqn | CESNZ02 | JN105546 |
| ACECP026.sqn | CFOTW01 | JN105547 |
| ACECP026.sqn | CESPK04 | JN105549 |
| ACECP026.sqn | CESNZ01 | JN105551 |
| ACECP026.sqn | CESAU10 | JN105552 |
| ACECP026.sqn | CESNZ03 | JN105556 |
| ACECP026.sqn | CESNZ14 | JN105557 |
| ACECP026.sqn | CESJP01 | JN105558 |
| ACECP035.sqn | CAFAU01 | JN105588 |
| ACECP035.sqn | CFOTW01 | JN105589 |
| ACECP035.sqn | CESNZ02 | JN105590 |
| ACECP035.sqn | CESPK03 | JN105591 |
| ACECP035.sqn | CESPK04 | JN105592 |
| ACECP035.sqn | CESAU18 | JN105593 |
| ACECP035.sqn | CESAU10 | JN105594 |
| ACECP035.sqn | CESNZ01 | JN105597 |
| ACECP035.sqn | CESNZ14 | JN105598 |
| ACECP035.sqn | CESNZ03 | JN105599 |
| ACECP039.sqn | CAFAU01 | JN105622 |
| ACECP039.sqn | CESJP01 | JN105623 |
| ACECP039.sqn | CESNZ14 | JN105624 |
| ACECP039.sqn | CESNZ01 | JN105625 |
| ACECP039.sqn | CESNZ03 | JN105626 |
| ACECP039.sqn | CESPK04 | JN105627 |
| ACECP039.sqn | CESNZ02 | JN105628 |
| ACECP039.sqn | CESPK03 | JN105629 |
| ACECP039.sqn | CESAU10 | JN105630 |
| ACECP039.sqn | CESAU18 | JN105631 |
| ACECP039.sqn | CFOTW01 | JN105632 |

**Supporting Table 3. Chloroplast haplotype groups (Types 1–14) in *Colocasia* species.**

Grouping of 185 samples (*C. esculenta* and other *Colocasia* species) into 14 haplotypes. For each haplotype, we show clade (CI-III), species, countries of collection, and sample identification codes. Samples in bold displayed sequences with incomplete data, but could be unequivocally assigned, manually, to the haplotype groups. Unique haplotypes are identified in Fig. 3 and Supp. Figs 1, 2 by their sample identification codes, and are noted as unique in the results for individual samples in Supp. Table 1.

| **Haplotype** | **Species** | **Country** | **Sample codes** |
| --- | --- | --- | --- |
| TYPE1 (CI) | *Colocasia esculenta* | Australia | CESAU04, CESAU07, CESAU20 |
|  |  | Easter Island, Chile | CESEI01, CESEI02, **CESEI03** |
|  |  | Egypt | CESJP02 |
|  |  | Hawaii, USA | **CESHW01** |
|  |  | India | CESIN03, CESIN06, **CESIN09** |
|  |  | Japan | CESJP01, CESJP05, CESJP09, CESJP10, CESJP11, CESJP14, CESJP15, CESJP16, CESJP17, CESJP18, CESJP19, CESJP20, CESJP21, CESJP22, CESJP23, CESJP24, CESJP25, CESJP26, CESJP27, CESJP28, CESJP29 |
|  |  | Madagascar | CESMG04 |
|  |  | Myanmar | **CESMM11** |
|  |  | New Zealand | CESNZ01,CESNZ13, CESNZ14, CESNZ15, CESNZ20 |
|  |  | New Guinea | CESPG02, CESPG03, **CESPG04,** CESPG05, CESPG08, CESPG09, CESPG10, CESPG11, CESPG12, CESPG13, CESPG14, CESPG15, CESPG16, CESPG17, **CESPG18,** CESPG19 |
|  |  | Philippines | CESPH01, CESPH02, CESPH03, CESPH04, CESPH05, CESPH09 |
|  |  | Society Islands, French Polynesia | CESSI01, CESSI02, CESSI03 |
|  |  | Thailand | CESTH04, CESTH05 |
|  |  | Vietnam | CESVN01, CESVN04, CESVN05, CESVN08, CESVN09, CESVN12, CESVN14 |
|  |  | Cyprus | CSPCY01 |
|  |  | Fiji | CSPFJ01, CSPFJ02, CSPFJ03, **CSPFJ05** |
|  | *C. affinis* | Philippines | CAFPH01 |
| TYPE2 (CIII) | *C. esculenta* | Australia | CESAU01, CESAU03, CESAU05, **CESAU06, CESAU09, CESAU21,** CESAU23 |
|  |  | New Guinea | CESPG01, CESPG06, CESPG07 |
| TYPE3 (CIII) | *C. esculenta* | Australia | CESAU08, CESAU10, CESAU11, CESAU12, CESAU13, CESAU14, CESAU15, CESAU16, **CESAU17, CESAU18,** CESAU19, CESAU22 |
| TYPE4 (CI) | *C. esculenta* | Bangladesh | CESBD01 |
|  |  | India | CESIN02, CESIN07, CESIN08, CESIN11, CESIN12 |
|  |  | Sri Lanka | CESLK02 |
|  |  | Myanmar | CESMM03, CESMM09, CESMM13 |
|  |  | New Zealand | CESNZ03, CESNZ08, CESNZ16 |
|  |  | Timor-Leste | CESTL01 |
|  |  | Vietnam | CESVN06 |
|  | *Colocasia* sp*.* cf. *affinis* | Myanmar | CSPMM01, CSPMM02 |
| TYPE5 (CII) | *C. esculenta* | Ethiopia | CESET01 |
|  |  | Japan | CESJP03, CESJP04, CESJP06, **CESJP07,** CESJP08, CESJP12, CESJP13 |
|  |  | Sri Lanka | CESLK04 |
|  |  | Madagascar | CESMG01, CESMG02, CESMG03 |
|  |  | Nepal | CESNP03 |
|  |  | New Zealand | CESNZ02, CESNZ05, CESNZ06, CESNZ07, CESNZ09, CESNZ11, CESNZ12, CESNZ17, CESNZ18, CESNZ19, CESNZ21 |
|  |  | Pakistan | CESPK03, CESPK04 |
| TYPE6 (CIII) | *C. esculenta* | Myanmar | CESMM02, CESMM10 |
| TYPE7 (CII) | *C. esculenta* | Nepal | CESNP01 |
|  |  | Pakistan | CESPK01, CESPK02, CESPK05, CESPK06, CESPK07 |
| TYPE8 (CII) | *C. esculenta* | New Zealand | CESNZ22, CESNZ23 |
| TYPE9 (CI) | *C. esculenta* | Philippines | CESPH06, CESPH07, CESPH08 |
| TYPE10 (CIII) | *C. esculenta* | Thailand | CESTH01, **CESTH02,** CESTH03 |
| TYPE11(CIII) | *C. esculenta* | Vietnam | CESVN02, CESVN03, CESVN07, CESVN10, CESVN13 |
|  | *C. lihengiae* | Vietnam | CLIVN03 |
|  | *C. menglaensis* | Vietnam | CMEVN03 |
|  | *Colocasia* sp*.* | Vietnam | CSPVN01, CSPVN02, CSPVN03, CSPVN04 |
|  | *C. yunnanensis* | Vietnam | CYUVN01, CYUVN02, CYUVN03, CYUVN04, CYUVN05 |
| TYPE12 (CIII) | *C. esculenta* | Vietnam | CESVN11 |
|  | *C. lihengiae* | Vietnam | CLIVN01, CLIVN02 |
|  | *C. menglaensis* | Vietnam | CMEVN01, CMEVN02 |
| TYPE13 (CIII) | *C. formosana* | Taiwan, China | CFOTW01, CFOTW02 |
| TYPE14 (CIII) | *C. esculenta* | Myanmar | **CESMM06,** CESMM08 |

1. Taxonomic authorities for species and varieties mentioned in this table are:

   *Remusatia vivipara* (Roxb.) Schott, *Steudnera* K. Koch, *Colocasia* Schott, *C. affinis* Schott, *C. affinis* var. *jenningsii* (Veitch) Engl., *C. fallax* Schott, *C. formosana* Hayata, *C. lihengiae* Long & Liu, *C. menglaensis* Yin, Li & Xu, *C. yunnanensis* Long & Cai, *C. esculenta* (L.) Schott, *C. esculenta* var. *fontanesii* (Schott) A. F. Hill. [↑](#footnote-ref-1)
2. *Source country* usually means the country of sample collection, but in some cases there was certain information that the sampled plant had been recently introduced from another named country. [↑](#footnote-ref-2)
3. *Sample ID* = species abbreviation (first three letters), country code (two letters), and individual sample number (two digits). [↑](#footnote-ref-3)
4. *Remarks* – *via* or *ex* indicate samples not directly derived from the stated field source. *Collector initials* refer to the following collectors: E. Maribel Agoo (EMA), Ibrar Ahmed (IA), V. Amarasinghe (VA), Josef Bogner (JB), Harold Conklin (HC), Wojciech Dabrowski (WD), Rhys Jones (RJ), Trevor King (TK), Peter Latz (PL), Ian Lawlor (IL), Peter J. Matthews (PJM), Dilip K. Medhi (DKM), Kyaw W. Naing (KWN), K. Ikeya (KI), K. Ikezawa (KIk), Sadao Sakamoto (SS), Nguyen Kien (NK), Nguyen V. Dzu (NVD), Joycelyn Powell (JP), Neville Scarlett (NS), Etsuko Tabuchi (ET), Emiko Takei (ETK), Venesa Tanner (VT), Neville White (NW), HEW (Henry E. Wright), DEY (Douglas E. Yen), T. Yoshino (TY). Chromosome numbers or ploidy are indicated if known from direct counts on the sample plant (‘2*n* =’), or published descriptions of specific varieties or populations (‘diploid/triploid’). [↑](#footnote-ref-4)
5. *Other numbers* include collection numbers, voucher specimen numbers in herbaria, etc., as follows. *ABG* refers to Auckland Botanic Gardens, Manurewa. *ANU T* refers to a living collection maintained at the Australian National University, Canberra, by D. E. Yen and P. J. Matthews (Canberra Taro Collection, 1981–1989) (Matthews, 2014). *KPGI* refers to Kyoto Plant Germplasm Institute (Kyoto University), Mozume-cho, Kyoto. LA L- refers to the living collection of Lyon Arboretum (LA), Honolulu, Hawaii. *MPN* refers to a voucher specimen in the herbarium of Massey University, Palmerston North, New Zealand. *UAAR* refers to the herbarium of Pir Mehr Ali Shah, University of Arid Agriculture, Rawalpindi, Pakistan. *WP* refers to waypoint locations recorded with a GPS device (Matthews, fieldnotes). [↑](#footnote-ref-5)
6. In this table, ‘wild’ refers to either an apparently natural wild habitat, to the obviously modified habitat of a commensal wild population, or to a known cultivar that has naturalized. For many samples, the original collectors did not describe the source habitat in sufficient detail to distinguish ‘apparently natural’ from ‘obviously modified and commensal’. In a few instances, the status of a wild plant as a naturalized cultivar was obvious at the time of collection, or can be inferred because it is a known exotic cultivar (e.g. var. RR in New Zealand; Matthews, 1985, 2014). Plants collected as corms from a market or described by the collector as cultivar, are identified here as cultivars. Plants identified here as ‘ornamental’ may be domesticated forms or may be wildtypes that have entered cultivation and the ornamental trade. [↑](#footnote-ref-6)
7. All samples collected from the Queensland wild population (CESAU02-CESAU23) are phenotypically uniform, and are known or likely to be diploids, cf. ‘Jiyer phenotype’ (Matthews, 2014). This population as a whole does not appear commensal, and many samples came from apparently natural habitats (e.g., Fig. 2d). All New Zealand samples identified as var. RR and var. GP are identified here as triploid on the basis of cytological and ribosomal DNA surveys conducted in the 1980s (Matthews, 2014). Those surveys indicated uniformity in the phenotypes and nuclear genomes of plants identified on the basis of morphology and leaf color; var. RR was both wild (commensal) and in cultivation, while var. GP was consistently wild and commensal. All Okinawa samples in the sequence CESJP14-29 belong to a commensal wild variety that is diploid (Matthews, Takei, & Kawahara, 1992). [↑](#footnote-ref-7)
